# The largest hoplophonine and a complex new hypothesis of nimravid evolution

**DOI:** 10.1038/s41598-021-00521-1

**Published:** 2021-10-26

**Authors:** Paul Zachary Barrett

**Affiliations:** Department of Earth Sciences, University of Oregon, Eugene, OR 97403 USA

**Keywords:** Ecology, Evolution, Zoology, Ecology

## Abstract

Nimravids were the first carnivorans to evolve saberteeth, but previously portrayed as having a narrow evolutionary trajectory of increasing degrees of sabertooth specialization. Here I present a novel hypothesis about the evolution of this group, including a description of *Eusmilus adelos*, the largest known hoplophonine, which forces a re-evaluation of not only their relationships, but perceived paleoecology. Using a tip-dated Bayesian analysis with sophisticated evolutionary models, nimravids can now be viewed as following two paths of evolution: one led to numerous early dirk-tooth forms, including *E. adelos*, while the other converged on living feline morphology, tens of millions of years before its appearance in felids.

## Introduction

Since their initial discovery, specimens that are now referred to the Nimravidae have been likened to, or indeed placed within, the Felidae, living cats and their numerous extinct sabertooth relatives. These comparisons resulted in the moniker of ‘false sabertooth cats’ for this group of carnivores, which until recently was thought to represent one of three independent acquisitions of sabertooth morphology within Carnivoramorpha. These studies have subsumed the “barbourofelids” into Nimravidae, as Miocene members of this family, based upon shared basicranial morphology, but the precise placement of these Miocene nimravids is still debated^1–3^.

All past phylogenetic analyses have cast these non-felid sabertooths as having an evolutionary history of increased acquisition of sabertooth morphology, resulting in pectinate relationships of the least sabertoothed taxa to the most^1,3–8^. This pattern may stem from a historical interest and resultant character selection of these sabertooth features, focusing on the dental aspects of nimravid anatomy, and the necessary cranial adaptations to wield saberteeth. However, as has been shown by recent analyses^2,9–12^, these morphological features are highly convergent in sabertooth mammals, which begs the question, would increase sampling of non-sabertooth characters affect interpretation of evolutionary relationships? By bringing together the most-comprehensive set of nimravid taxa yet analyzed, including *Eusmilus adelos* sp. nov., the largest known hoplophonine, I report a novel hypothesis of nimravid evolutionary history. This hypothesis forces a re-evaluation of nimravid evolution as an escalator towards a single, hyperspecialized sabertooth ecomorphology, instead positing a diverse array of derived forms analogous to modern felids, but occurring tens of millions of years earlier. This hypothesis stems from a near six-fold increase in morphological characters, sampled tip-date priors, and sophisticated models of character evolution brought together in a Bayesian framework.

## Results

### Systematic palaeontology

Carnivora^13^.

Nimravidae^14^.

*Eusmilus*^15^.

*Eusmilus adelos *sp. nov.

ZooBank LSID (for nomenclatural act): AE752634-8697-473C-8CD6-CA301A8E56A8

**Etymology **From the Greek *adelos*, for unseen, unknown, or secret. The specific epithet refers to the unclear taxonomic affiliations these specimens have had in their more than 85 year history of publication^4,16–20^.

**Figure 1:**
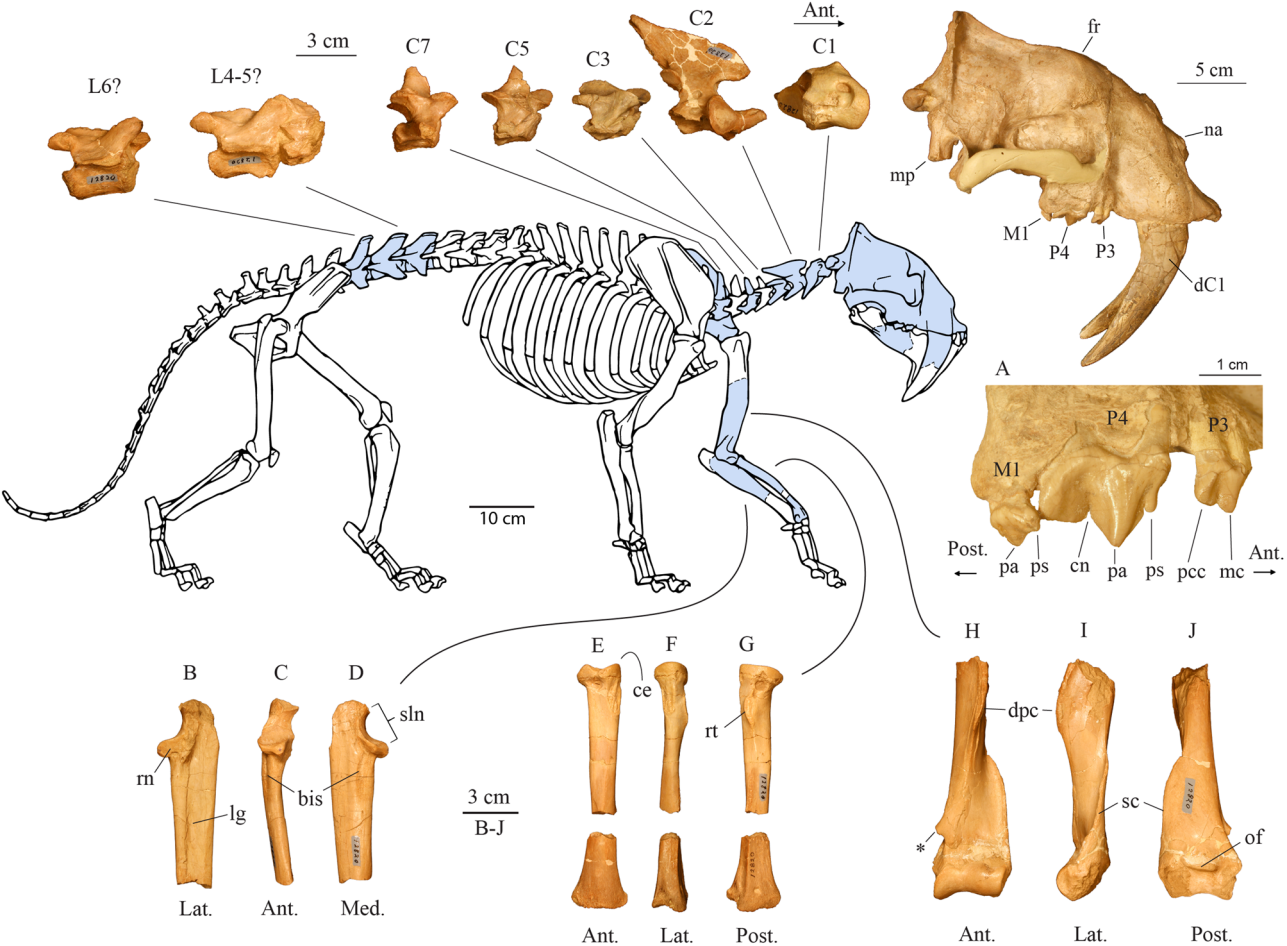
Partial skeleton of USNM 12820, *Eusmilus adelos* with shaded known elements. Cranial abbreviations: *fr* frontal, *na* nasal, *mp* mastoid process, (**A**) *cn* carnassial notch, *mc* main cusp of P3, *pa* paracone, *pcc* posterior cingular cusp of P3, *ps* parastyle; (**B**–**D**) *bis* brachialis insertion site, *lg* lateral groove of ulna, *rn* radial notch, *sln* semilunar notch, (**E**–**G**) *ce* capitular eminence of radius, *rt* radial tuberosity, (**H**–**J**) *dpc* delto-pectoral crest, *of* olecranon fossa, *sc* supinator crest (brachial flange), *remnants of bridge enclosing epicondylar foramen. *Eusmilus adelos* skeletal reconstruction by Dhruv Franklin.

**Figure 2:**
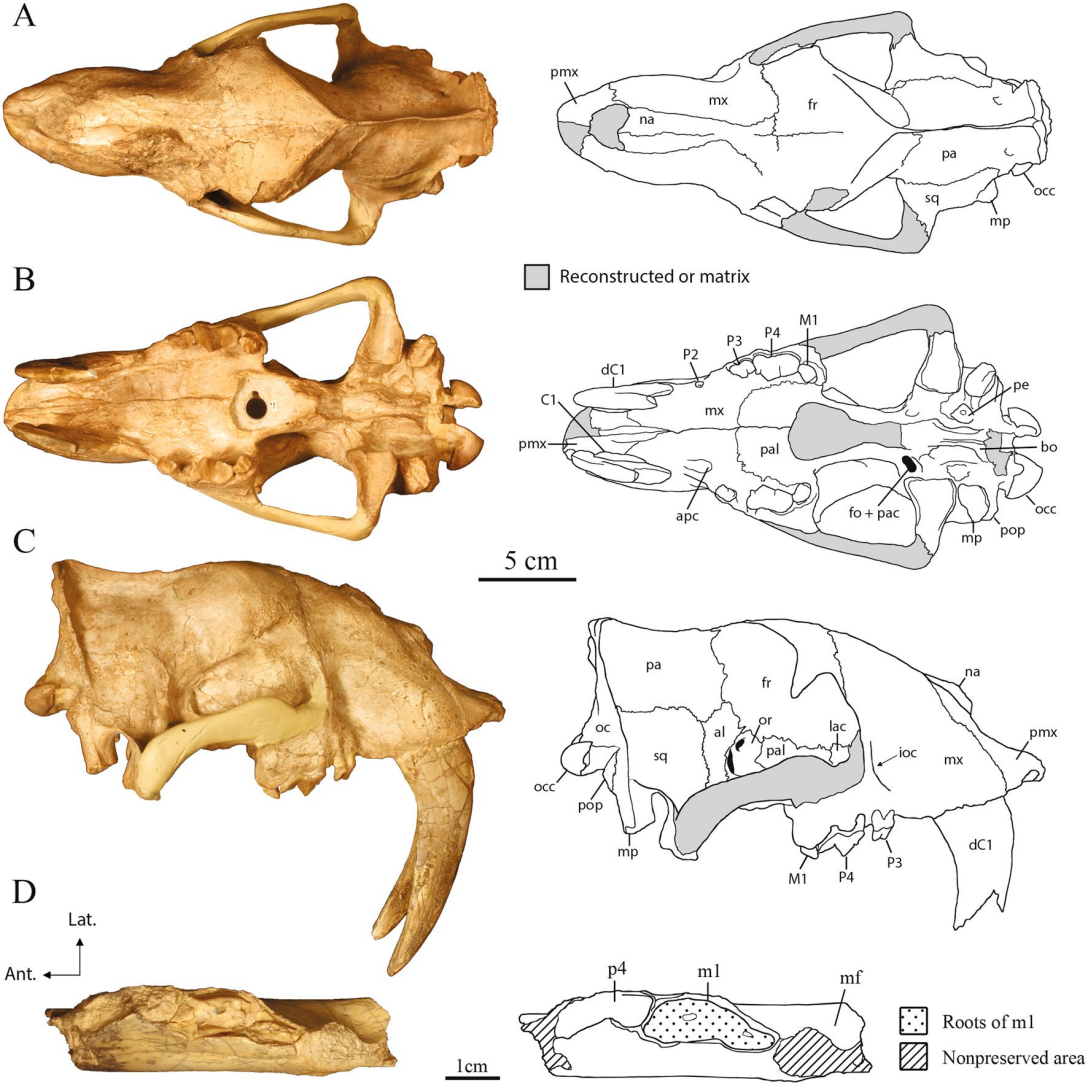
Cranium and dentary of USNM 12820, *Eusmilus adelos*, sp. nov. in dorsal (**A**), ventral (**B**), right lateral (**C**) and occlusal (**D**) views. Abbreviations: *al* alisphenoid, *apc* palatine canal anterior opening, *bo* basioccipital, *fo* foramen ovale, *fr* frontal, *ioc* infraorbital canal, *lac* lacrimal, *mf* masseteric fossa, *mp* mastoid process, *mx* maxilla, *na* nasal, *oc* occipital, *occ* occipital condyle, *or* orbitosphenoid, *pa* parietal, *pac* alisphenoid canal posterior opening, *pal* palatine, *pe* petrosal, *pmx* premaxilla, *pop* paroccipital process, *sq* squamosal.

**Figure 3:**
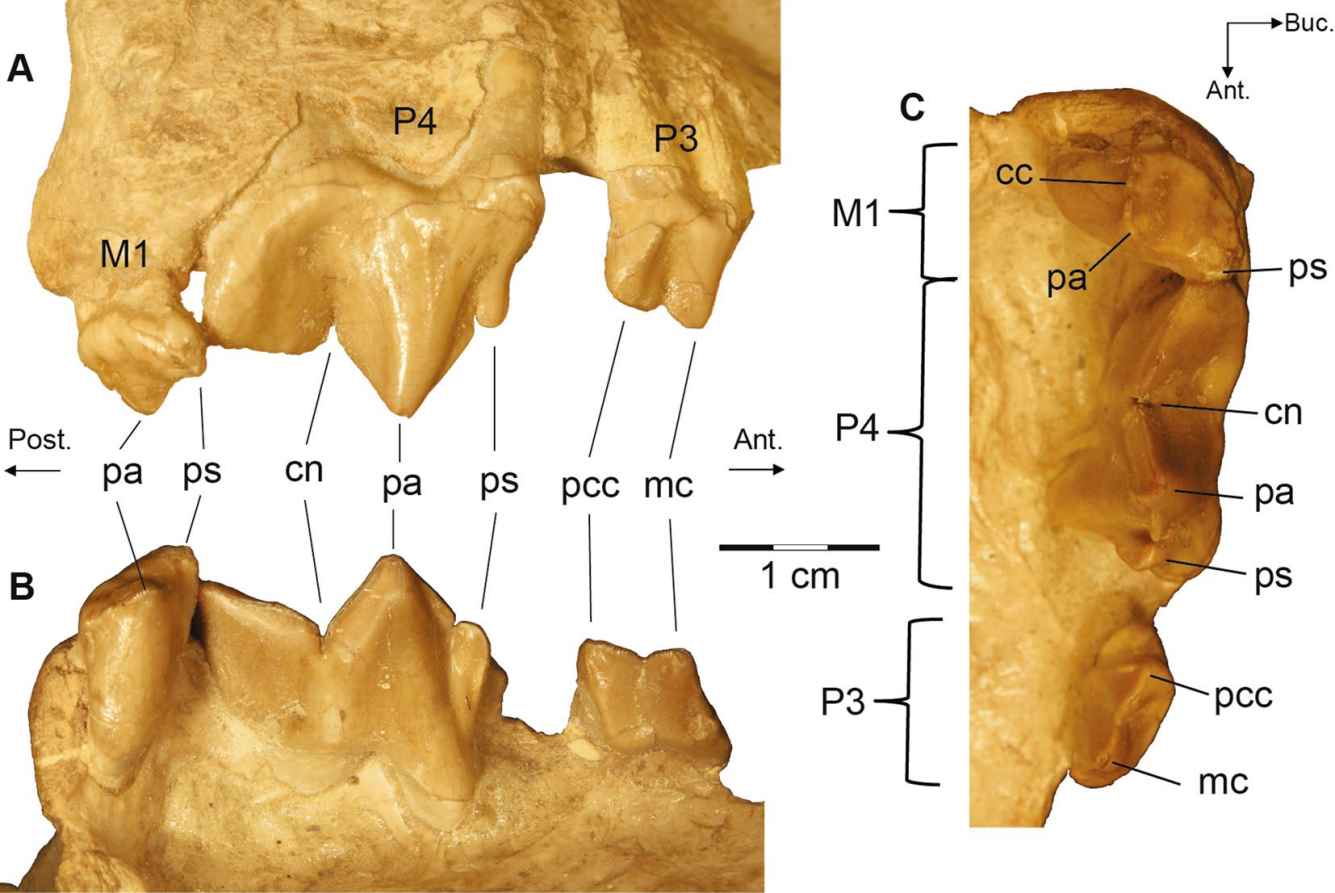
Posterior dentition of USNM 12820, *Eusmilus adelos*, sp. nov., in buccal (**A**), lingual (**B**) and occlusal (**C**) views. Note the anomalous gap between the P3 and P4 not seen in the left tooth row, nor in USNM 18214. *cc* centrocrista, *cn* carnassial notch, *mc* main cusp of P3, *pa* paracone, *pcc* posterior cingular cusp of P3, *ps* parastyle.

**Holotype** Smithsonian National Museum of Natural History, Washington D.C., USA (USNM) 12820: partially crushed cranium, parts of both dentaries, atlas, axis, 3rd, 5th and 7th cervical vertebrae, three lumbar vertebrae, left: scapula, distal humerus, proximal and distal ends of radius, proximal ulna (Figs. 1, 2, 3, Supplementary Figs. S1–S3).

**Referred material** Paratype: USNM 18214, cranium, Supplementary Fig. S1.

**Locality and horizon** Orellan of Wyoming (White River Fm., Brule Mbr.), Niobrara County, USA.

**Diagnosis** Characters of *Eusmilus* (see Supplementary Information and Barrett^6^) plus: C1 serration density of 5.58 denticles per millimeter, compared to *E. cerebralis* with 6.2 and *Hoplophoneus primaevus* of 4.4^6^; hypoglossal foramen within groove between the occipital condyle and the paroccipital process; the nasals are short compared to all other nimravid taxa save *Barbourofelis fricki* and *Eusmilus cerebralis*, where the posterior border lies across or anterior to the maxillofrontal suture; dorsal jugal-squamosal suture in zygomatic arch does not abut the postorbital process, unlike all other nimravid taxa except *Eusmilus cerebralis*.

**General description** The cranial morphology of both specimens is comparable to that of other *Hoplophoneus* and *Eusmilus* species (e.g., *Hoplophoneus primaevus* and *Eusmilus sicarius*), with hypertrophied upper canines, reduction of pre- and postcarnassial dentition, large mastoid processes, and ventral projection of the glenoid pedicles. In dorsal view (Fig. 2A, Supplementary Fig. S1a,e) the nasals are shortened, such that the posterior margin does not extend beyond the maxillofrontal suture, a condition that otherwise is only known in the “toy sabertooth” *Eusmilus cerebralis* and the exceedingly derived, late Miocene *Barbourofelis fricki*. The postorbital constriction occurs along the frontal-parietal suture with the frontals being slightly longer than the parietals.

In ventral view (Fig. 2B, Supplementary Fig. S1b,f) the palate is triangular with the width between the canines less than that at the P4s. The upper canine is anteroposterior longer than that of the P4—33.2 mm (dC1) versus 21.3 mm (values from USNM 12820)—and separated from the P3 by a diastema similar in length to that of the canine. A C1 that is longer than the P4 is only found in members of the genus *Eusmilus* and in *Pogonodon platycopis* and *Hoplophoneus oharrai*. A diminutive P2 (length 3.82 mm) is present on the left side of USNM 12820, but absent on the right and not present at all on USNM 18214. The P3 is double rooted, lacking an anterior cusp, but possessing a single posterior cingular cusp. The P4 (Fig. 3) is trenchant, with a prominent paracone and metastylar blade. A parastyle cusp, emanating from the anterior face of the paracone, is present with a crista positioned posterior to it and ventrally inclined, creating a notch. The M1 is reduced, lacking a protocone and metacone, with a centrocrista extending to the middle posterior of the tooth. A large, mounded paracone is present, with a sinusoidal paracrista connecting it to an anterobuccal directed parastyle shelf. The anterior palatine canal opens through the maxilla, anterior of the P3, while the posterior margin of the tooth row extends beyond the posterior margin of the medial palate. The foramen ovale is located at the anterior margin of the glenoid fossa, and is joined in a common depression with the posterior entrance of the alisphenoid canal. The basicranial region is heavily buttressed with ventral projecting basioccipital flanges, suggesting a large attachment area for non-preserved bullar elements. The roof and medial wall of the petrobasilar canal for the inferior petrosal venous sinus is formed entirely by the basioccipital, whereas the petrosal contacts the basioccipital and contributes to the lateral wall of the canal along its preserved portion. The paroccipital process is simple and short, exhibiting a sutural contact with the mastoid process that travels into the auditory region. The petrobasilar foramen is aligned with this suture on the medial side of the auditory capsule, and is confluent with the petrobasilar canal. A large hypoglossal foramen is located posterior to the petrobasilar foramen, though separated by the basioccipital, within a groove between the occipital condyle and paroccipital process.

In lateral view (Figs. 1, 2C; Supplementary Fig. S1c,d,g,h), the premaxillary dental arcade exhibits a significant amount of prognathism, with I1 and I2 extending more anteriorly than the I3. The infraorbital foramen is large and placed close to the antero-ventral corner of the orbit, above the center of the P3. The occiput is anteriorly rotated, creating an angle between the lambdoid crest and the cingular border of the cheektooth row of approximately 90°, compared to a mean value of 116 for *Hoplophoneus primaevus*^6^.

Portions of the left and right dentaries (Fig. 2D) are preserved in USNM 12820, but no teeth are present in either dentary. However, judging from the alveoli, the p4 would have been large (length 19.39 mm; width 6.51 mm, measurements for right side). Given the orientation of the roots, this tooth in life would have presented significant lateral rotation, a condition noted in several nimravid taxa^21^. The p4 is imbricated to the m1, with the p4 lateral. The m1 of both sides preserve portions of base of the crown (length 23.72 mm, greatest width 8.01 mm, measurements for the right side), giving a predicted body mass of approximately 111 kg as derived from the m1 length felid regression equation of Van Valkenburgh^22^. Given the preservation, it is impossible to determine the presence of a p3.

Additionally, five cervical and three partial lumbar vertebrae are known from USNM 12820 (Fig. 1; Supplementary Fig. S2), along with portions of the left forelimb including the scapula through radius and ulna (Fig. 1, Supplementary Fig. S3). Detailed descriptions of these elements are given in the Supplementary Information.

### Nimravidae phylogeny

**Figure 4:**
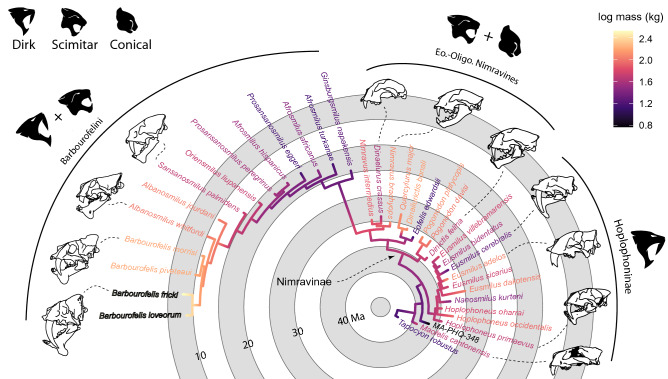
Tip-dated Bayesian phylogeny of the Nimravidae, outgroups (save *Tapocyon*) removed. Two main clades were recovered, the Hopliphoninae and Nimravinae, the latter of which includes all barbourofelin taxa. Color gradient reflects maximum-likelihood estimation of ancestral body mass. Additional nodal values (e.g., posterior probability, divergence dates) can be found in Supplementary Fig. S5.

In direct opposition to all previous nimravid phylogenetic analyses, Nimravidae was recovered with a basal bifurcation splitting all taxa (save *Maofelis cantonensis* and unnamed specimen MA-PHQ 348) into two clades, here termed Hopliphoninae and Nimravinae (Fig. 4; Supplementary Fig. S4). These clades are supported by three unambiguous synapomorphies and 97% Posterior Probability (PP) for the former, and five unambiguous synapomorphies (74% PP) for the latter. Within Hopliphoninae are found the genera *Hoplophoneus*, *Eusmilus* and *Nanosmilus*, while the remainder are within Nimravinae. Additionally, the Eocene–Oligocene Nimravinae was recovered in an arrangement that is more or less the direct opposite of prior analyses^1,4,5^, with the scimitar-tooth *Dinictis felina* as the basalmost taxon and ‘cheetah-like’^23^ *Dinaelurus crassus* as the most derived. Barbourofelini was recovered as a well-supported monophyletic clade with four unambiguous synapomorphies (93% PP) as sister to the North American and European *Nimravus* plus *Dinaelurus* clade, with three unambiguous synapomorphies (91% PP). The Barbourofelins were recovered in a primarily pectinate arrangement, leading from scimitar- to highly derived dirk-tooth taxa. Of note, the genera *Afrosmilus* and *Prosansanosmilus* were not recovered as monophyletic, but in paraphyly, a relationship previously hypothesized in literature^7,24^. Furthermore, removal of lower dentition, dentary, and postcranial (questionably referred material due to lack of associated upper dentition) characters for *Ginsburgsmilus napakensis* from the character matrix resulted in minimal change to tree topology, and primarily only a small drop in support values at major nimravine nodes, Supplementary Fig. S5.

## Discussion

All prior cladistic analyses of Nimravidae employed brief character lists that more-or-less described acquisition of sabertooth morphology. The almost six-fold increase of characters primarily to regions much, if not entirely, neglected by prior studies produced extremely disparate topologies compared to those studies. One of the major results of this analysis was the recovery of a basal split dividing nimravid evolution along two paths. One path (Hopliphoninae) leads to a plethora of early and highly derived dirk-tooth taxa, ranging from the diminutive *Eusmilus cerebralis* (~ 19 kg) to the largest, *Eusmilus adelos* (~ 111 kg), although on average members of this clade was relatively small (~ 61 kg) compared to Pleistocene sabertooth cats such as *Smilodon* and *Homotherium* (~ 150–300 kg). The other path (Nimravinae) includes the recovery of previously determined “basal” taxa (e.g., *Dinaelurus*, *Nimravus*) as derived members of their own speciose clade that converged on extant feline morphology, a hypothesis that is more congruent with the stratigraphic record. This clade/path further gave rise to some of the most derived taxa found in any sabertooth clade, such as *Barbourofelis fricki*.

The nimravids now offer a specialized take on ‘cat-like’ morphologies, tens of millions of years before their subsequent appearance in later more-familiar felids. *Eusmilus adelos* is estimated to be the largest hoplophonine, approximating the size of small individuals of the living African lion^25^. As previously stated, derived dirk-tooth nimravids of the Oligocene were typically quite small, implying a differential niche than the often compared Pleistocene sabertooth cats. This makes *E. adelos* the first described hoplophonine to approximate a size comparable to derived dirktooth cats such as *Smilodon*. Carnivores surpassing 25 kg typically hunt prey greater or equal to their own body mass^26,27^. Applying this principle to potential prey taxa, *E. adelos* would have been constrained to hunting rhinoceratids, tapirids and anthracotheriids of the Orellan (33.7–32.0 Ma^28^) American Midwest, while the majority of other hoplophonines would have preyed upon the diversity of ‘oreodont’, equid and camelid taxa^29^. This niche partitioning is typical of African felid ecosystems today, with lions frequently consuming large bovids and equids, while leopards prey on a diversity of large rodents, small bovids and even small canids^25^.

Eocene through Oligocene nimravines include both the smallest and largest known nimravids during this time period, featuring both scimitar- and conical-tooth morphology. *Quercylurus* and *Dinailurictis* attained African lion proportions (> 140 kg), but additionally possessed unusual, highly crenulated P4s. Little is known of the non-dental morphology of these giant nimravines, but they would have been some of the largest carnivores of the Oligocene in Europe. However, compared to the large (79–142 kg) majority of early nimravines, *Eofelis* is the smallest known nimravid (~ 14.5 kg) of this time period, comparable in body size to the living caracal. The inferred diverse paleoecology of these scimitar-tooth taxa paints a picture remarkably reminiscent of the Pleistocene, with homotherin and smilodontin sabertooth felid taxa co-existing.

*Dinaelurus* is unique amongst nimravids in its conical canines lacking serrations, its domed cranium, and its enlarged internal nares. These features have been suggested to be similar to a ‘cheetah-like’ morphology, though post-crania are still unknown for this taxon. The phylogeny of this study supports these morphological features as derived (and thus possible adaptations), compared to previous results suggesting that it retained an ancestral morphology relative to sabertooth taxa. Compared to the evolutionary history of felids, a startingly convergent trajectory of evolution is implied by these results. Felids are derived from ancestors possessing compressed canine teeth, and only geologically recently acquired the familiar conical-tooth condition^30^. Likewise, *Dinaelurus* evolved from sabertooth ancestors within the Nimravidae, but can now be viewed as exploring the conical-tooth ecomorphology approximately 18 million years earlier.

The origin of the barbourofelins is now well constrained to be within Nimravinae, sister to the European and North American taxa *Nimravus* and *Dinaelurus*. Similar to Barrett et al.^2^ the recovered phylogeny implies a migration of nimravine taxa into Africa at MN2, which gave rise to the earliest barbourofelins. These relatively small scimitar-toothed taxa (~ 14–29 kg) were some of the first carnivorans in Africa^2,31^, entering an ecosystem dominated by a diversity of carnivorous hyaenodonts^32,33^. Hyaenodonts may have constrained, via competition, these early barbourofelins to their relatively small sizes, yet barbourofelins may have maintained a niche with their derived dental morphology. Barbourofelins subsequently spread to Eurasia and eventually North America, by which time they had greatly increased in size (~ 103–328 kg) and their degree of sabertooth morphology. The last, and most derived, nimravids were extinct by 7.0 Ma^34^, though probably not due to competition with North American felids given the limited temporal overlap between these two families^35^. Instead, nimravids more likely met their end due to general faunal turnover at the end of Hh2 that also saw a major reduction in diversity of numerous prey taxa, such as equids, camelids, antilocaprids and dromomerycids^36^.

Nimravids can be viewed as occupying three distinct morphological *baupläne*: dirk-tooth, scimitar-tooth and conical-tooth. These morphologies are now recovered as derived evolutionary pathways, not simply steps on the way to some ultimate sabertooth carnivoran. Ecologically, nimravids are typically cast in the mold of ‘cat-like’, most notably with the colloquial moniker of ‘false sabertooth cat’. Indeed, most known nimravids possessed compressed canines with serrations and the associated cranial adaptations to wield such hardware. Much literature has been devoted to these sabertooth comparisons, citing iterative evolution, but the presented findings raise the question, what are the advantages of conical-tooth morphology in ‘cat-like’ organisms given its iterative nature? However, due to the relatively great phylogenetic distance between nimravids and felids, and the range of morphologies nimravids achieved some tens of millions of years before their appearance in felids, maybe referring to cats as ‘nimravid-like’ would be most appropriate.

## Methods

### Phylogeny

The phylogenetic position of *E. adelos* was assessed via tip-dated Bayesian phylogenetic analysis in Beast2 v. 2.6.3^37^. All well-represented Eocene-Miocene nimravid taxa (34 taxa), the new taxon and four outgroups were coded for 225 morphological characters, some of which are autapomorphic (Supplementary Information) from direct observations of specimens, or from literature as listed in Supplementary Table 1. Tip dates were sampled from a uniform distribution of known first to last appearances, following the stratigraphic data in the electronic supplementary material. Ten competing schemes of character partitioning and evolution were assessed by Bayes Factor via a Generalized Stepping Stone (GSS)^38^ analysis (Table 1). These schemes partitioned characters based upon anatomical association (hypothesized sabertooth integrated morphology^10,39,40^ or cranial/post-cranial), number of states a character contains (n-states), or a combination of the two. I additionally wrote custom evolutionary rate matrices for specific multistate characters (Supplementary Information) that included ordered, multipath and Dollo (irreversible) characters to compare to a null unordered character evolution. Each scheme was assessed by GSS with 10 steps, a chain length of five million and 50% burnin. All characters were set to evolve under the Lewis Mkv model to account for ascertainment bias in the character matrix. Substitution rates were further set to vary across characters according to a single shared Gamma distribution, while a single morphological clock was modeled with an uncorrelated relaxed clock with log-normal distributed rates, a position advocated in the integration and modularity literature e.g. Ref.^41^. A fossilized birth–death (FBD) model with sampled ancestors^42–44^ was employed as a prior for the distribution of time trees.

**Table 1:** Summary of marginal log-likelihoods and Bayes factor (BF) support for differential substitution models and partitioning schemes of the morphology data. Log BF values are reported as the support of the best model (model 10) over the inline model. *P* partition used. ^a^Best supported model.

#	n-state partition	Cranial/post-cranial part	Sabertooth anat. part	Complex Evo. models	Marginal log-likelihood	Log BF vs best model
1			P		− 3399.72	851.29
2		P			− 3390.56	842.13
3		P		P	− 3070.96	522.53
4			P	P	− 2999.51	451.08
5	P		P		− 2607.97	59.54
6	P	P			− 2602.99	54.56
7	P				− 2599.59	51.16
8	P	P		P	− 2554.79	6.36
9	P		P	P	− 2550.78	2.35
10^a^	P			P	− 2548.43	–

Turnover rate, diversification rate and fossil sampling proportion were estimated, but given initial values as derived from the carnivoran diversification analysis of Liow and Finarelli^45^, where turnover (r) = μ/λ = 0.5, net diversification (d) = λ − μ = 0.05, and sampling proportion (s) = ψ/(μ + ψ) = 0.5. Furthermore, turnover was given a uniform distribution prior, while diversification an exponential with a mean of 1.0 and sampling proportion a beta prior (2.0, 2.0). The Rho parameter was conditioned with a value of 0.0083, being the fraction of living carnivorans in the analysis (1/286)^46^. The origin parameter was estimated at 49.75 Mya^2^, with a lognormal distribution, offset of 45.15 Mya (being the FAD of the oldest taxon in the analysis), mean of 1.0, standard deviation of 1.25 and ‘mean in real space’ parameter selected. A single topology prior was employed that enforced monophyly of Nimravidae to the exclusion of the outgroup taxa. The XML file for this analysis (and other partitioning schemes) containing all the employed parameters is available in the electronic Supplementary Dataset S1.

Markov Chain Monte Carlo (MCMC) runs were sampled every 1000 generations, while trees were logged every 10,000 generations until Effective Sample Sizes (ESSs) were > 200, as determined in Tracer version 1.7.1^47^. From the output of Tracer, sampling and timing (generation number) of the stability of the run was assessed. Thus, the first 10% of the generations were discarded (burn-in), and topology and posterior probabilities were estimated from the remaining generations on a maximum clade credibility (MCC) tree with median node height estimates (Supplementary Fig. S4). An additional analysis was run using the best-supported settings of the previous analyses to test the impact of referred dentary and postcranial *Ginsburgsmilus napakensis* material^48^. There are no specimens with associated upper and lower dentition for this species, creating doubt for referral of this material to other stem Afrosmilini taxa, e.g., *Afrosmilus turkanae*. Thus, a second analysis was run that did not include lower dentition, dentary or postcranial material for *Ginsburgsmilus napakensis*.

### Ancestral character state estimation

Synapomorphies were assessed via ancestral character state estimation (ACSE) in BayesTraits v. 3.02^49^. ACSE was used to identify the likely plesiomorphic condition for analyzed characters at the base of Nimravidae, and to identify shared, derived character states (synapomorphies) within the clade. Possible synapomorphies were identified by using the method of Paterson et al.^50^, first by analyzing the entire morphology character matrix on the MCC tree in TNT v. 1.5^51^. From this list of both ambiguous and unambiguous synapomorphies (51 in total), I assessed their state likelihoods in BayesTraits. To account for phylogenetic uncertainty, ancestral character state estimates were calculated using a random sample of 1000 post-burnin trees from the stationary pool of the Beast2 analysis described above. I used the AddNode option for the reconstruction of seven internal nodes of the full phylogeny: Nimravidae, Hopliphoninae, Nimravinae, *Eusmilus*, the Barbourofelini plus *Nimravus* and *Dinaelurus* clade, Barbourofelini, and the *Albanosmilus* plus *Barbourofelis* clade. I chose the Multistate method and MCMC parameters of five million iterations, sample period of 1000 and a burnin of ten percent. I used the Reversible-Jump MCMC method with an exponential hyperprior of 10 to assess best fitting models without necessitating choice in priors devoid of a preexisting empirical framework. MCMC sampling and ESS values (sufficient sampling considered ESS > 200) were assessed in Tracer v. 1.7.1, while the estimated ancestral states were evaluated, and mean probabilities plotted at each node with the aid of R packages ‘btw’ v. 2.0^52^ and ‘phytools’ v. 0.7-47^53^. The associated run files, mapped character states and list of synapomorphies for all fifty-one characters can be seen in Supplementary Dataset S2.

### Body mass estimation

Body mass, as depicted in Fig. 4, was estimated via the m1 length felid regression equation of Van Valkenburgh^22^. These estimates were derived from mean values of m1 length from specimens and literature listed in Supplementary Table 1. Not all taxa have known lower dentition and thus *Maofelis cantonensis*, MA-PHQ-348 and *Dinaelurus crassus* were estimated from the felid regression equation of skull length from Van Valkenburgh^22^. The remaining taxa were estimated/imputed using the ‘fastANC’ function found in ‘phytools’^53^.

## Supplementary Information

Supplementary Information 1.

Supplementary Information 2.

Supplementary Information 3.
