# Supplementary material for: The largest hoplophonine and a complex new hypothesis of nimravid evolution
Source: Sci Rep. 2021 Oct 26;11:21078. doi: 10.1038/s41598-021-00521-1 (PMC8548586; doi:10.1038/s41598-021-00521-1)

Synapomorphy 7

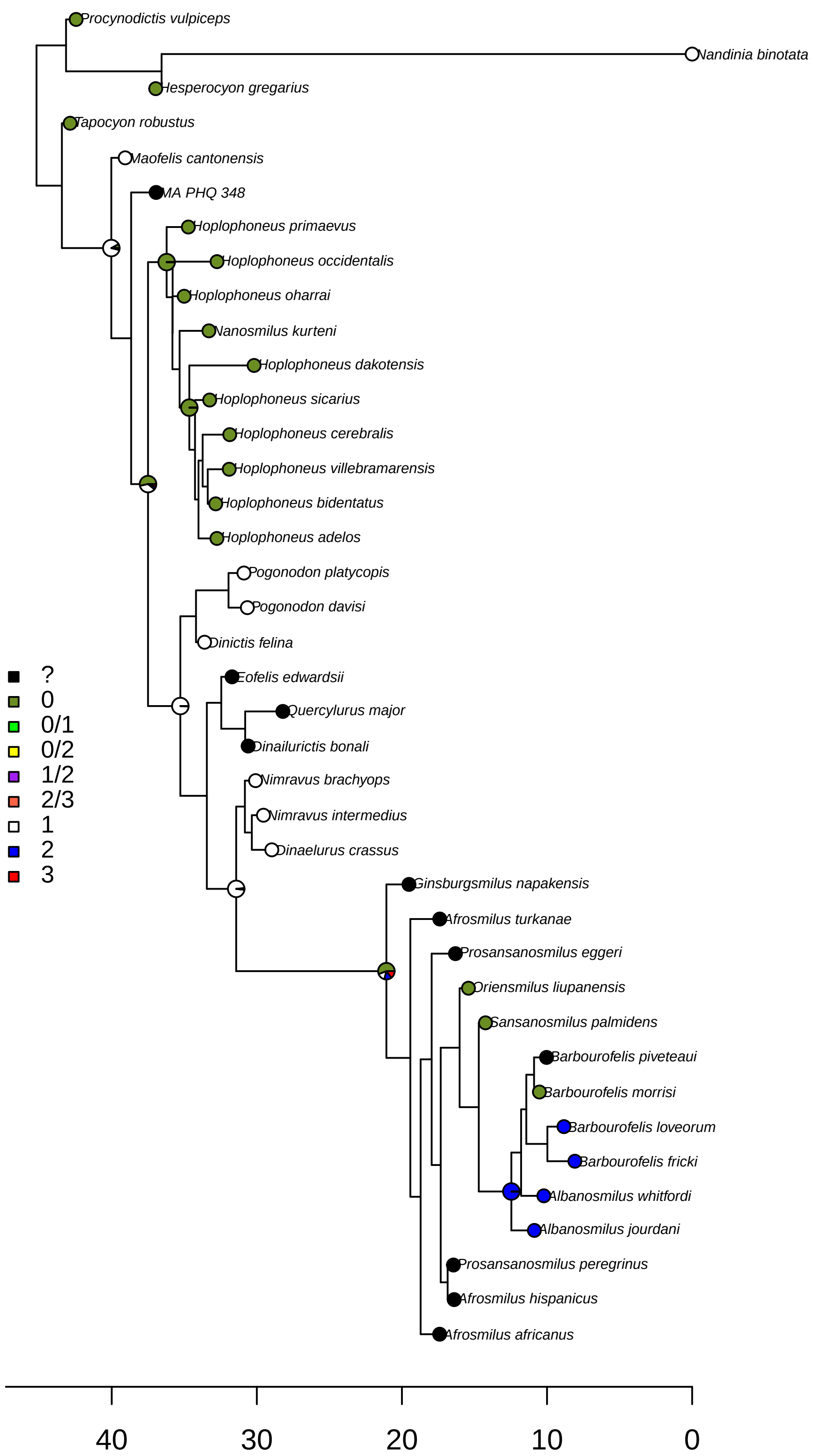

Synapomorphy 8

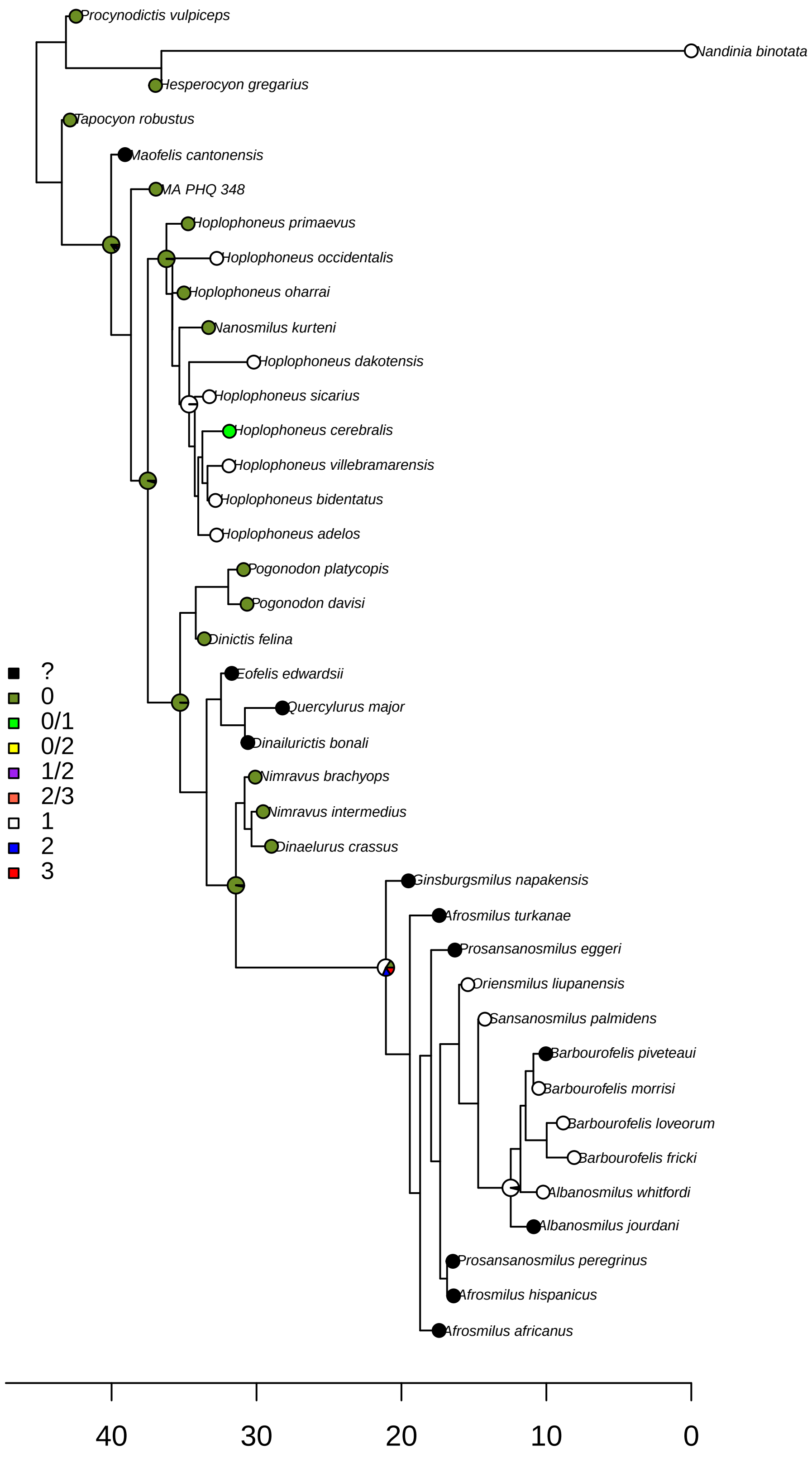

Synapomorphy 9

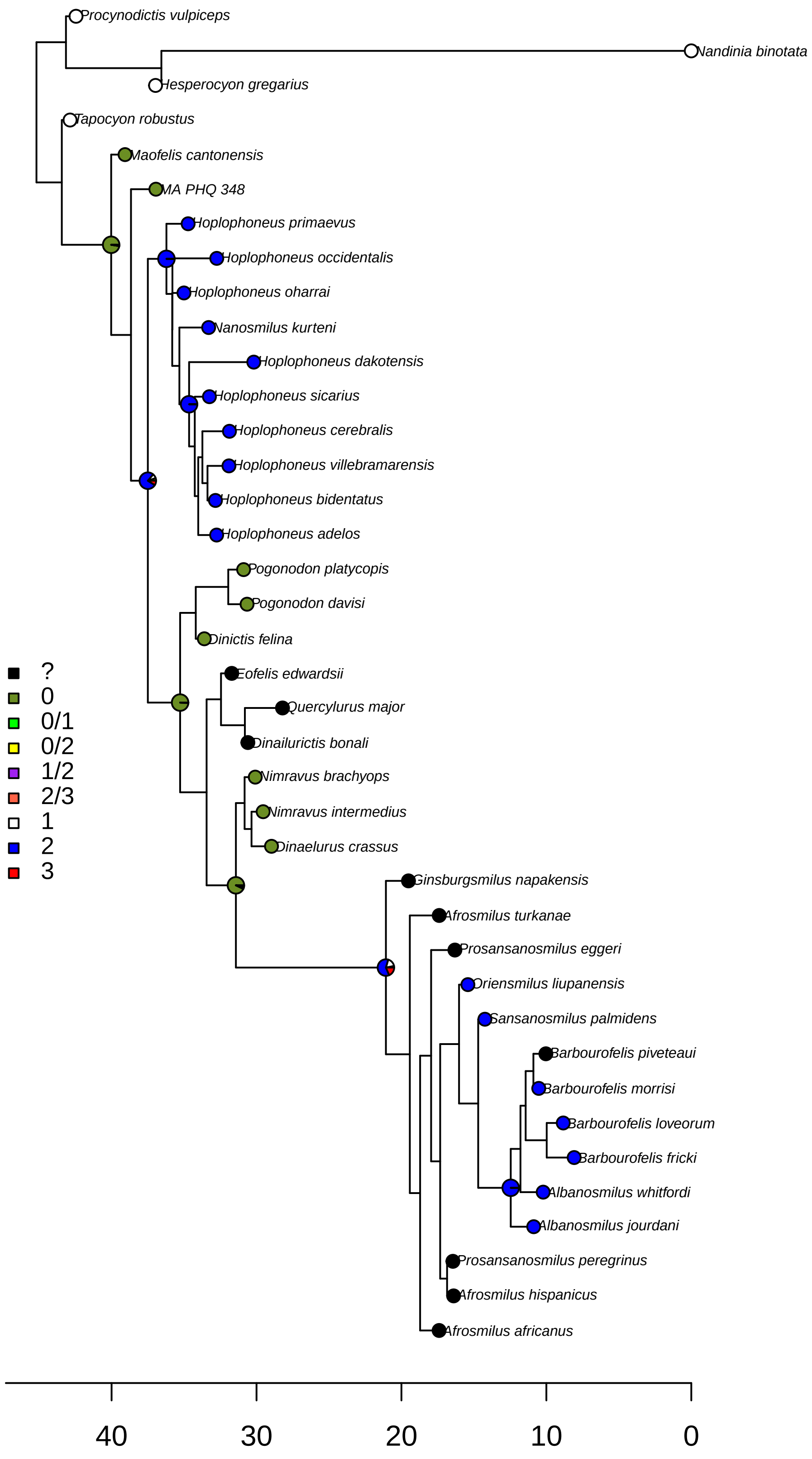

Synapomorphy 11

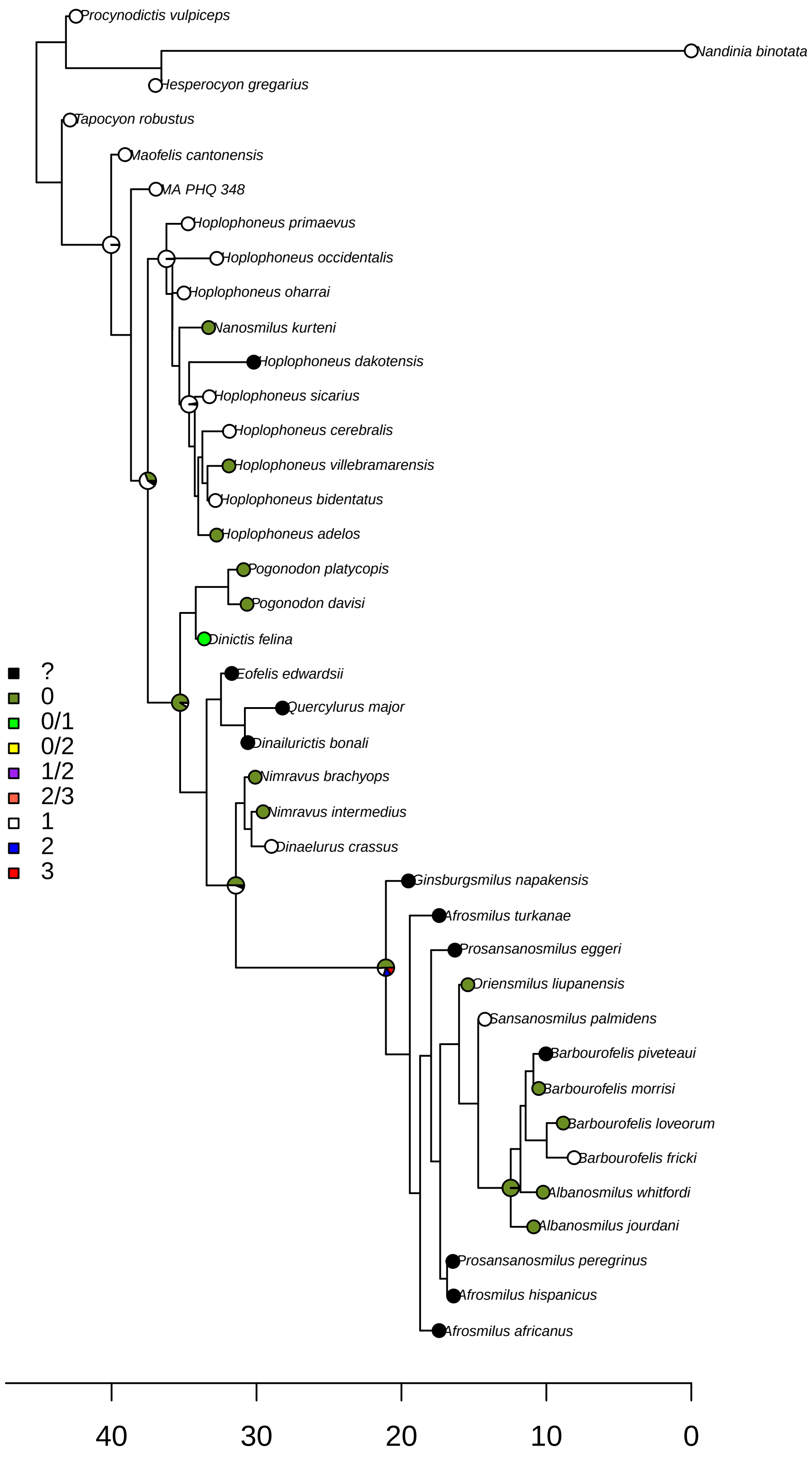

Synapomorphy 15

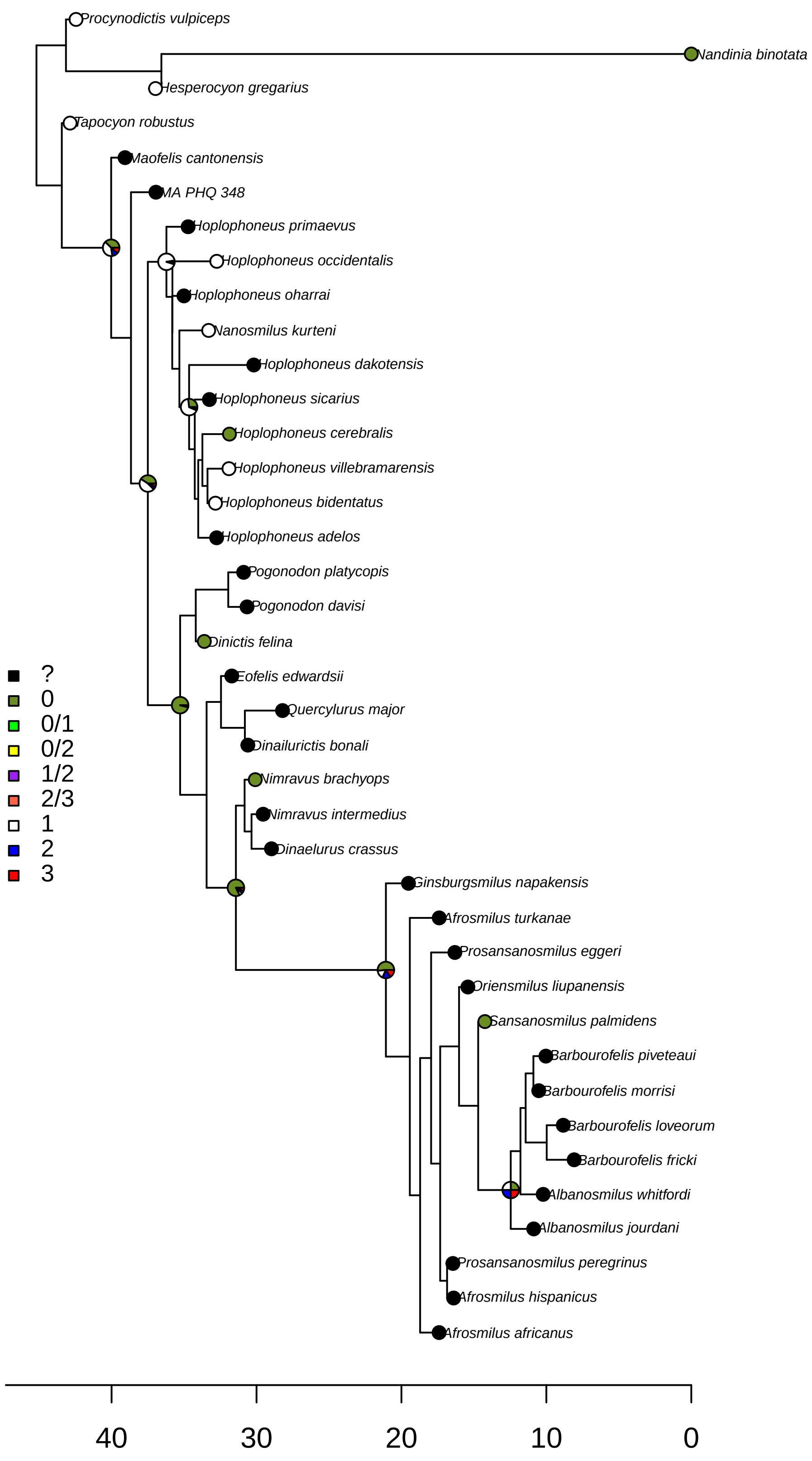

Synapomorphy 20

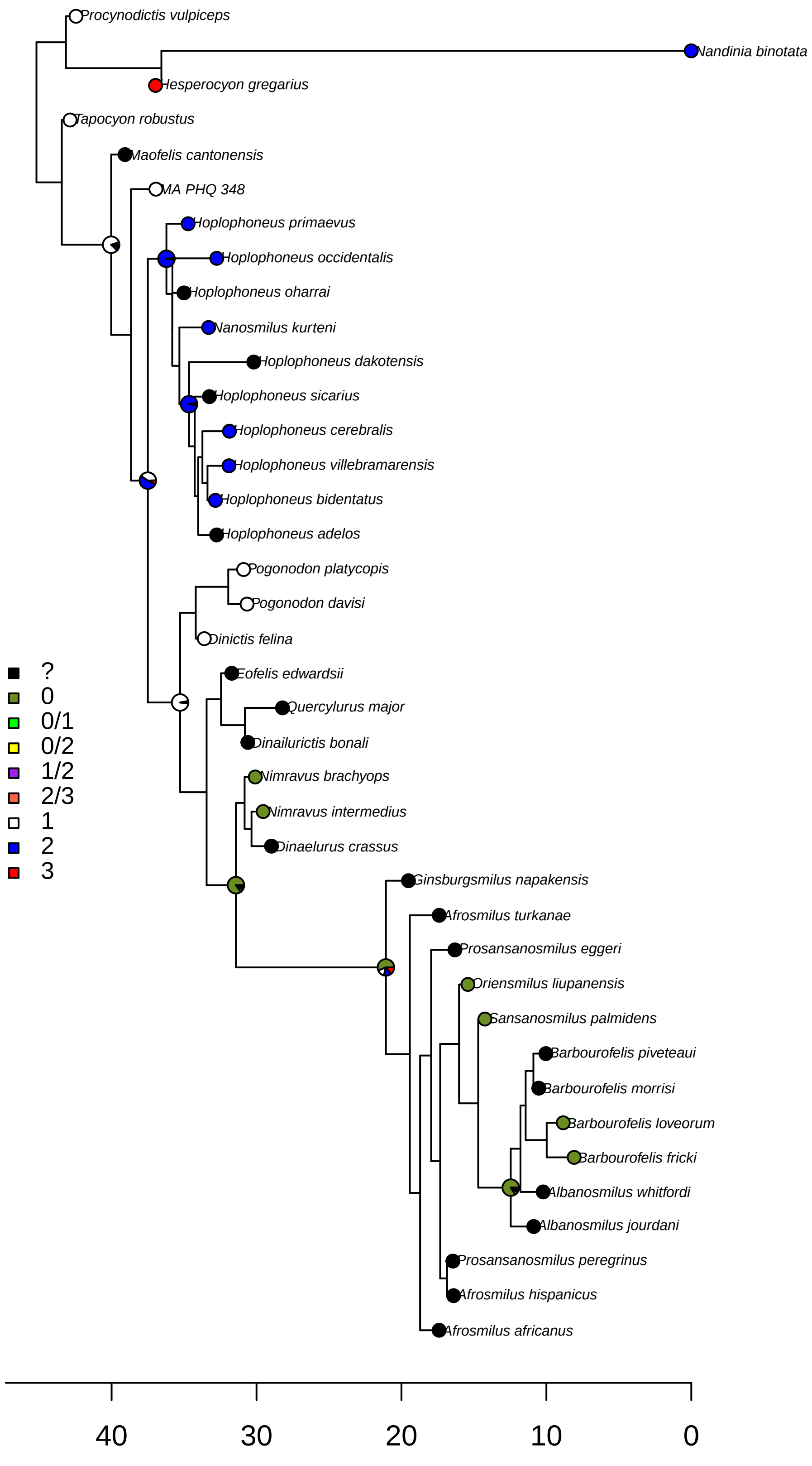

Synapomorphy 29

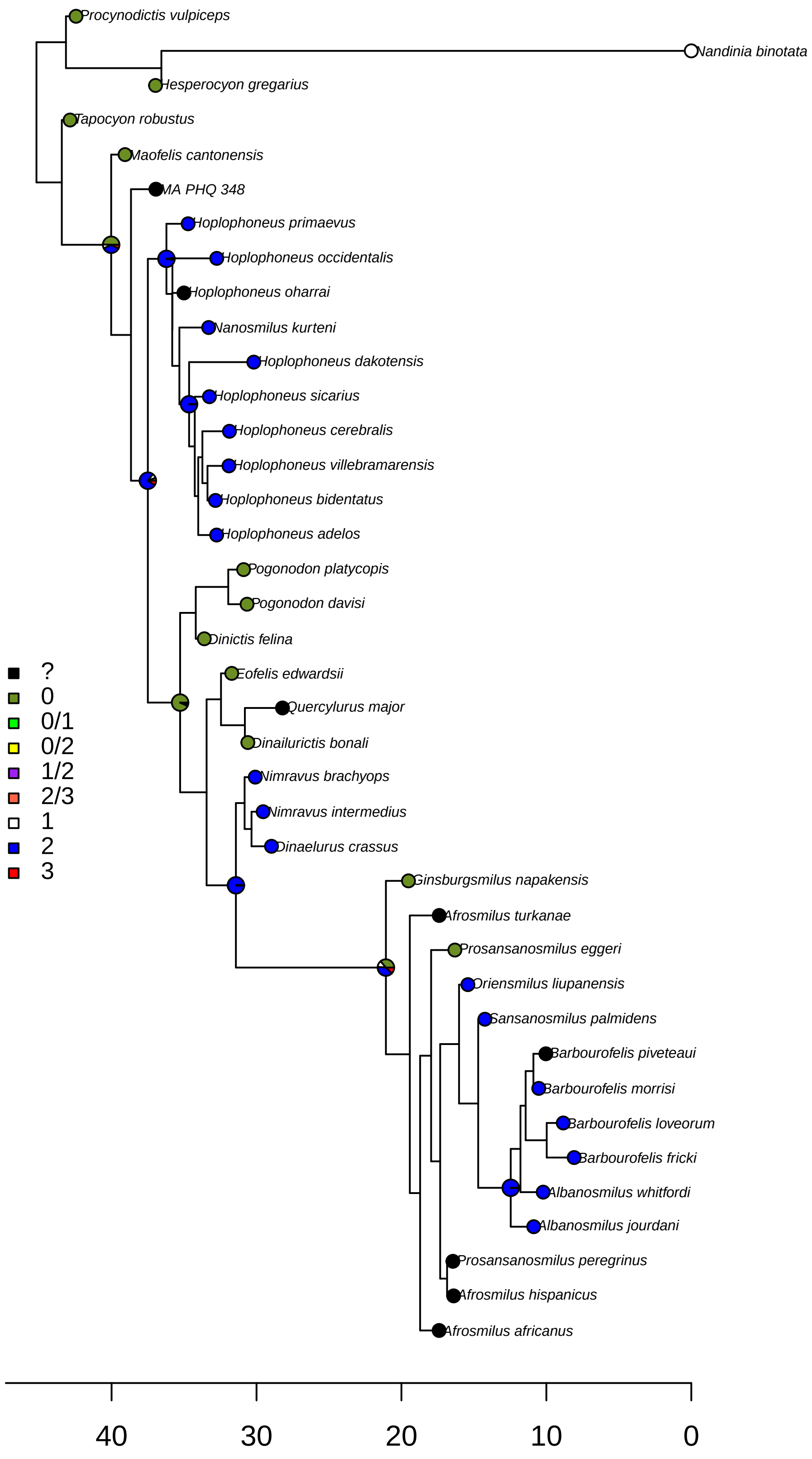

Synapomorphy 33

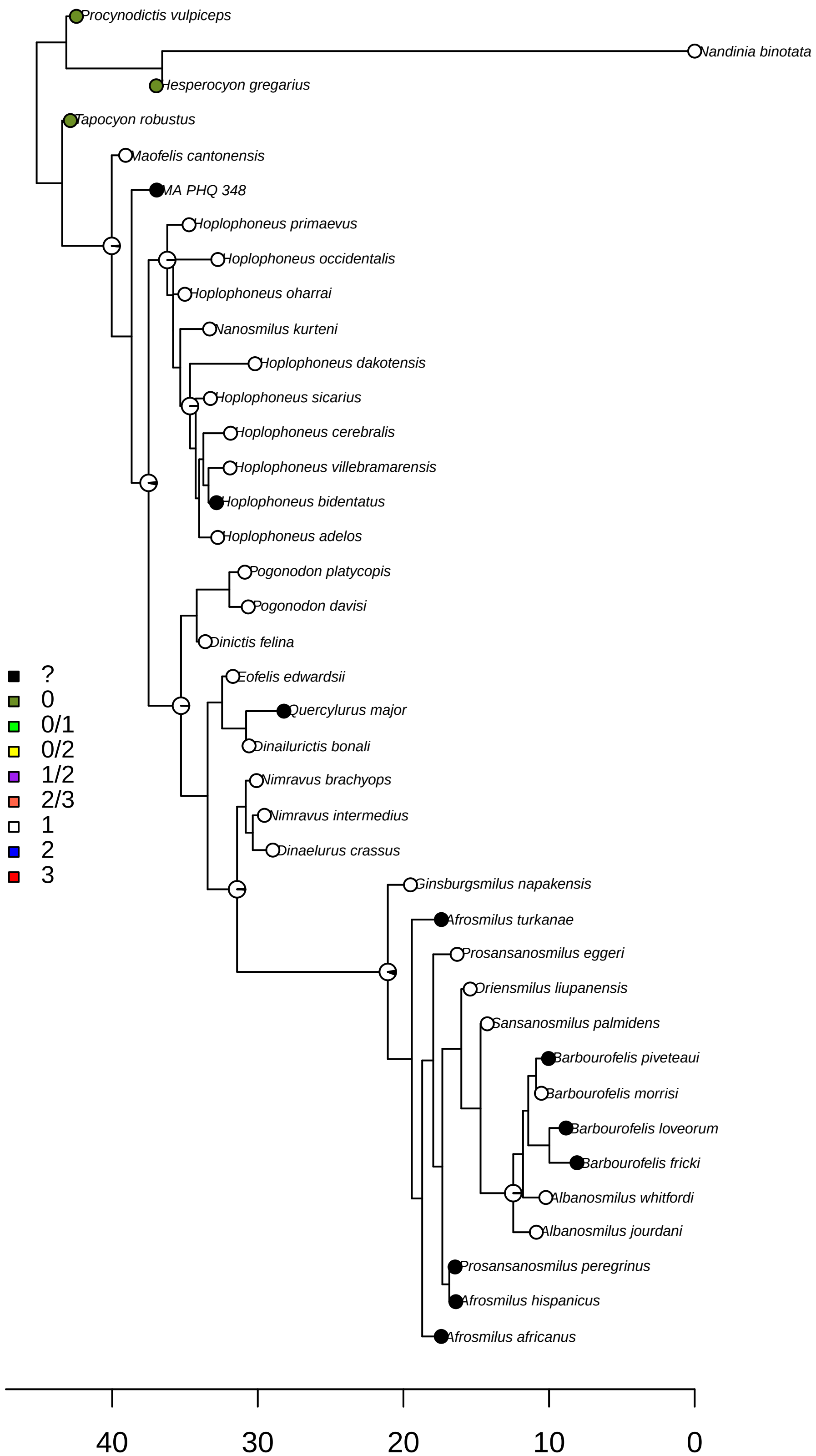

Synapomorphy 36

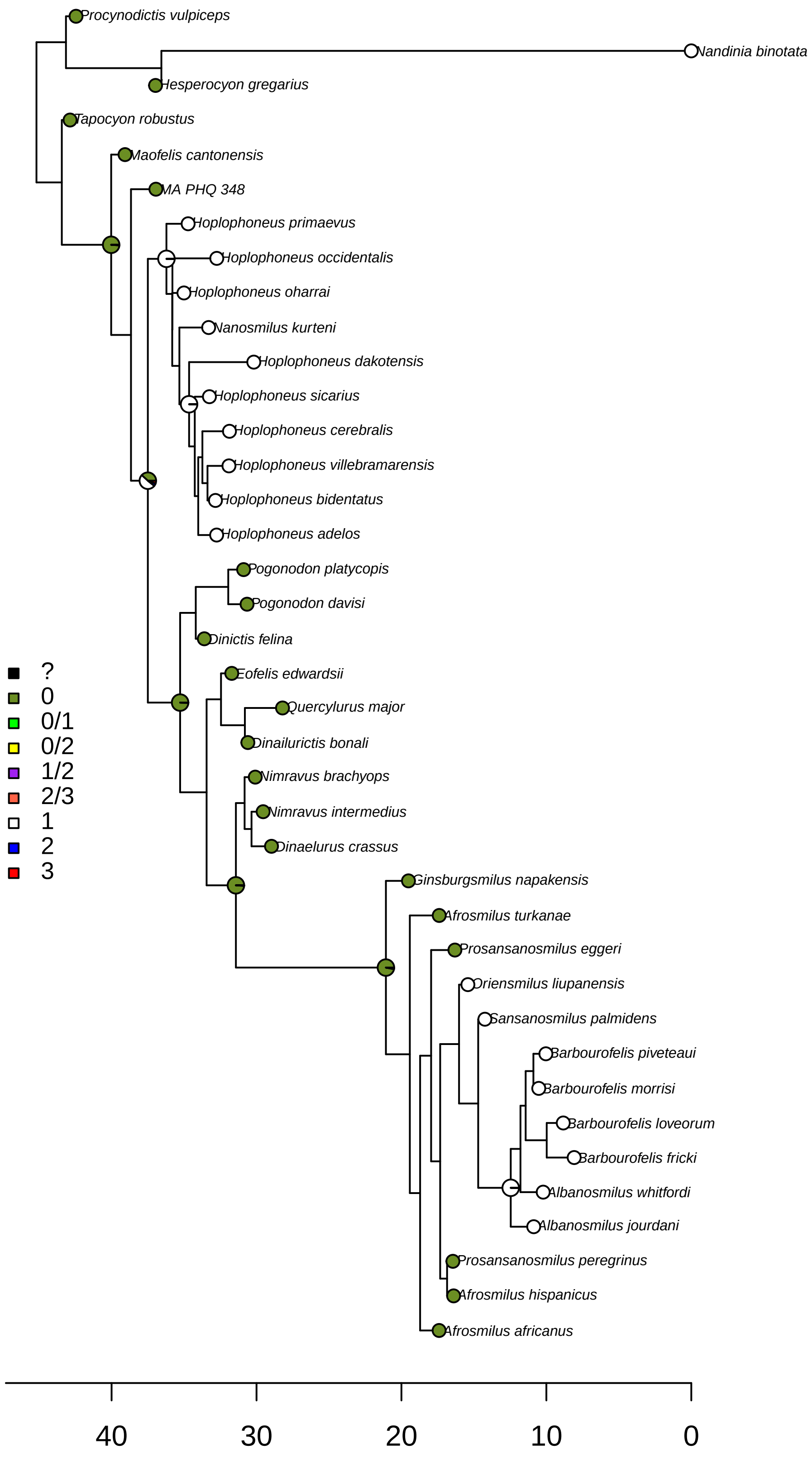

Synapomorphy 37

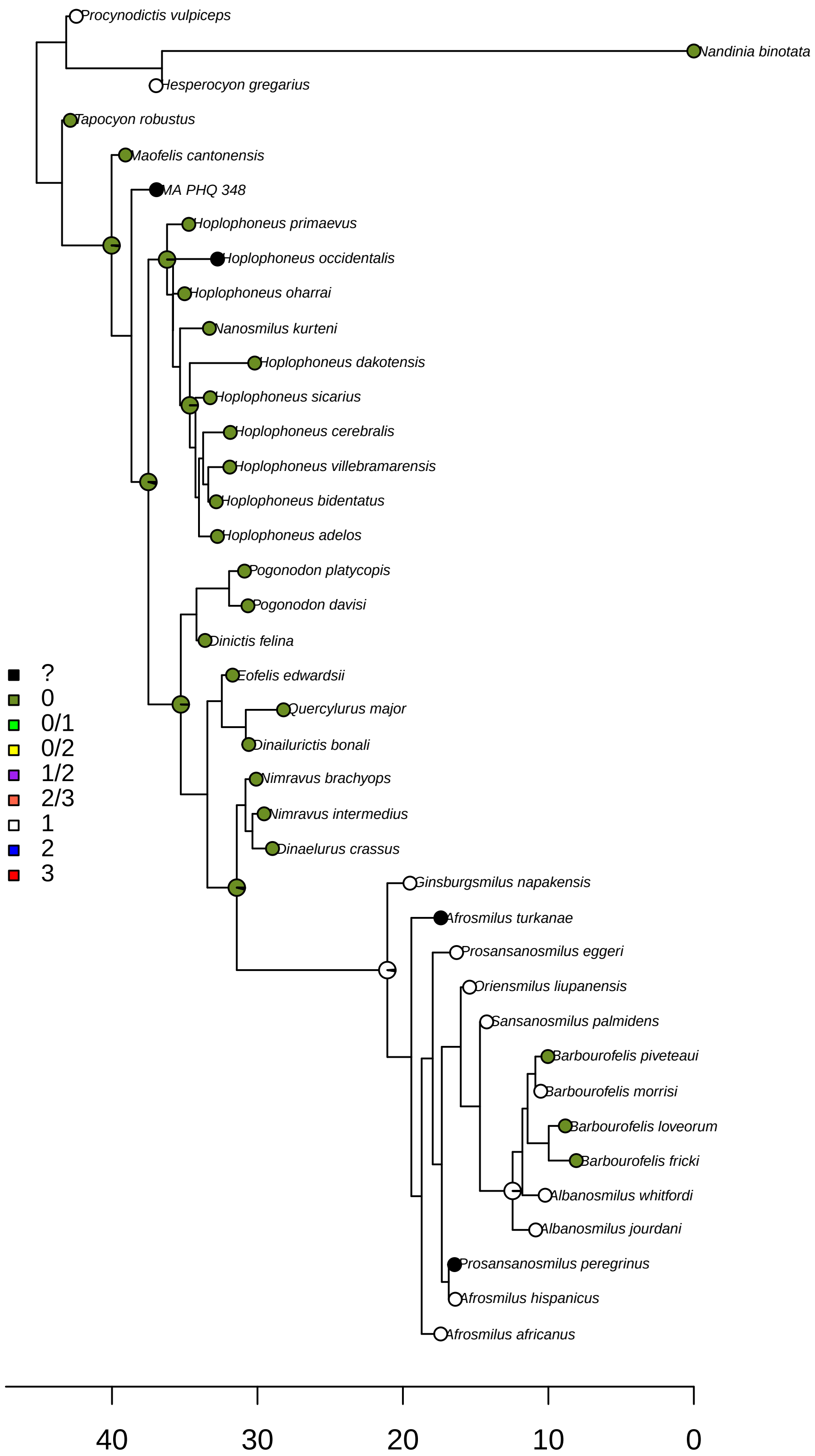

Synapomorphy 44

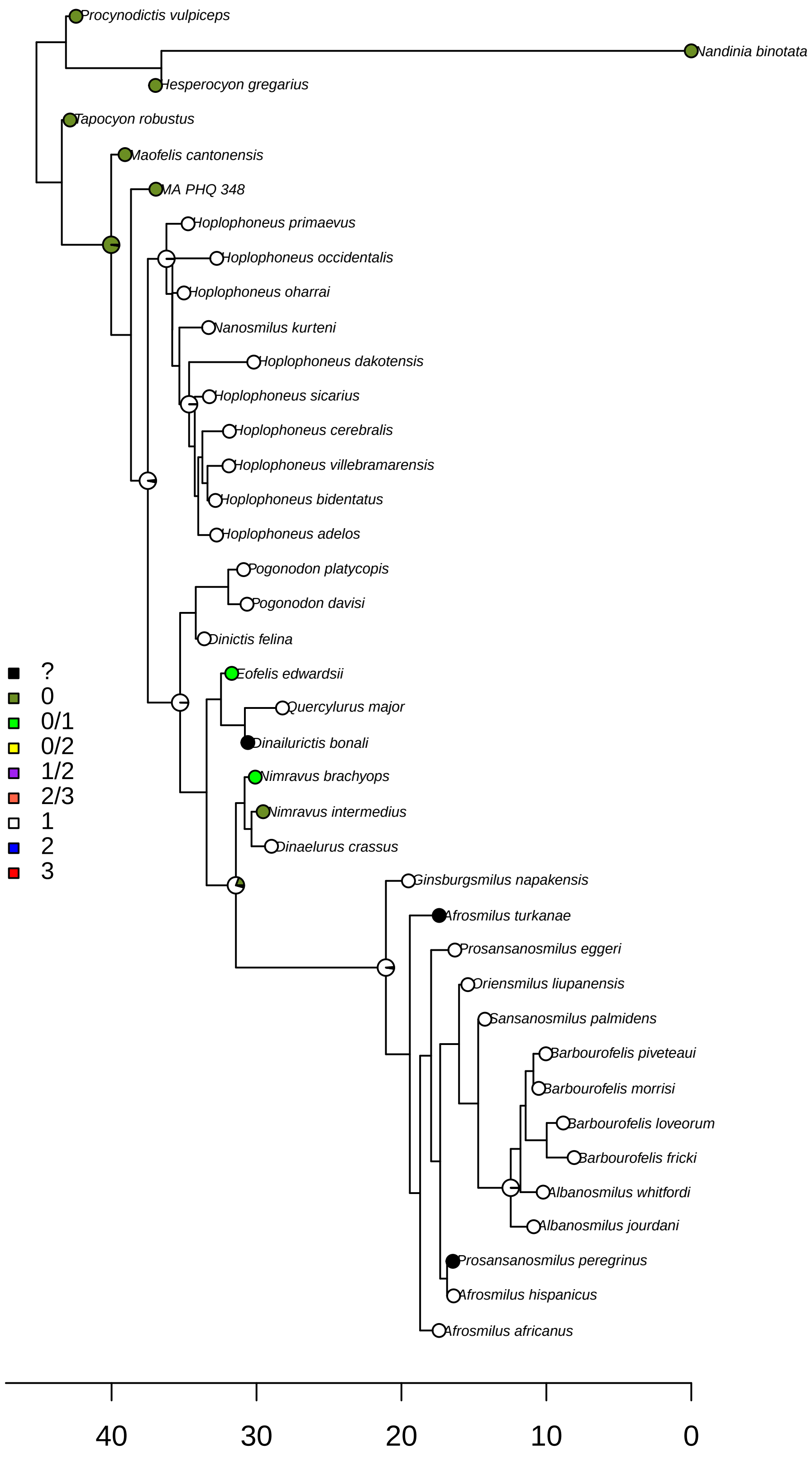

Synapomorphy 83

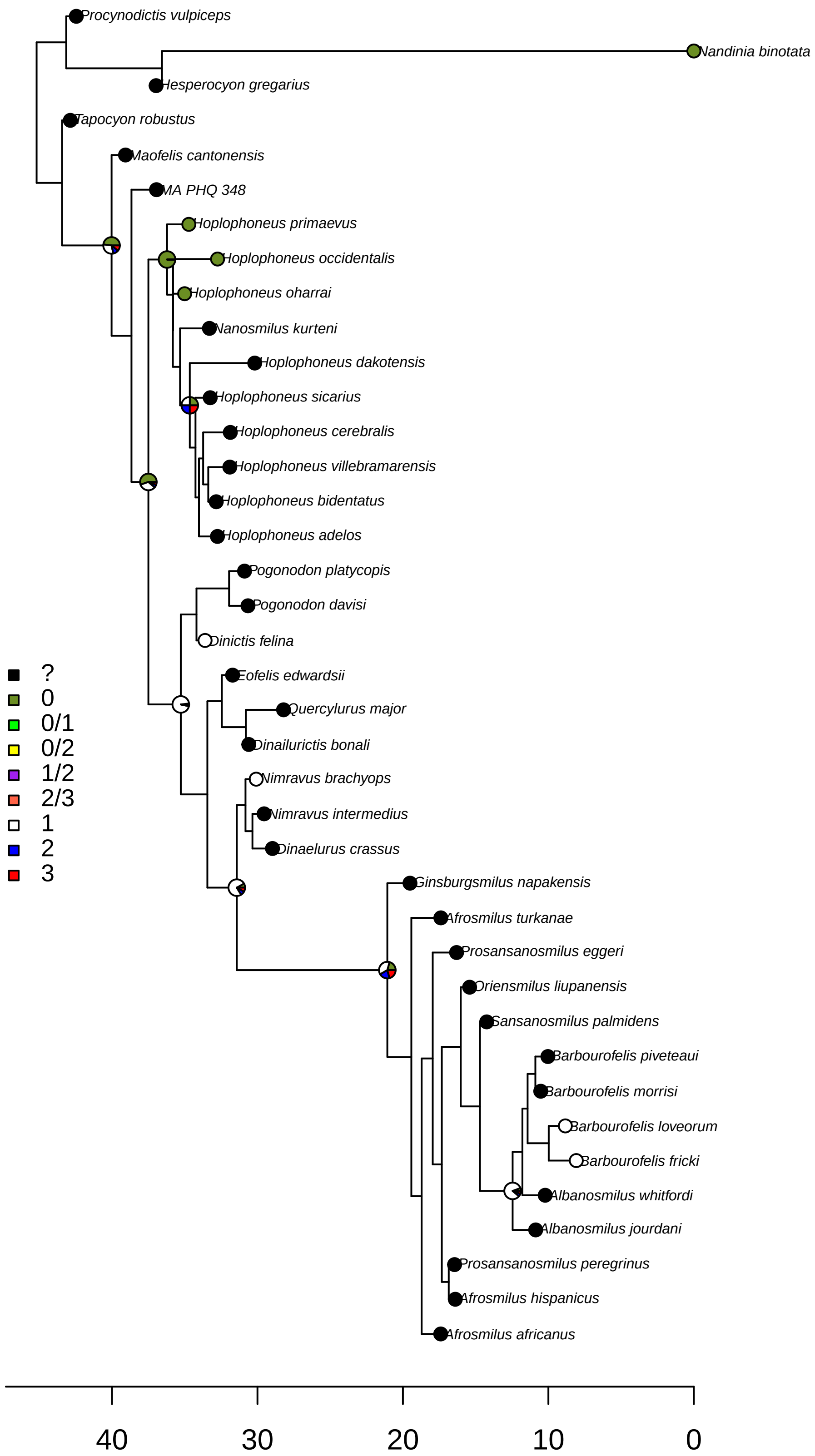

Synapomorphy 85

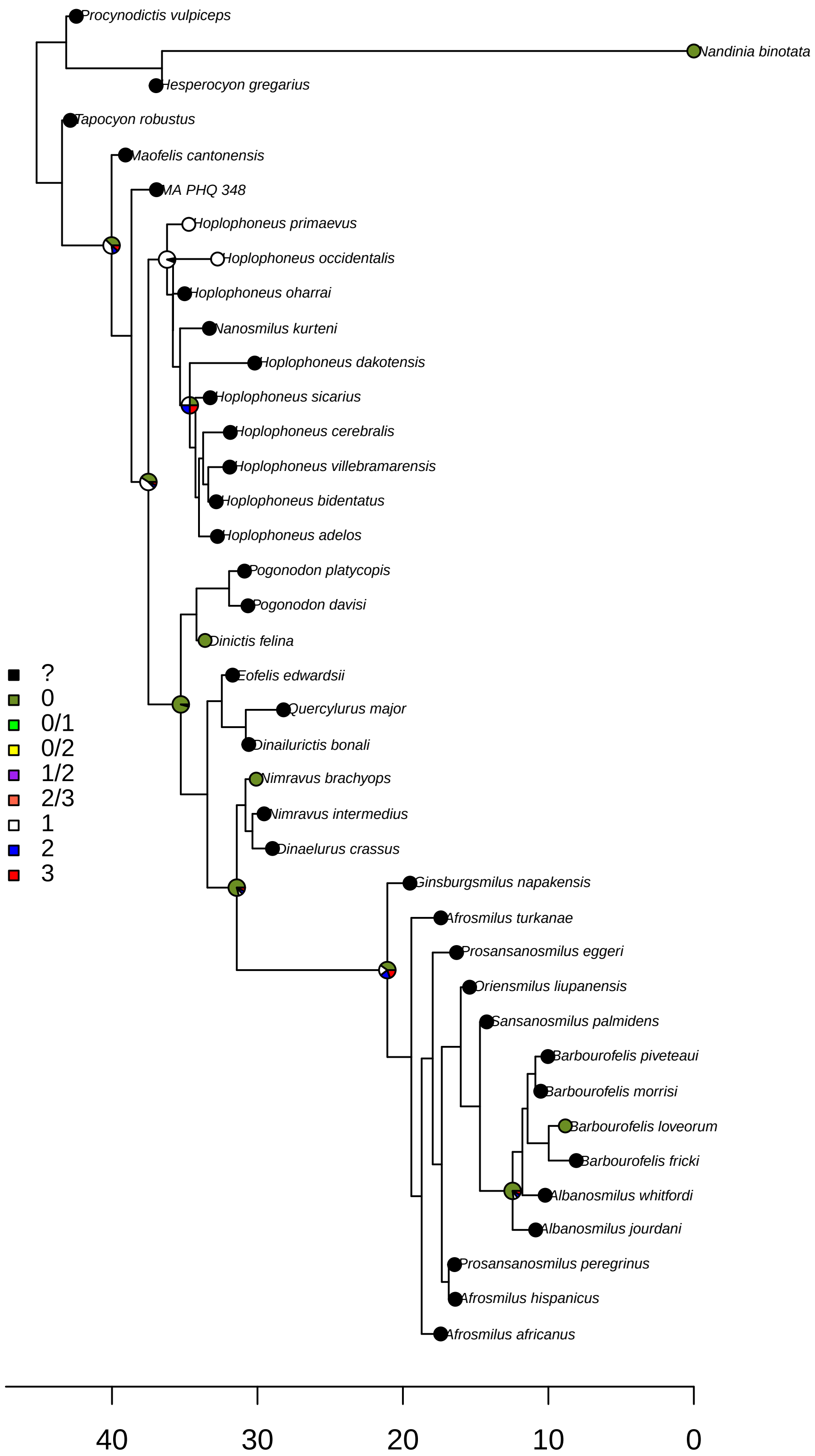

Synapomorphy 96

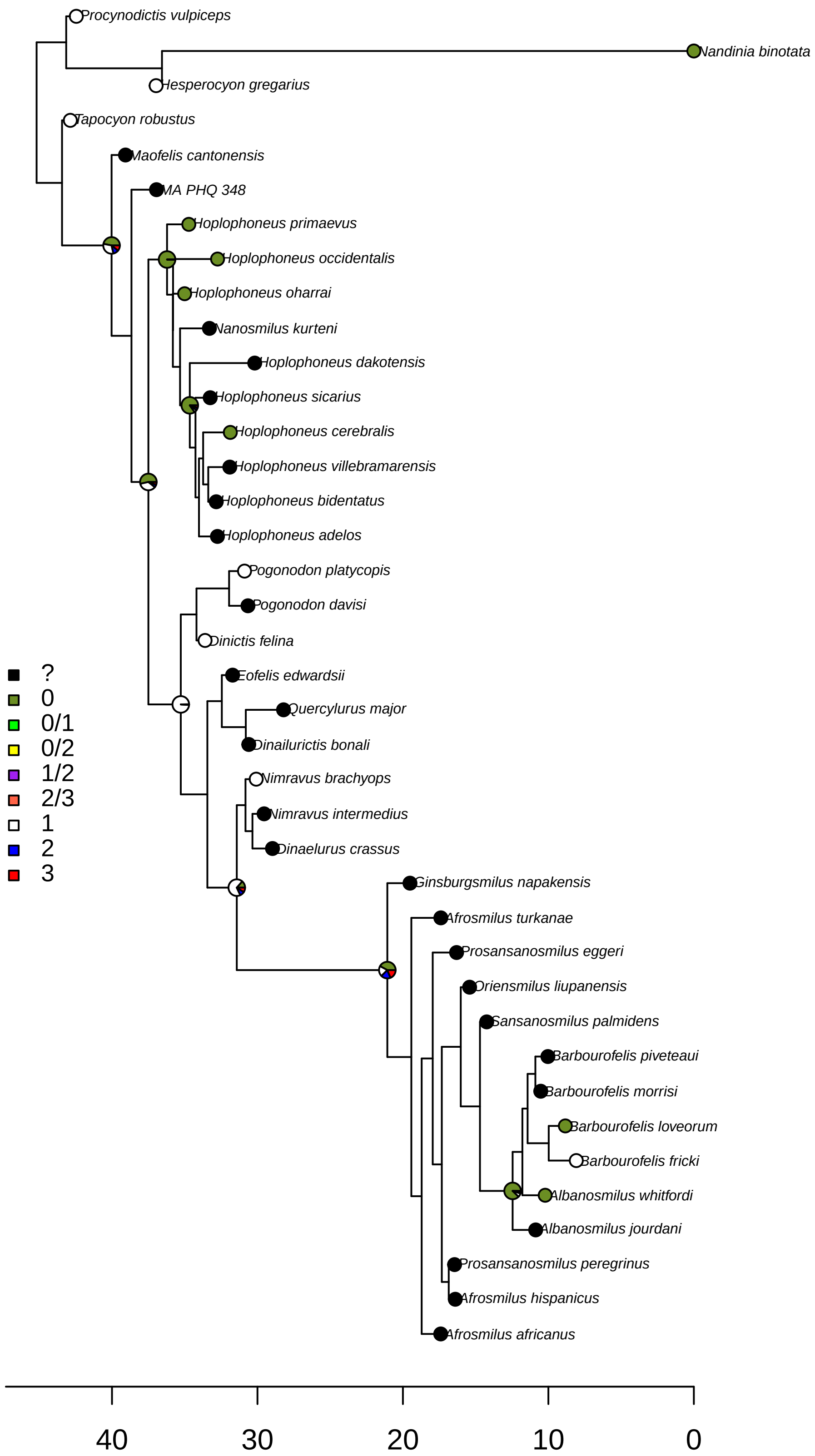

Synapomorphy 103

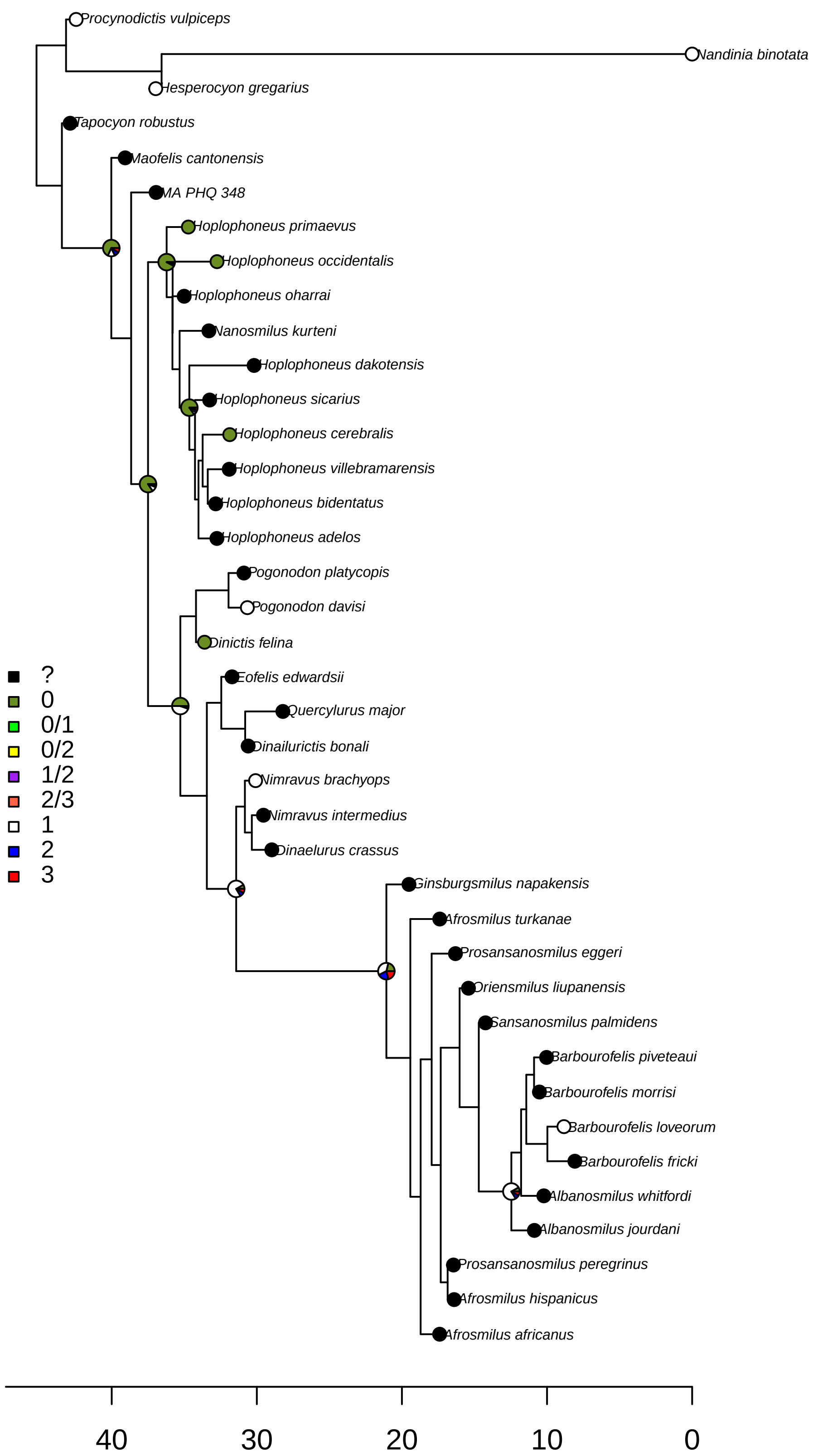

Synapomorphy 116

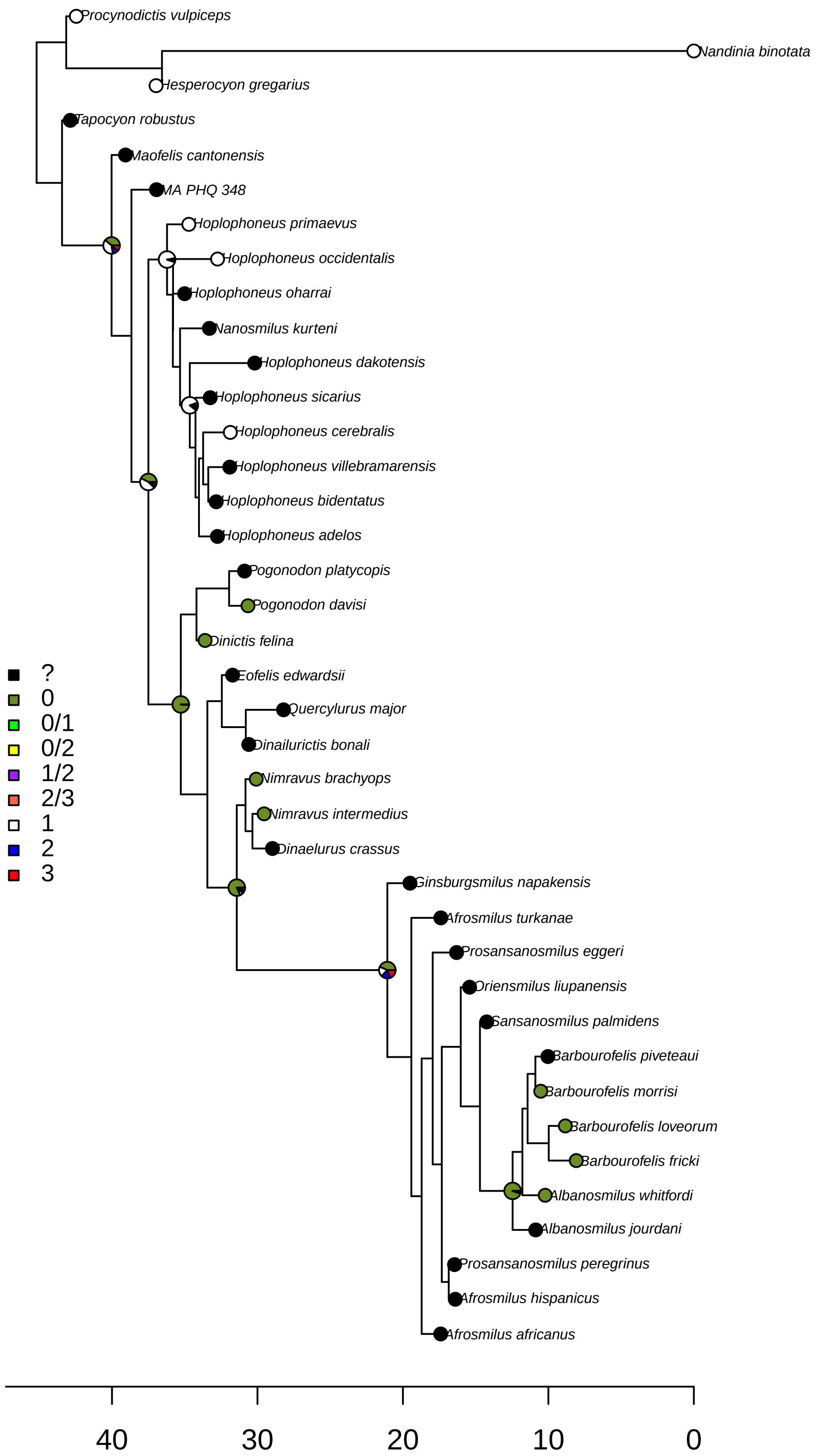

Synapomorphy 133

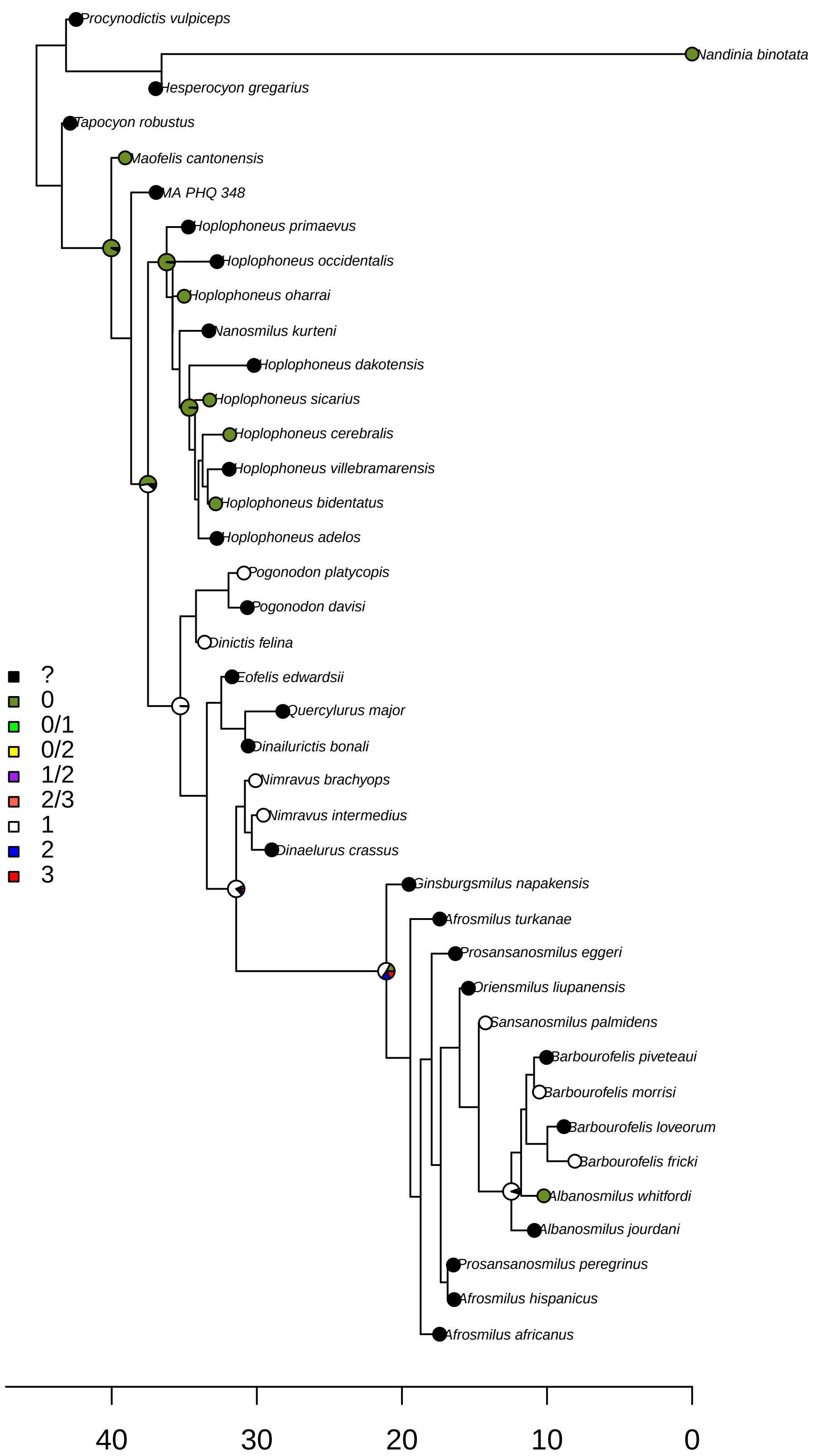

Synapomorphy 135

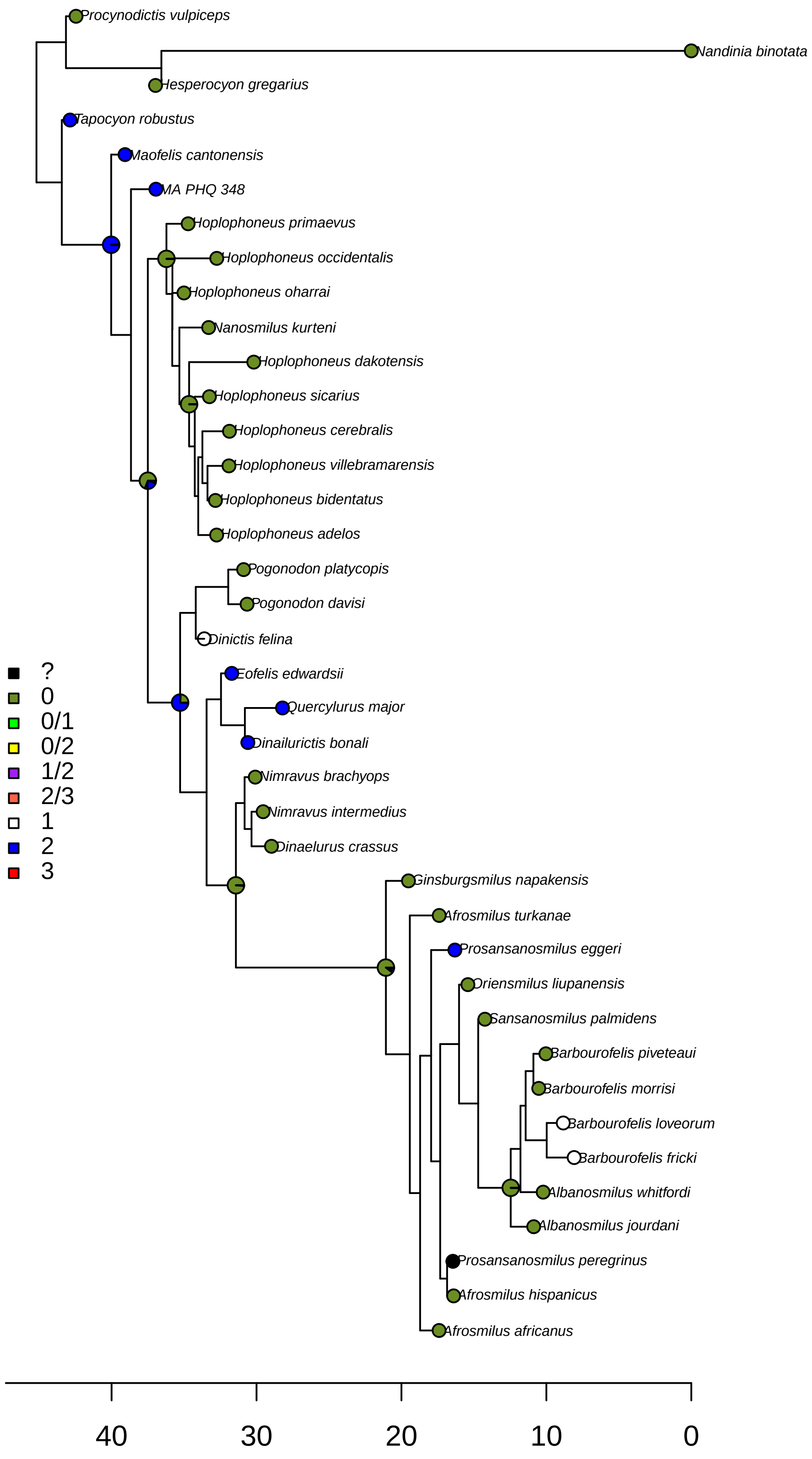

Synapomorphy 136

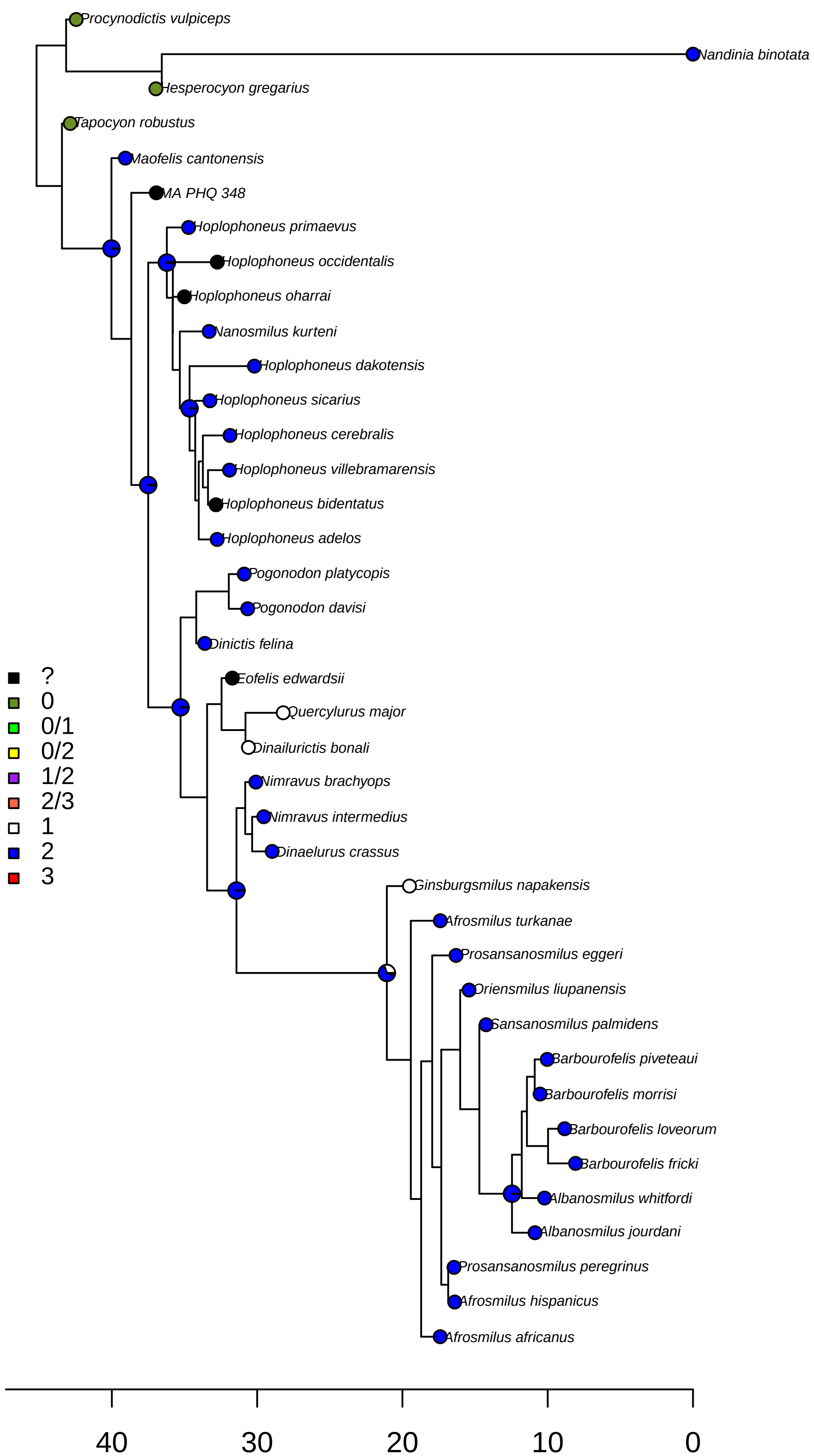

Synapomorphy 138

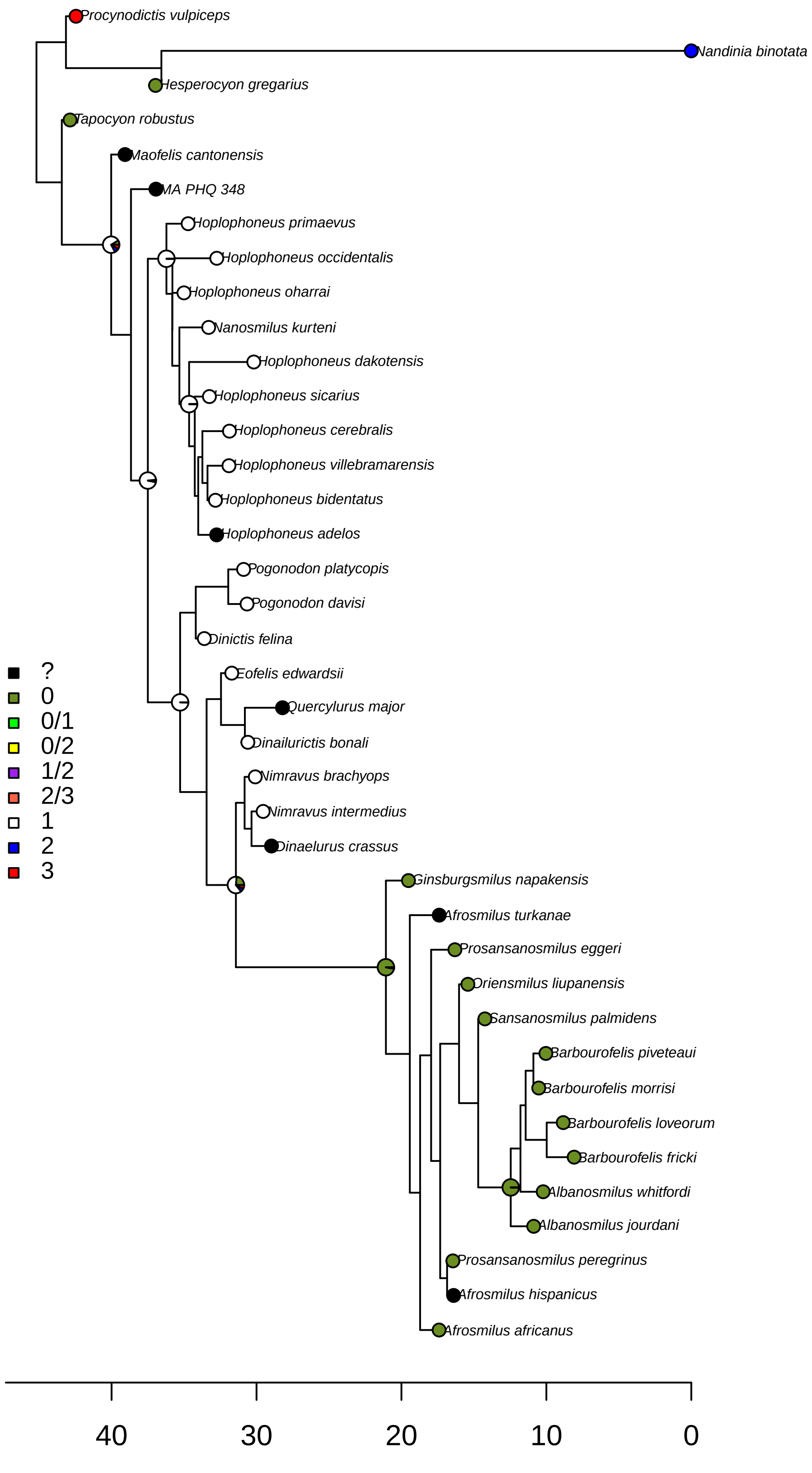

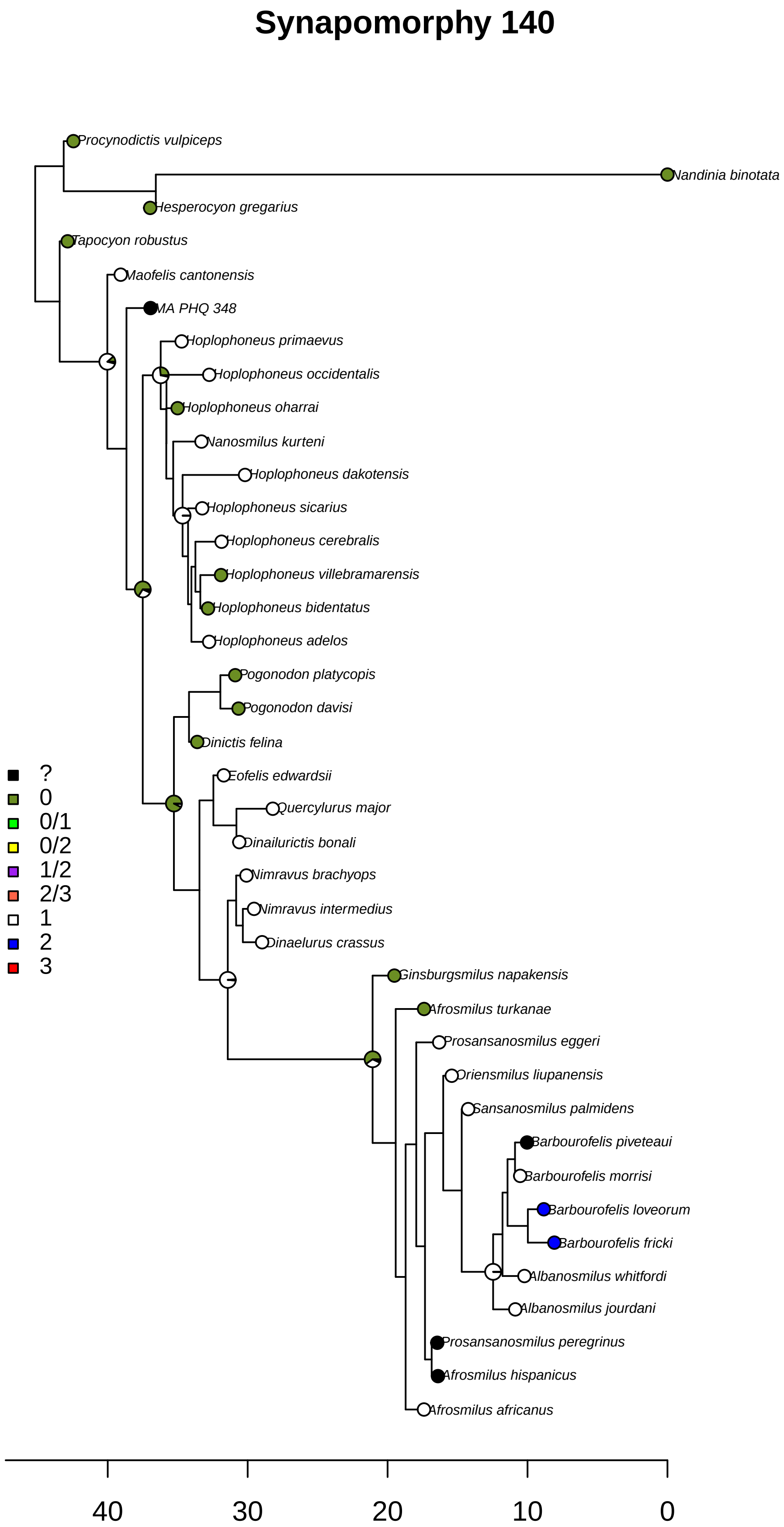
Synapomorphy 140
Procynodictis vulpiceps
Nandinia binotata
Hesperocyon gregarius
Tapocyon robustus
Maofelis cantonensis
MA PHQ 348
Hoplophoneus primaevus
Hoplophoneus occidentalis
Hoplophoneus oharrai
Nanosmilus kurteni
Hoplophoneus dakotensis
Hoplophoneus sicarius
Hoplophoneus cerebralis
Hoplophoneus villebramarensis
Hoplophoneus bidentatus
Hoplophoneus adelos
Pogonodon platycopis
Pogonodon davisi
Dinictis felina
Eofelis edwardsii
Quercylurus major
Dinailurictis bonali
Nimravus brachyops
Nimravus intermedius
Dinaelurus crassus
Ginsburgsmilus napakensis
Afrosmilus turkanae
Prosansanosmilus eggeri
Oriensmilus liupanensis
Sansanosmilus palmidens
Barbourofelis piveteaui
Barbourofelis morrisi
Barbourofelis loveorum
Barbourofelis fricki
Albanosmilus whitfordi
Albanosmilus jourdani
Prosansanosmilus peregrinus
Afrosmilus hispanicus
Afrosmilus africanus
?
0
0/1
0/2
1/2
2/3
1
2
3
40
30
20
10
0

Synapomorphy 148

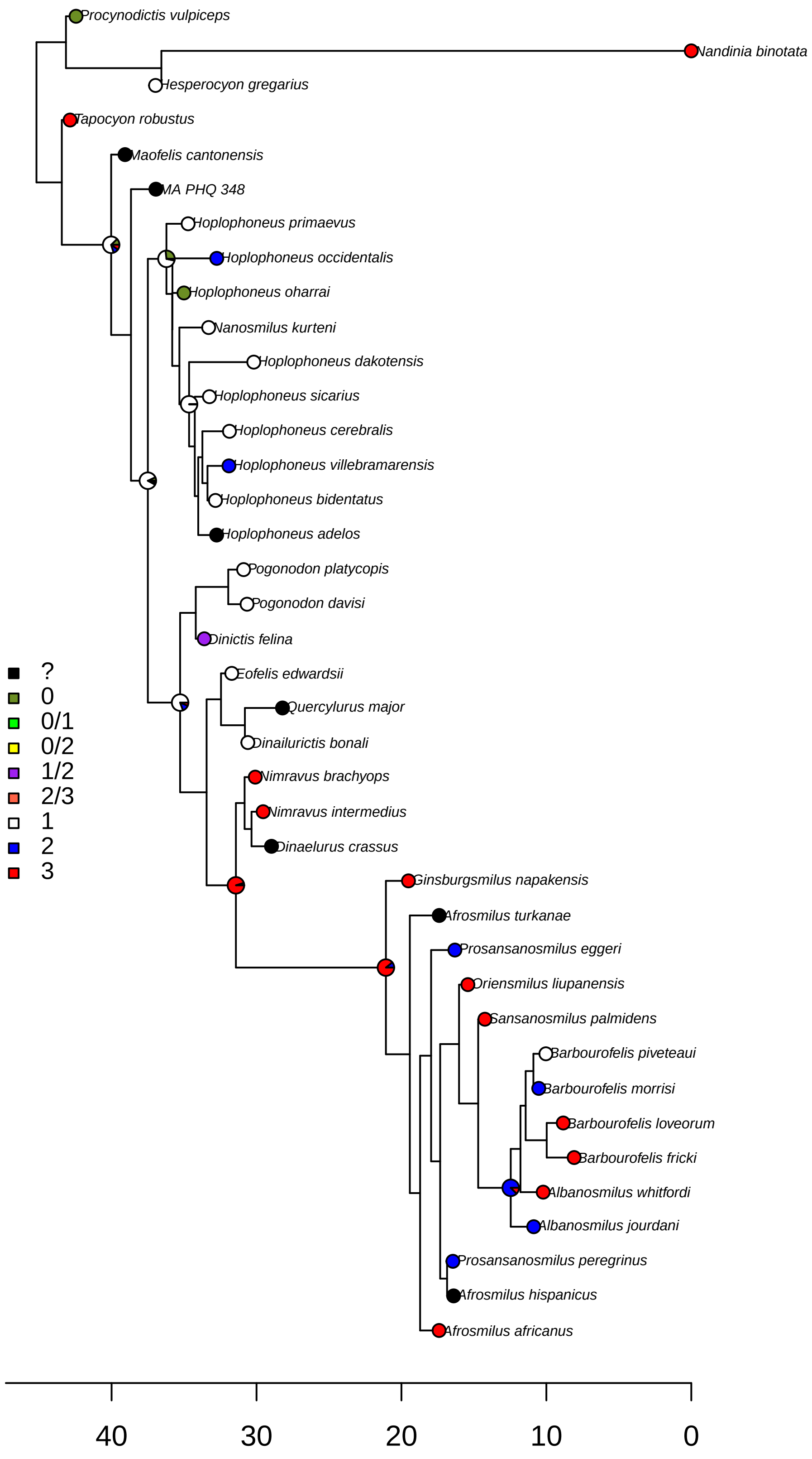

Synapomorphy 151

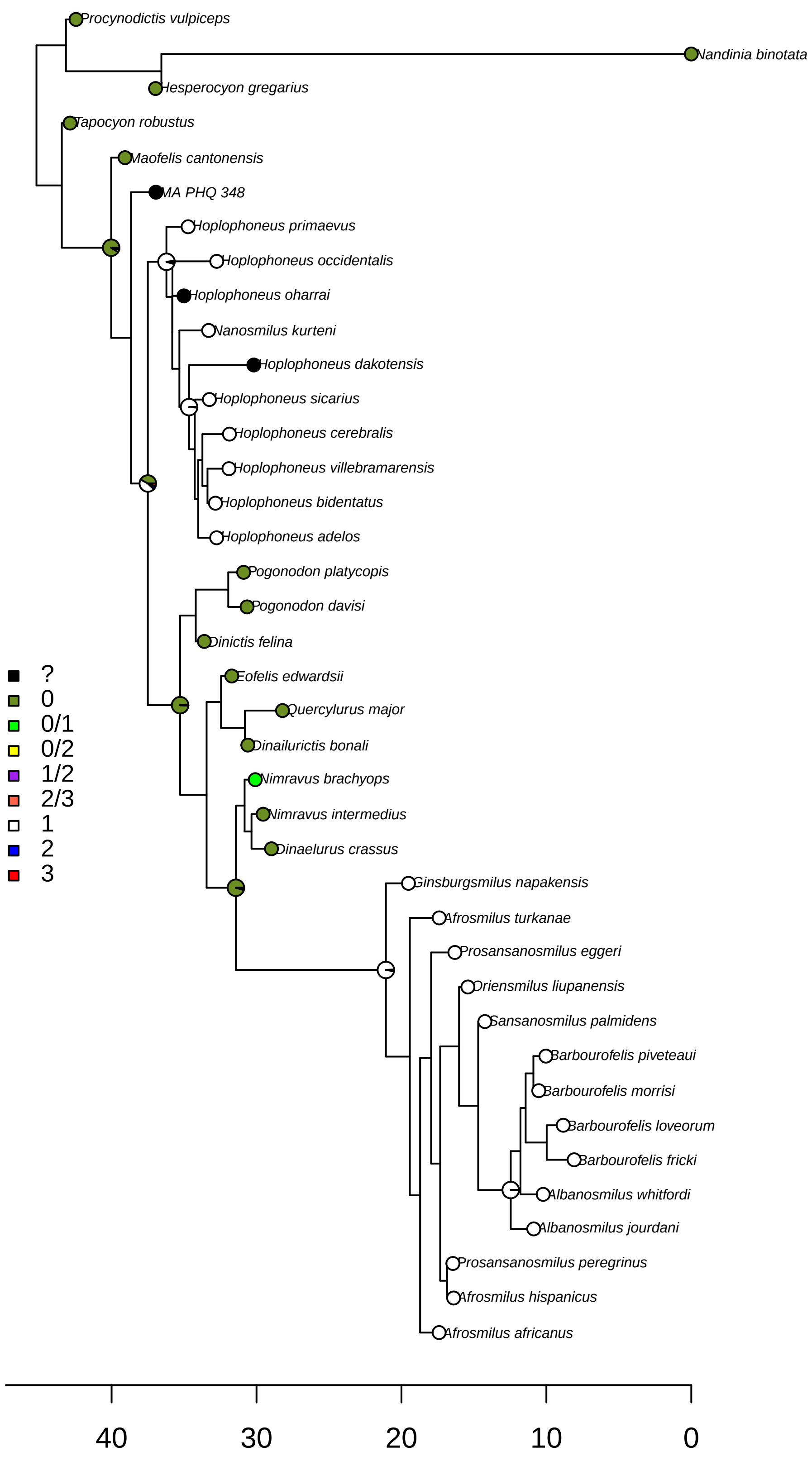

Synapomorphy 153

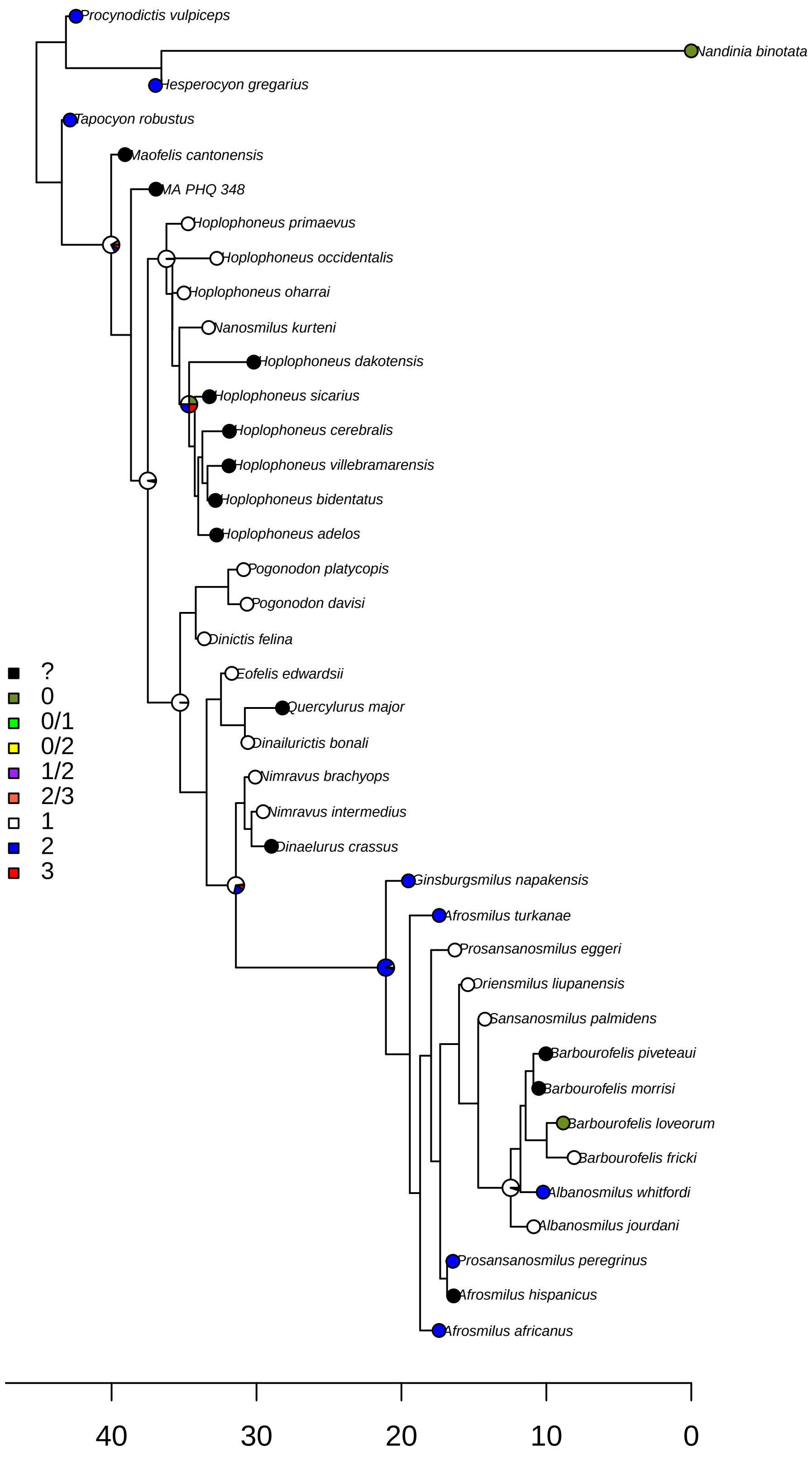

Synapomorphy 154

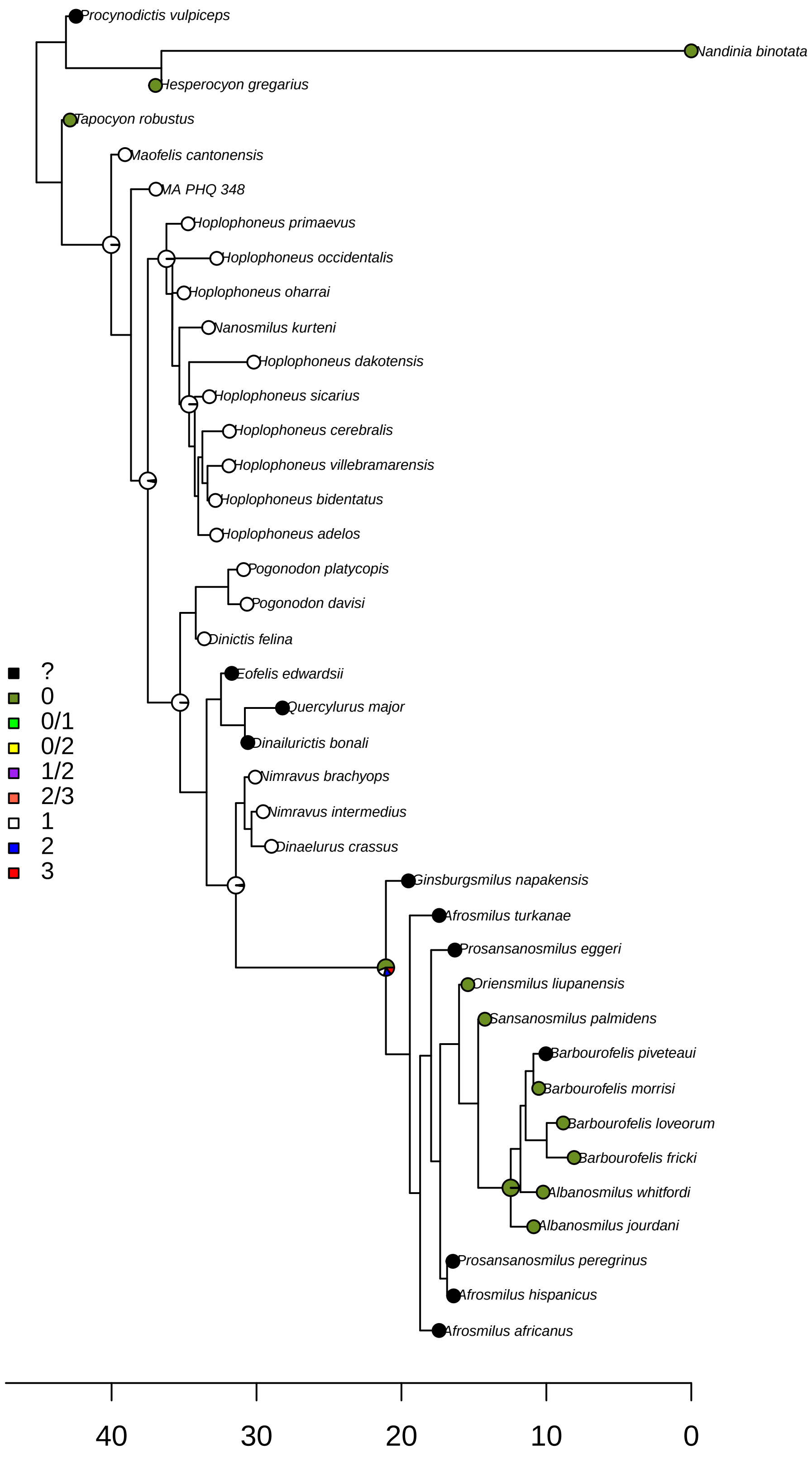

Synapomorphy 155

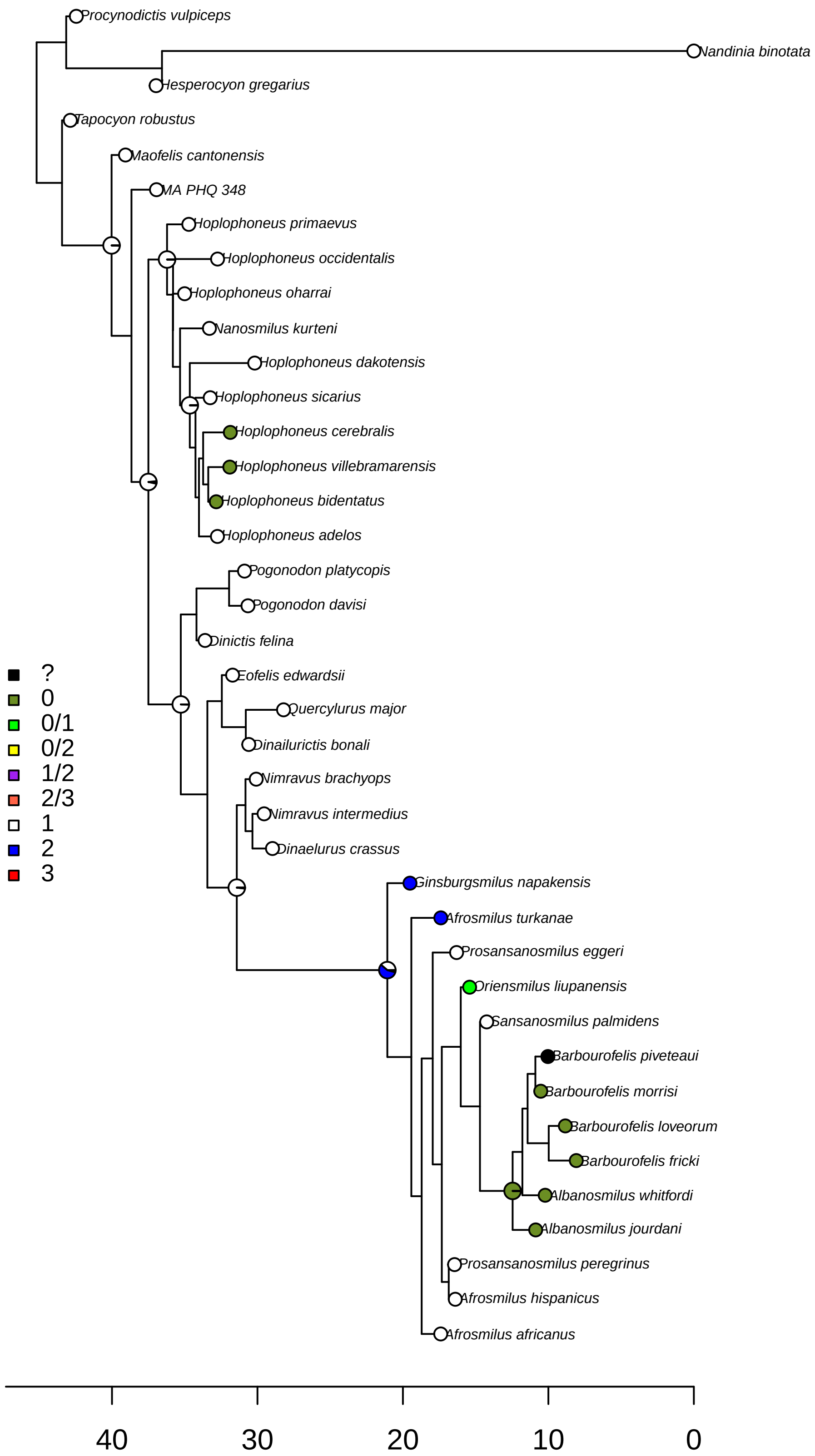

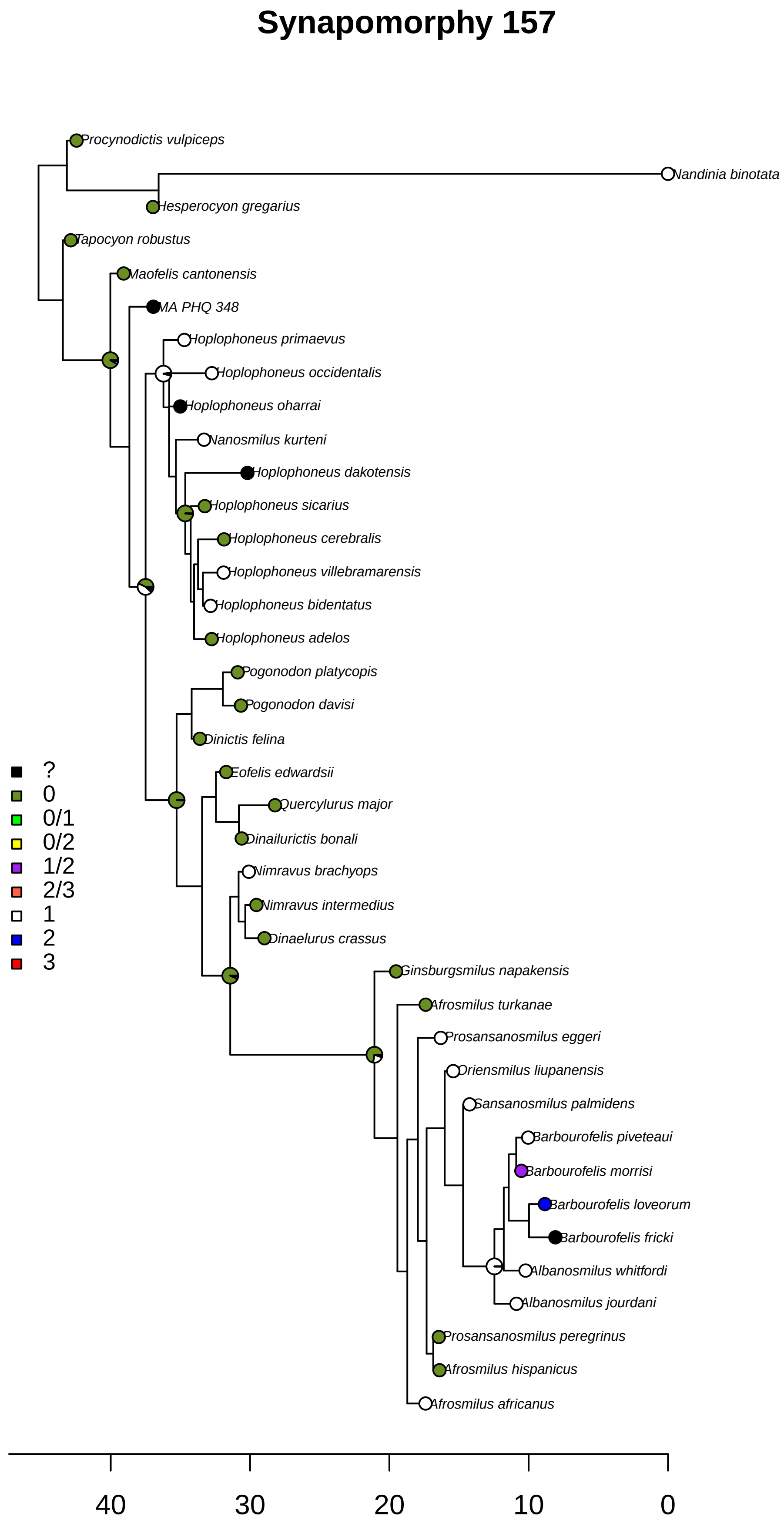
Synapomorphy 157
Procynodictis vulpiceps
Nandinia binotata
Hesperocyon gregarius
Tapocyon robustus
Maofelis cantonensis
MA PHQ 348
Hoplophoneus primaevus
Hoplophoneus occidentalis
Hoplophoneus oharrai
Nanosmilus kurteni
Hoplophoneus dakotensis
Hoplophoneus sicarius
Hoplophoneus cerebralis
Hoplophoneus villebramarensis
Hoplophoneus bidentatus
Hoplophoneus adelos
Pogonodon platycopis
Pogonodon davisi
Dinictis felina
Eofelis edwardsii
Quercylurus major
Dinailurictis bonali
Nimravus brachyops
Nimravus intermedius
Dinaelurus crassus
Ginsburgsmilus napakensis
Afrosmilus turkanae
Prosansanosmilus eggeri
Oriensmilus liupanensis
Sansanosmilus palmidens
Barbourofelis piveteaui
Barbourofelis morrisi
Barbourofelis loveorum
Barbourofelis fricki
Albanosmilus whitfordi
Albanosmilus jourdani
Prosansanosmilus peregrinus
Afrosmilus hispanicus
Afrosmilus africanus
?
0
0/1
0/2
1/2
2/3
1
2
3
40
30
20
10
0

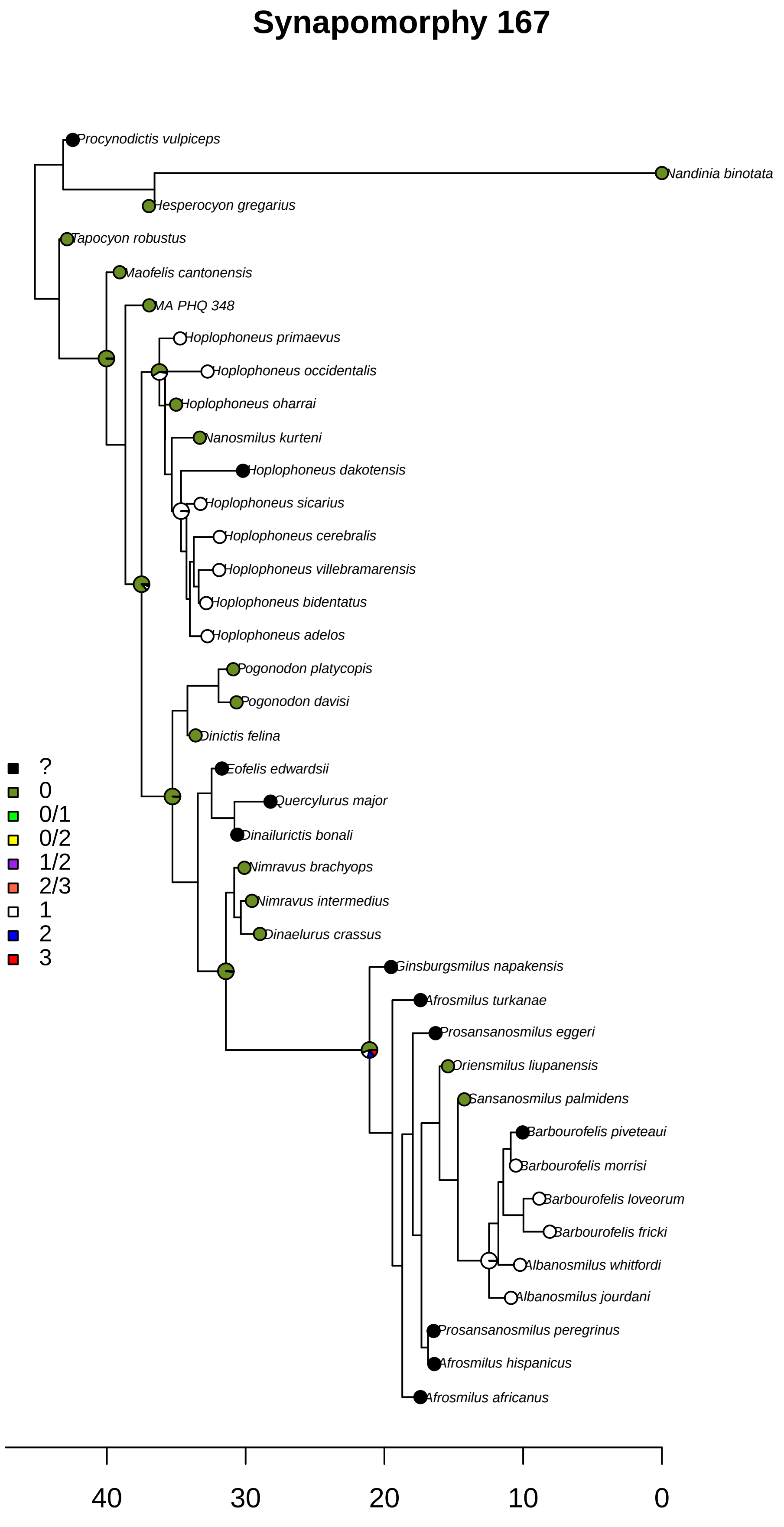
Synapomorphy 167
Procynodictis vulpiceps
Nandinia binotata
Hesperocyon gregarius
Tapocyon robustus
Maofelis cantonensis
MA PHQ 348
Hoplophoneus primaevus
Hoplophoneus occidentalis
Hoplophoneus oharrai
Nanosmilus kurteni
Hoplophoneus dakotensis
Hoplophoneus sicarius
Hoplophoneus cerebralis
Hoplophoneus villebramarensis
Hoplophoneus bidentatus
Hoplophoneus adelos
Pogonodon platycopis
Pogonodon davisi
Dinictis felina
Eofelis edwardsii
Quercylurus major
Dinailurictis bonali
Nimravus brachyops
Nimravus intermedius
Dinaelurus crassus
Ginsburgsmilus napakensis
Afrosmilus turkanae
Prosansanosmilus eggeri
Oriensmilus liupanensis
Sansanosmilus palmidens
Barbourofelis piveteaui
Barbourofelis morrisi
Barbourofelis loveorum
Barbourofelis fricki
Albanosmilus whitfordi
Albanosmilus jourdani
Prosansanosmilus peregrinus
Afrosmilus hispanicus
Afrosmilus africanus
?
0
0/1
0/2
1/2
2/3
1
2
3
40
30
20
10
0

Synapomorphy 173

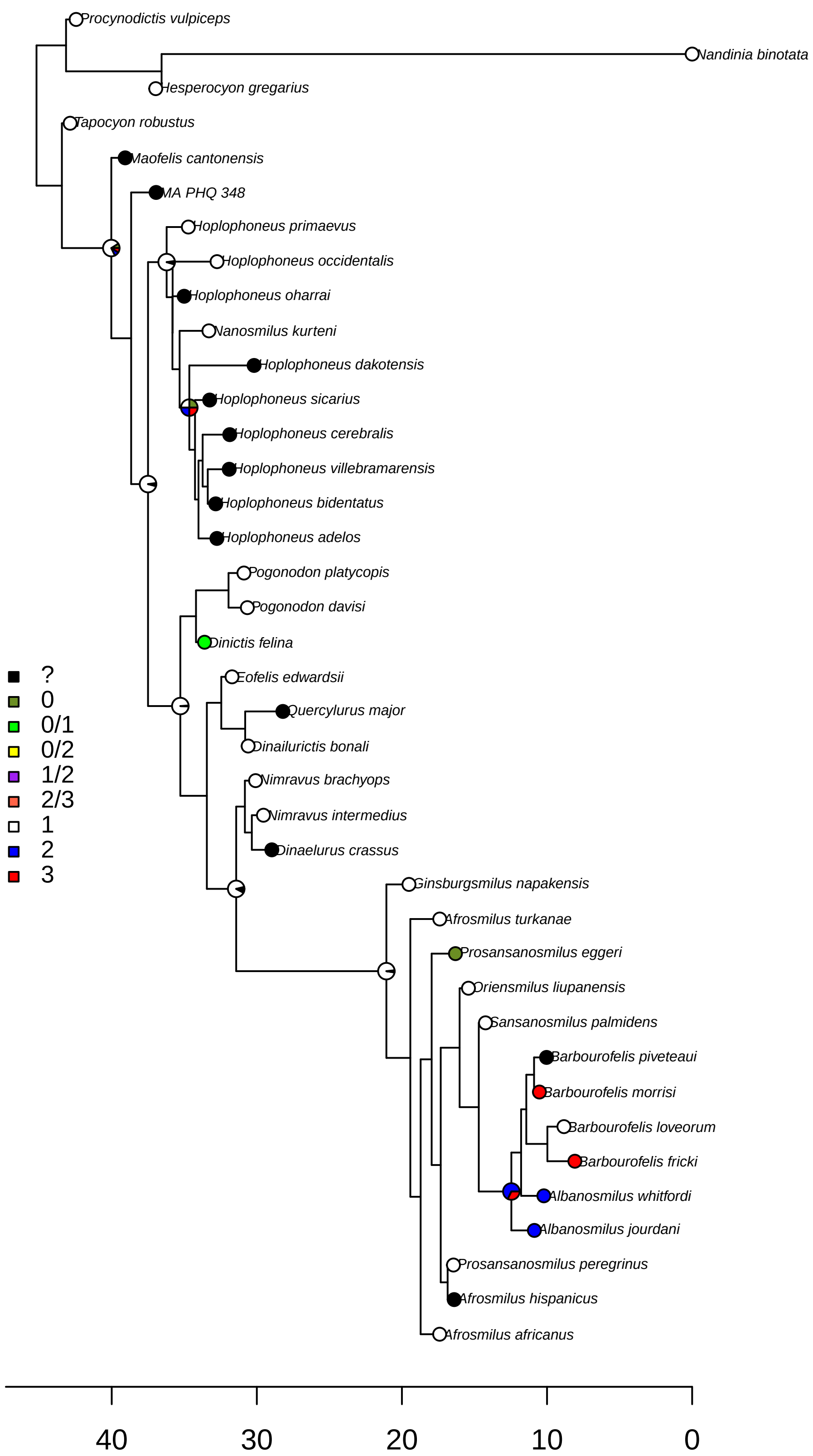

Synapomorphy 176

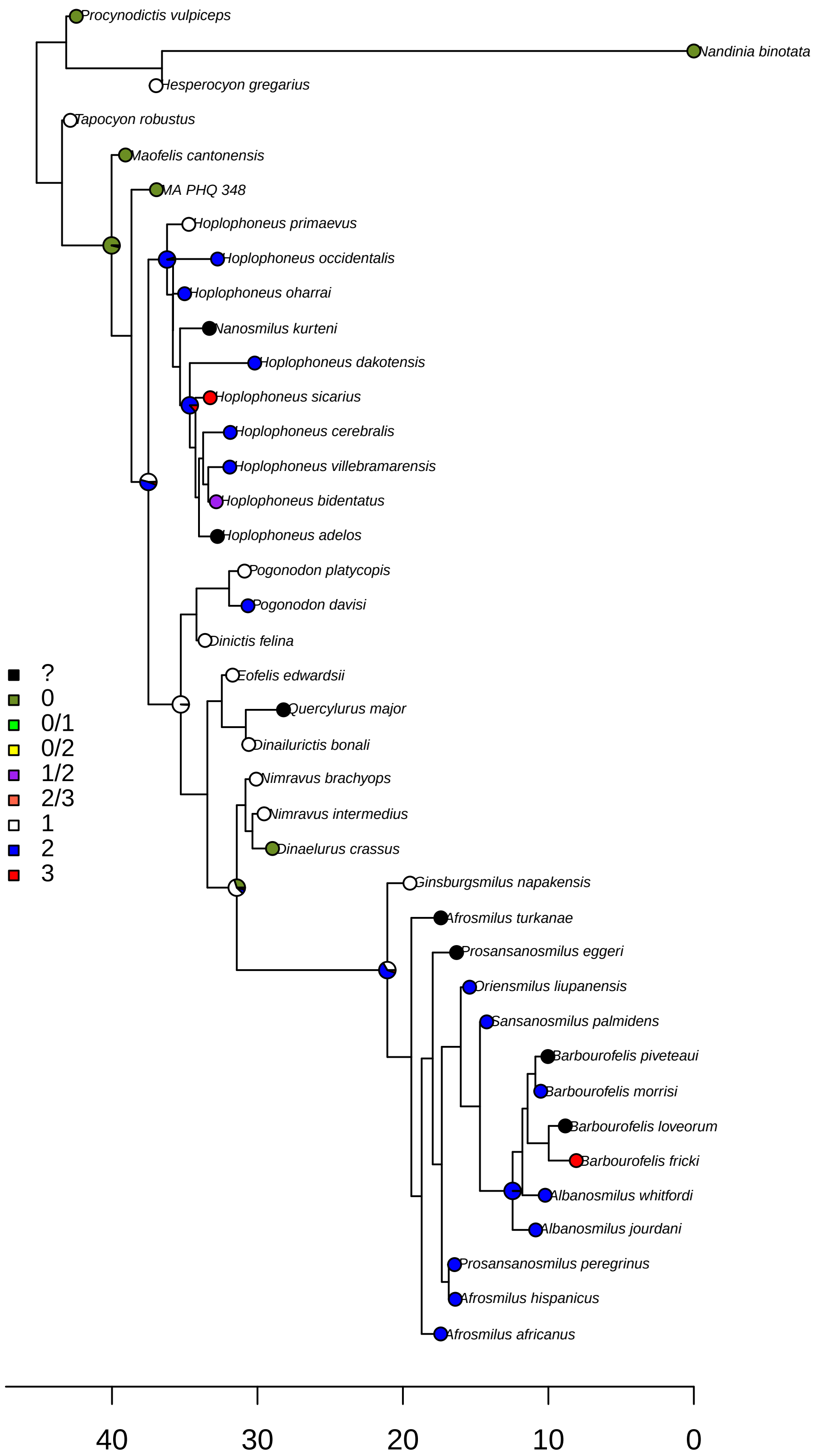

Synapomorphy 177

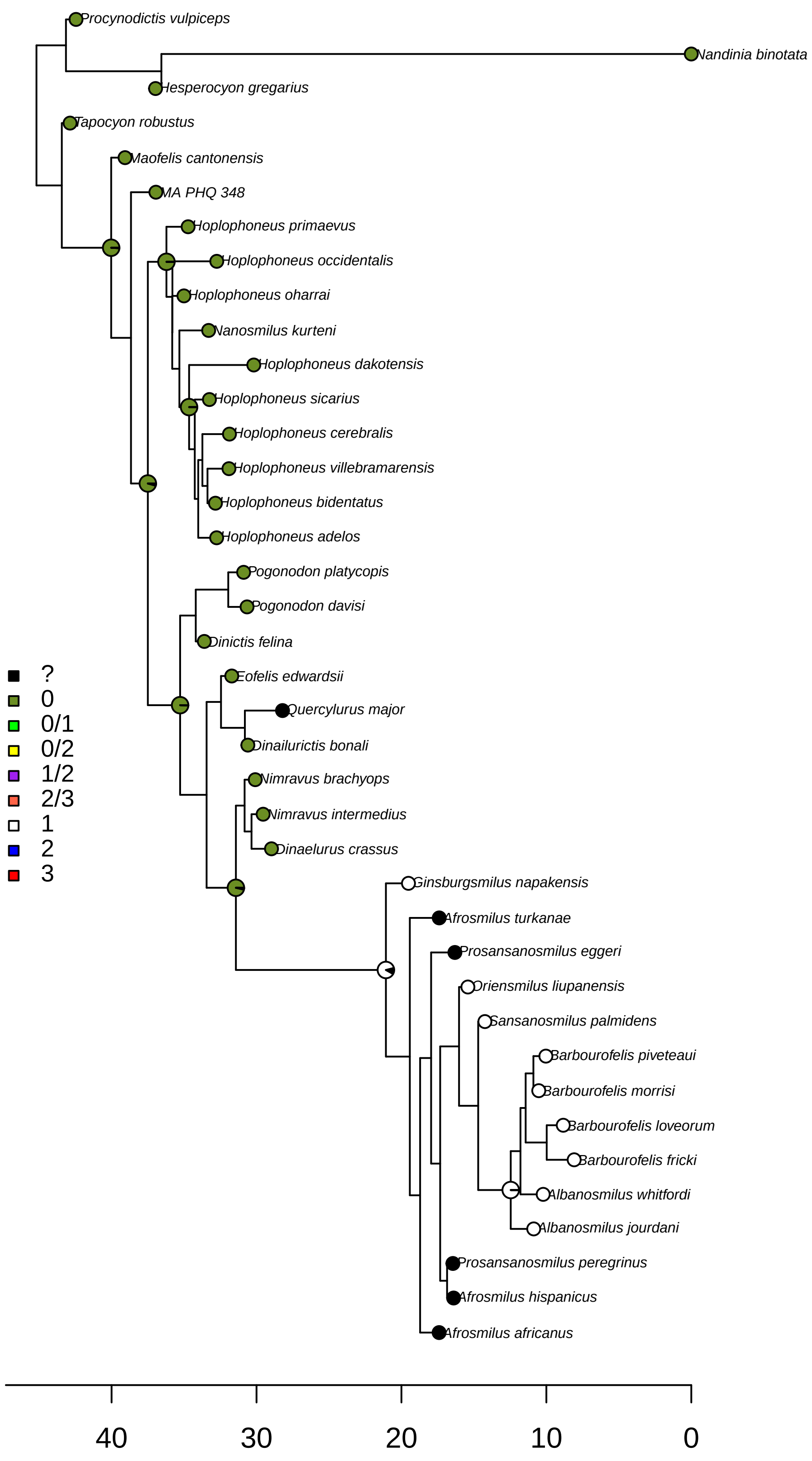

Synapomorphy 180

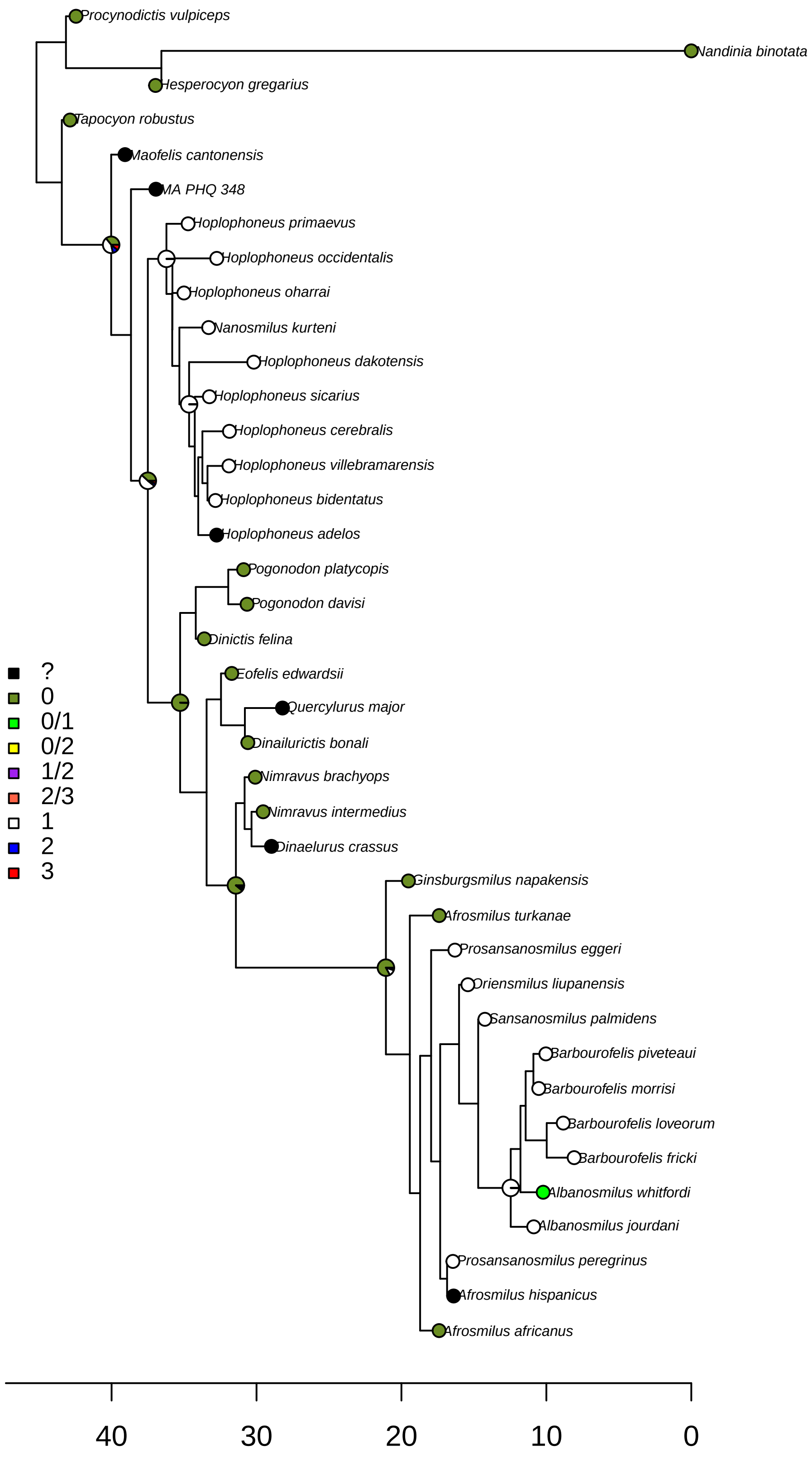

Synapomorphy 181

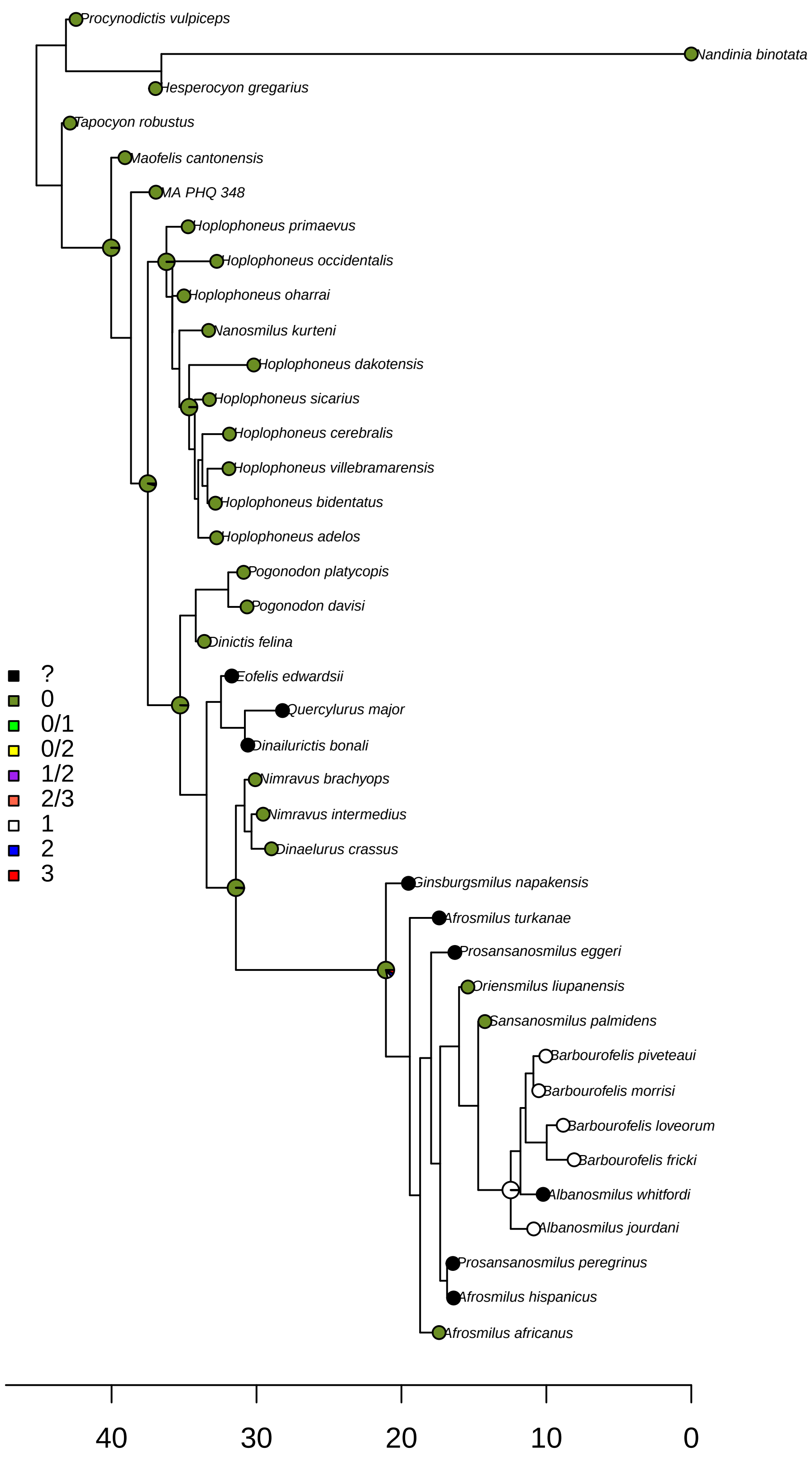

Synapomorphy 183

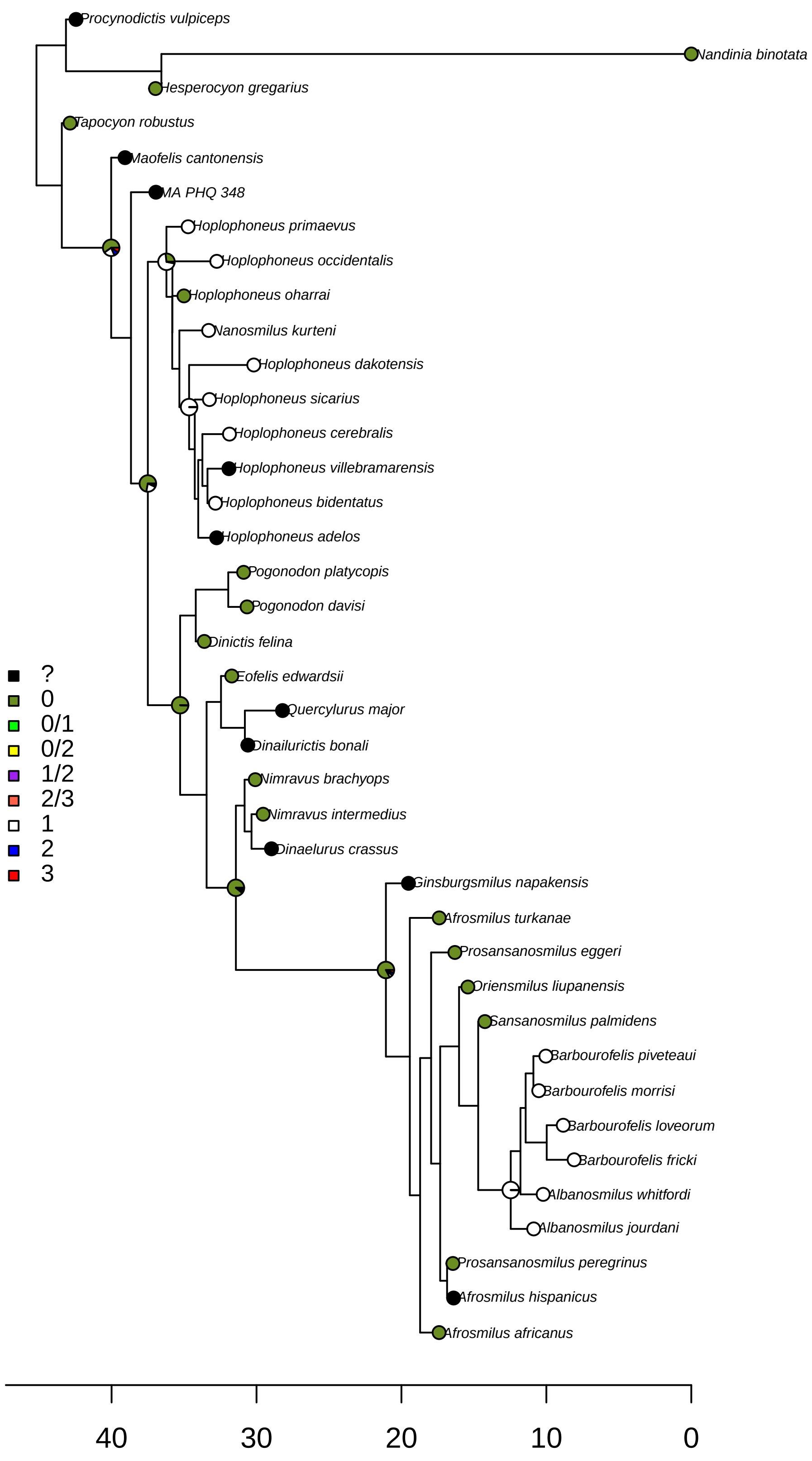

Synapomorphy 184

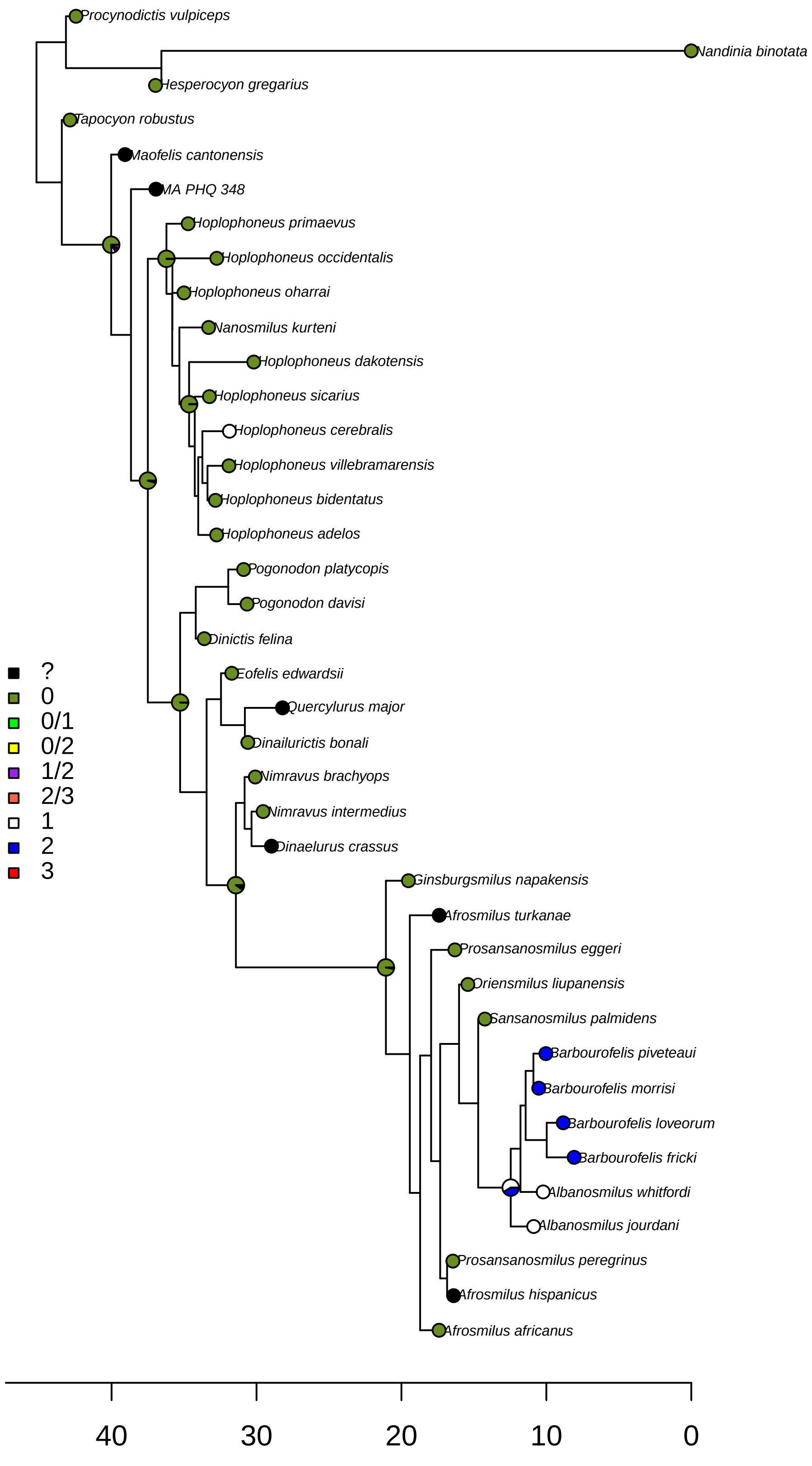

Synapomorphy 187

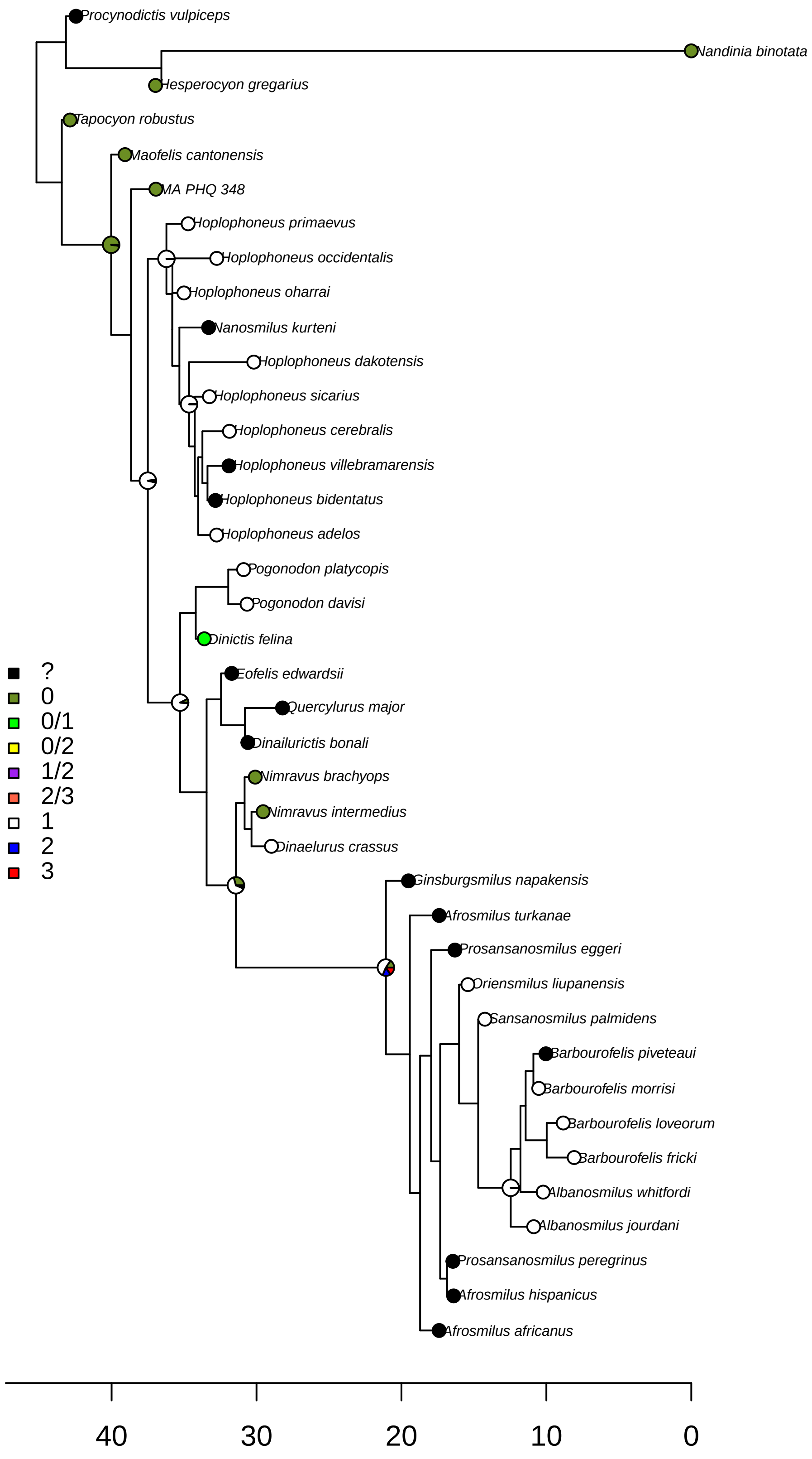

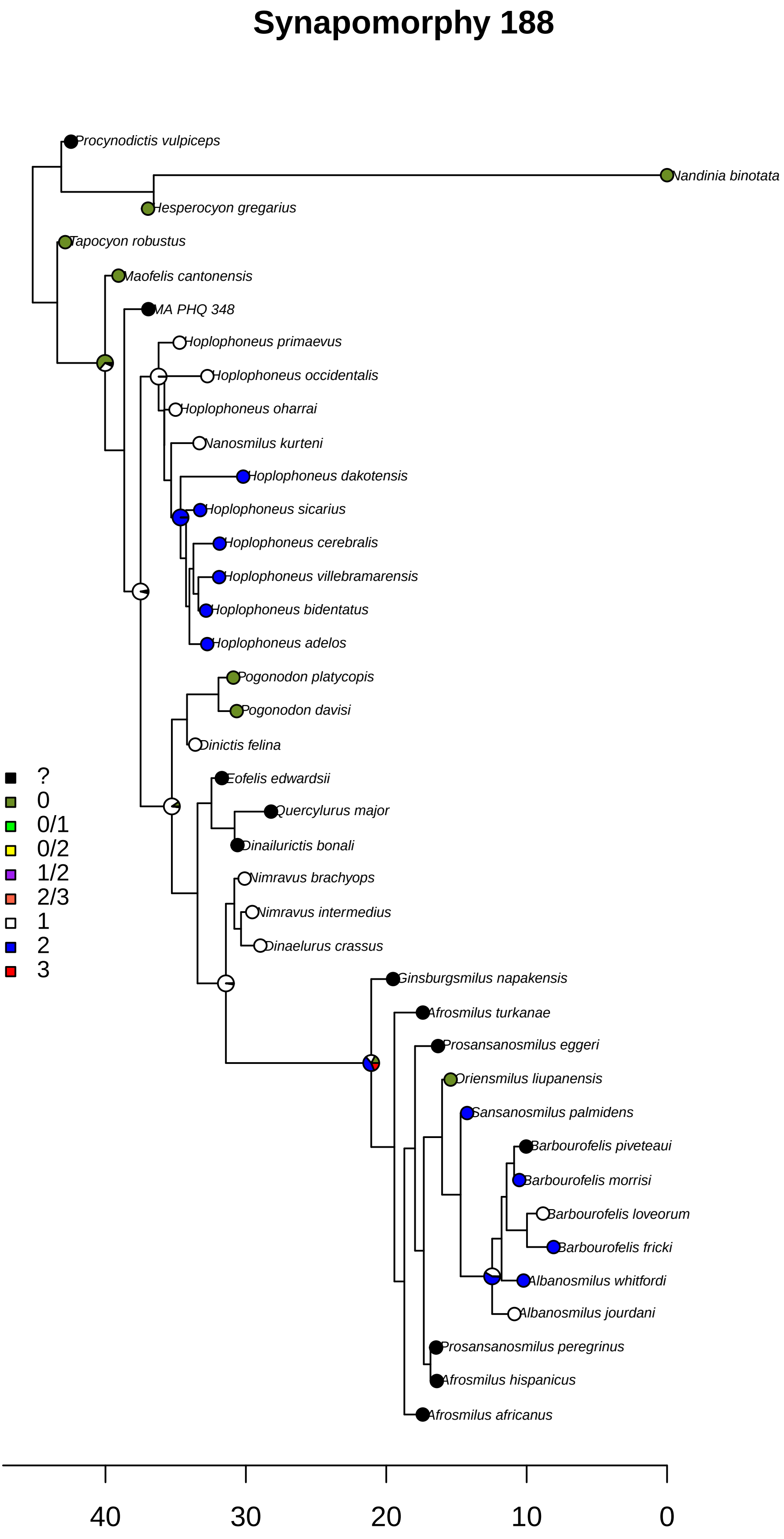
Synapomorphy 188
Procynodictis vulpiceps
Nandinia binotata
Hesperocyon gregarius
Tapocyon robustus
Maofelis cantonensis
MA PHQ 348
Hoplophoneus primaevus
Hoplophoneus occidentalis
Hoplophoneus oharrai
Nanosmilus kurteni
Hoplophoneus dakotensis
Hoplophoneus sicarius
Hoplophoneus cerebralis
Hoplophoneus villebramarensis
Hoplophoneus bidentatus
Hoplophoneus adelos
Pogonodon platycopis
Pogonodon davisi
Dinictis felina
Eofelis edwardsii
Quercylurus major
Dinailurictis bonali
Nimravus brachyops
Nimravus intermedius
Dinaelurus crassus
Ginsburgsmilus napakensis
Afrosmilus turkanae
Prosansanosmilus eggeri
Oriensmilus liupanensis
Sansanosmilus palmidens
Barbourofelis piveteaui
Barbourofelis morrisi
Barbourofelis loveorum
Barbourofelis fricki
Albanosmilus whitfordi
Albanosmilus jourdani
Prosansanosmilus peregrinus
Afrosmilus hispanicus
Afrosmilus africanus
?
0
0/1
0/2
1/2
2/3
1
2
3
40
30
20
10
0

Synapomorphy 189

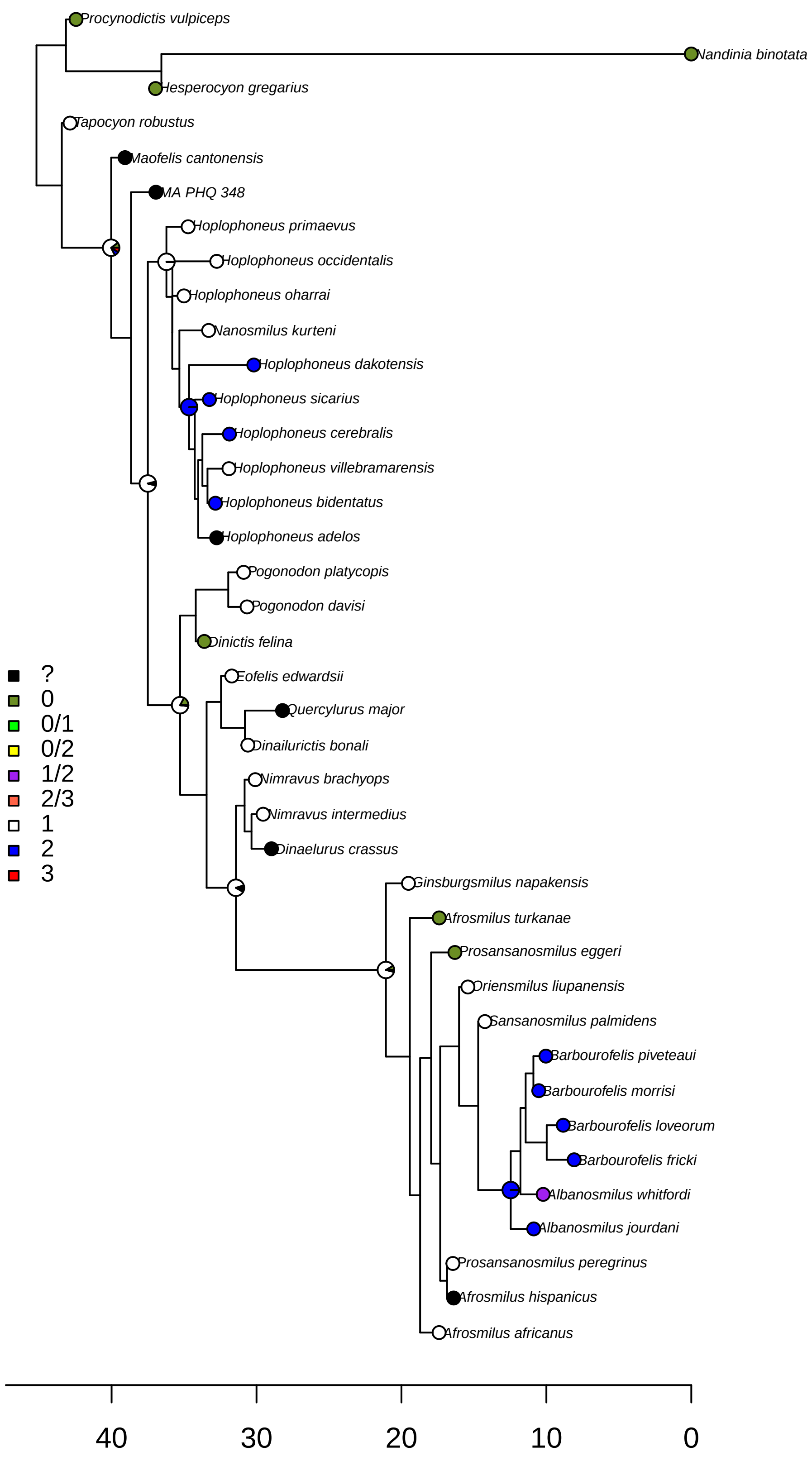

Synapomorphy 190

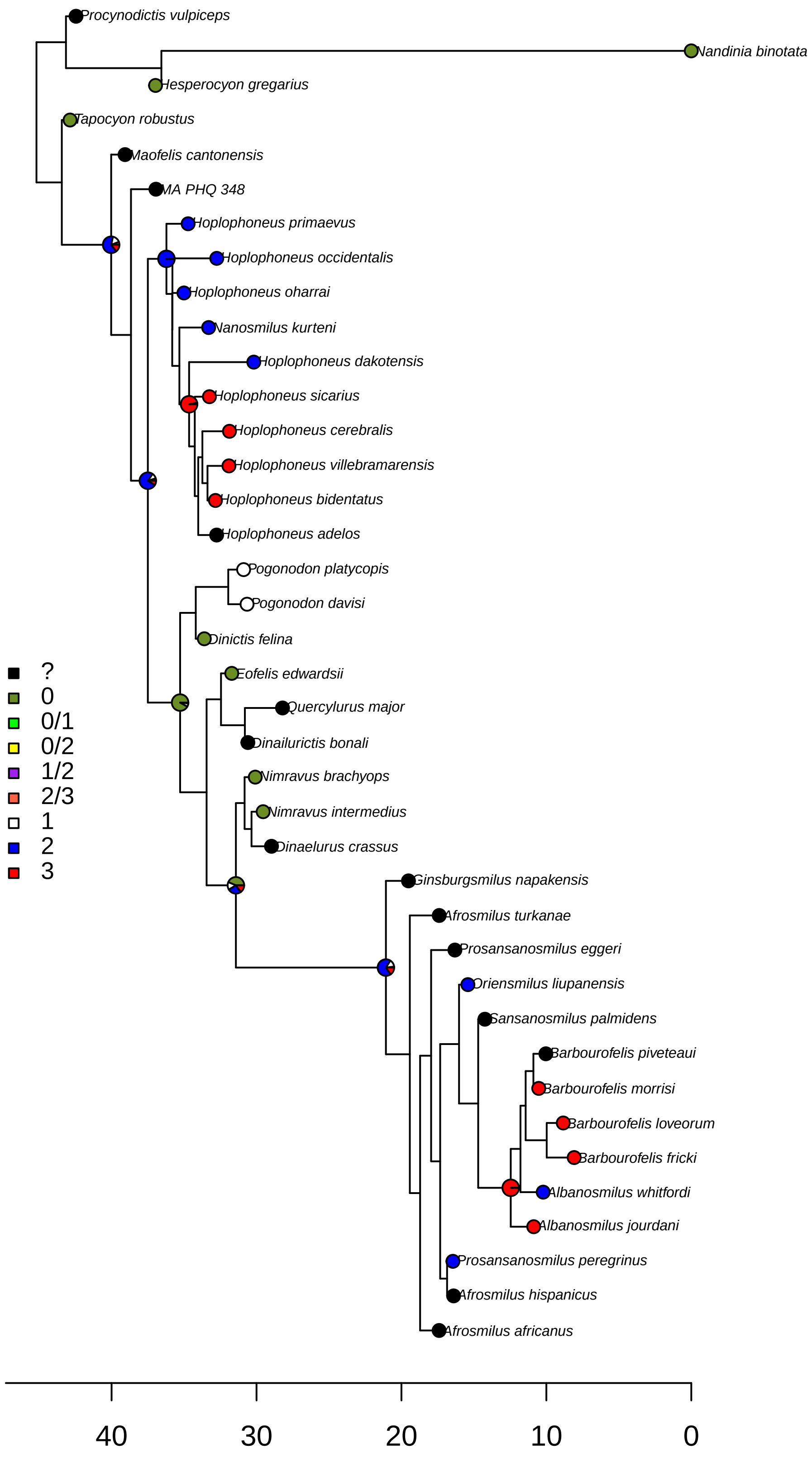

Synapomorphy 192

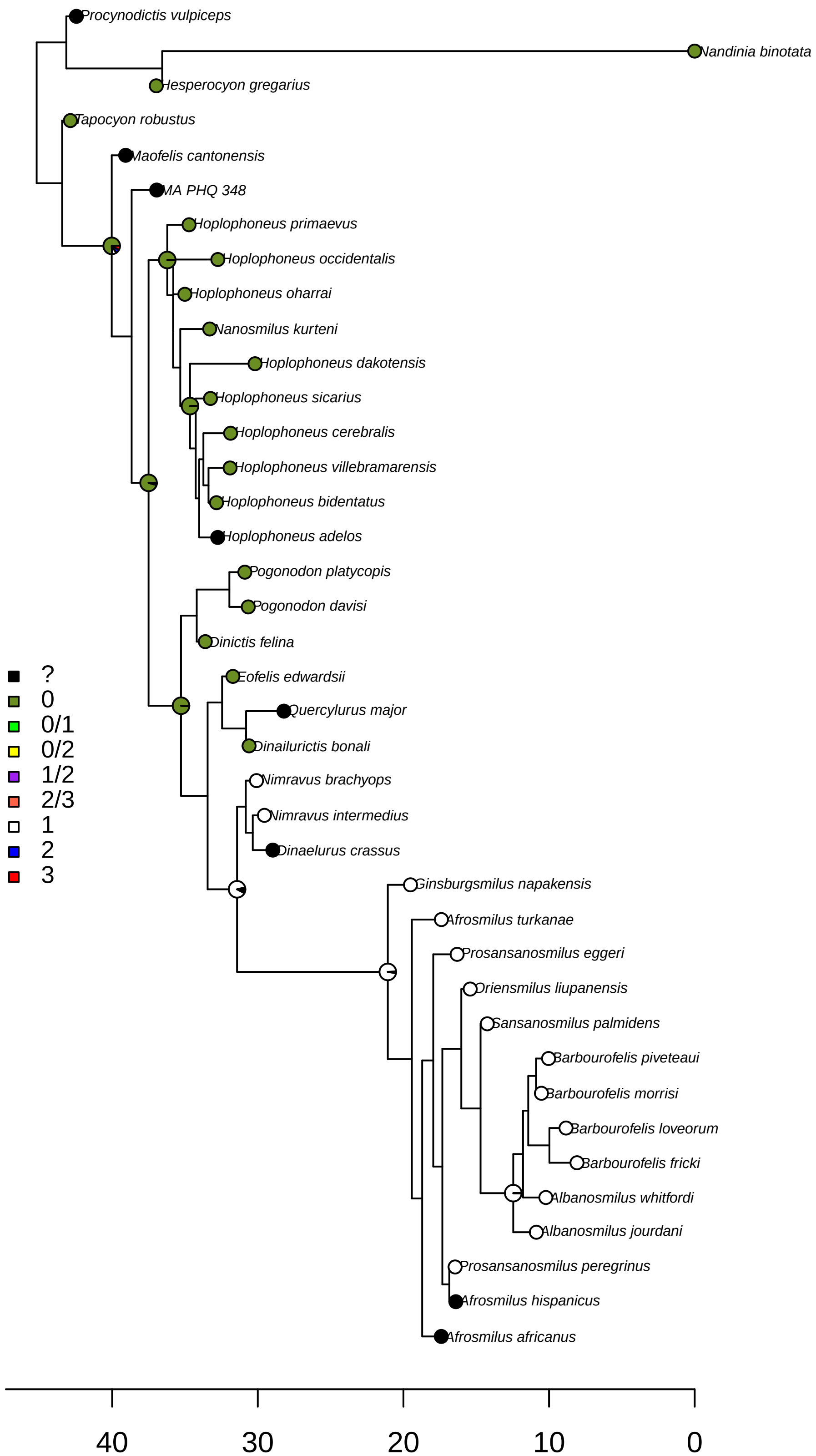

Synapomorphy 193

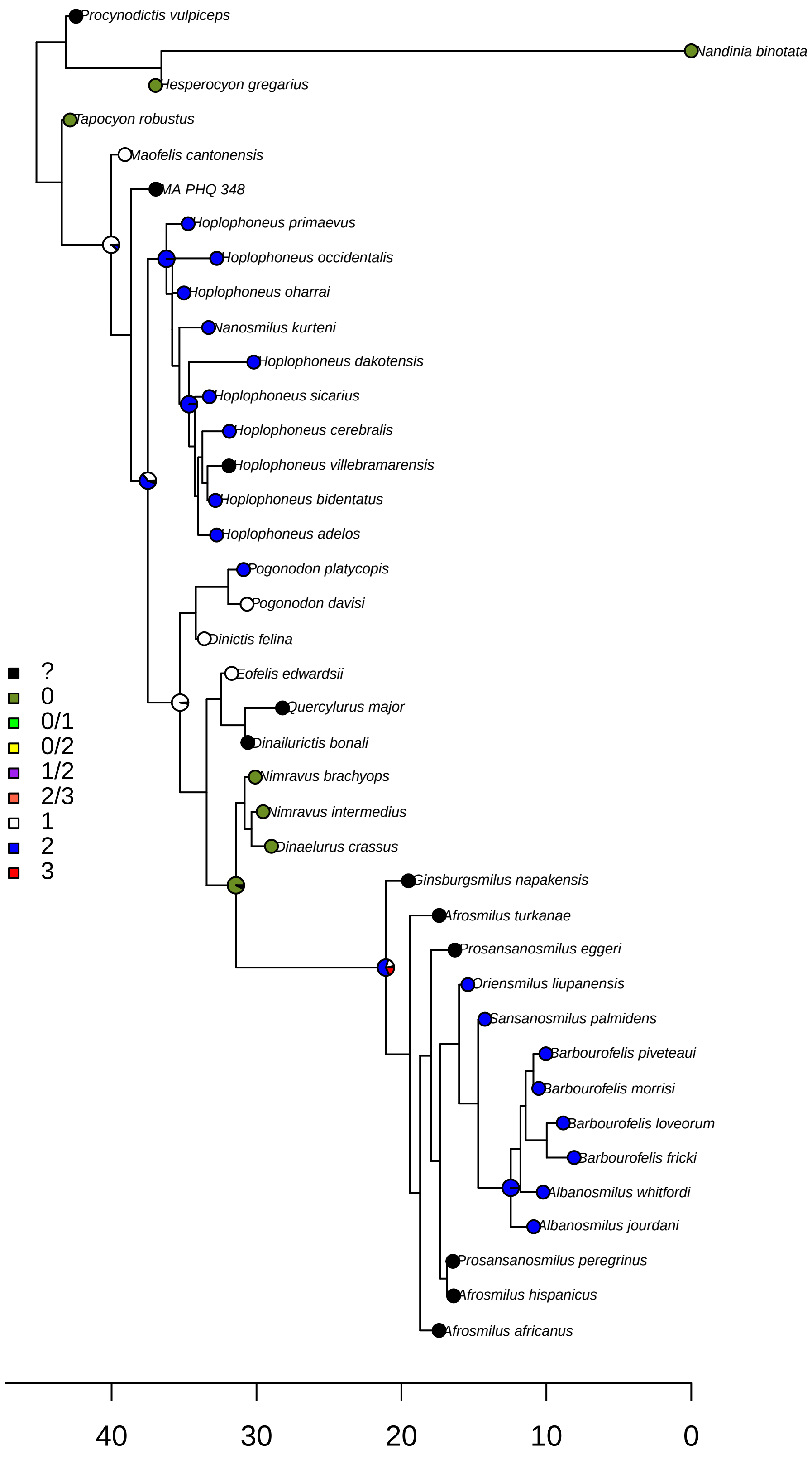

Synapomorphy 196

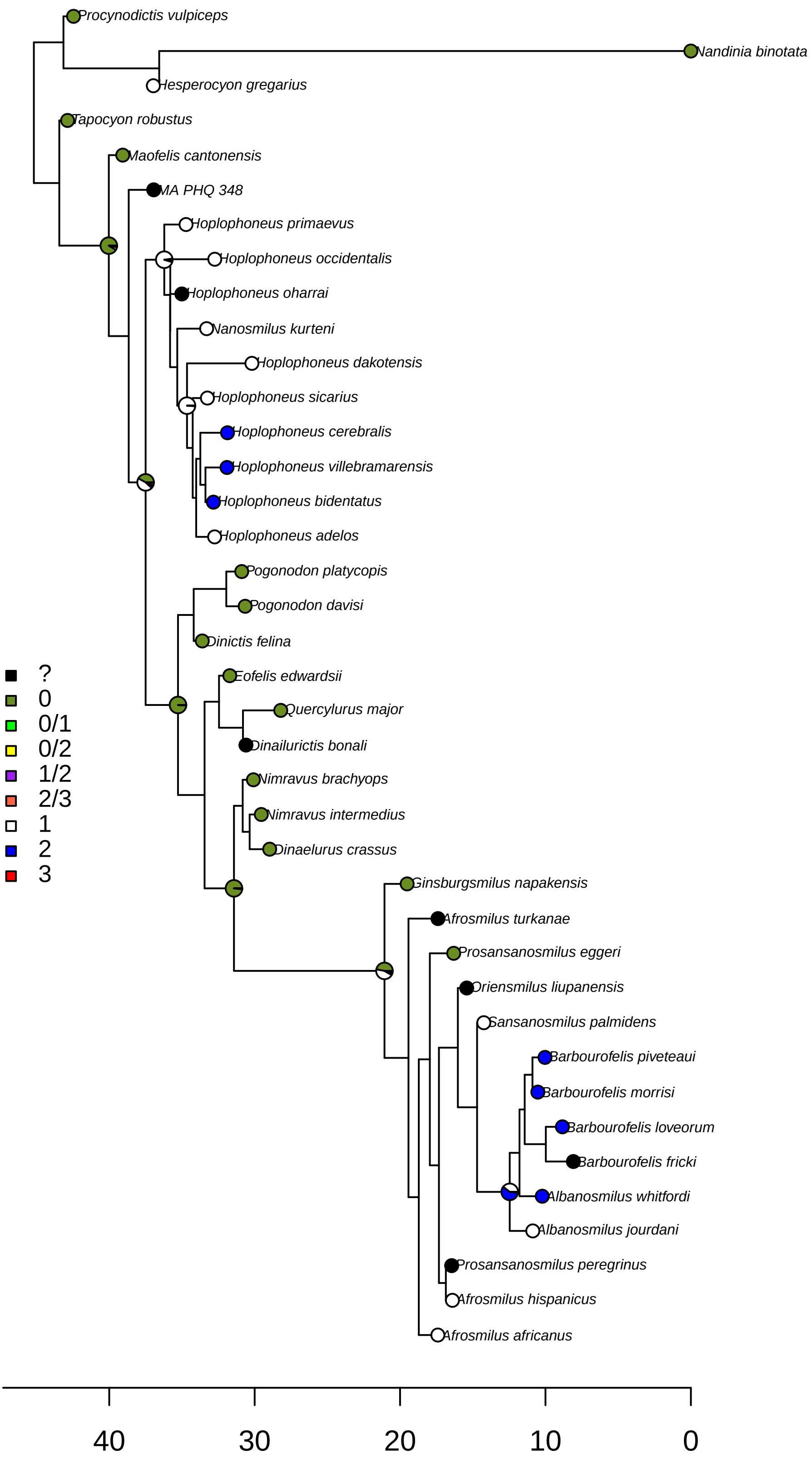

Synapomorphy 197

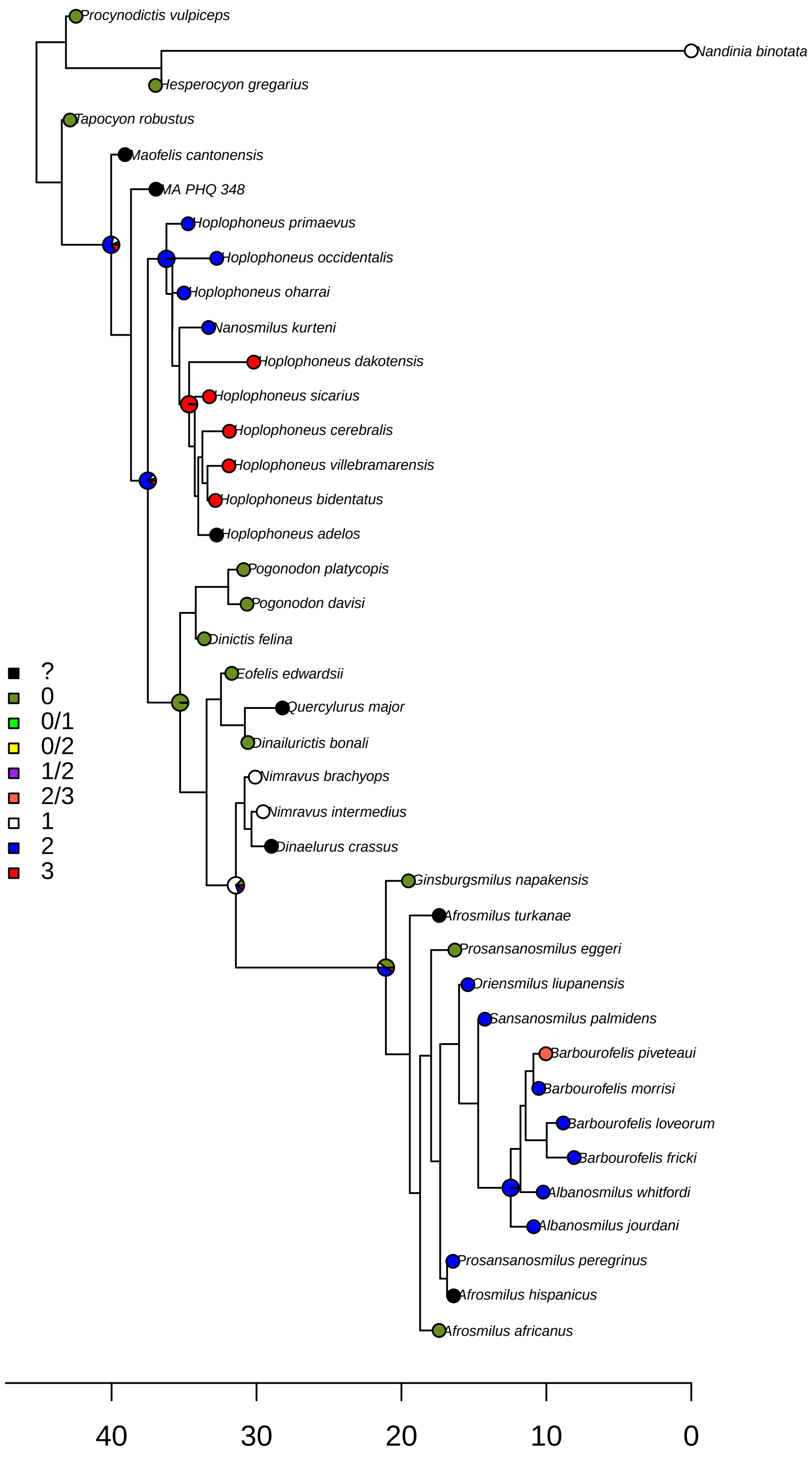

Synapomorphy 200

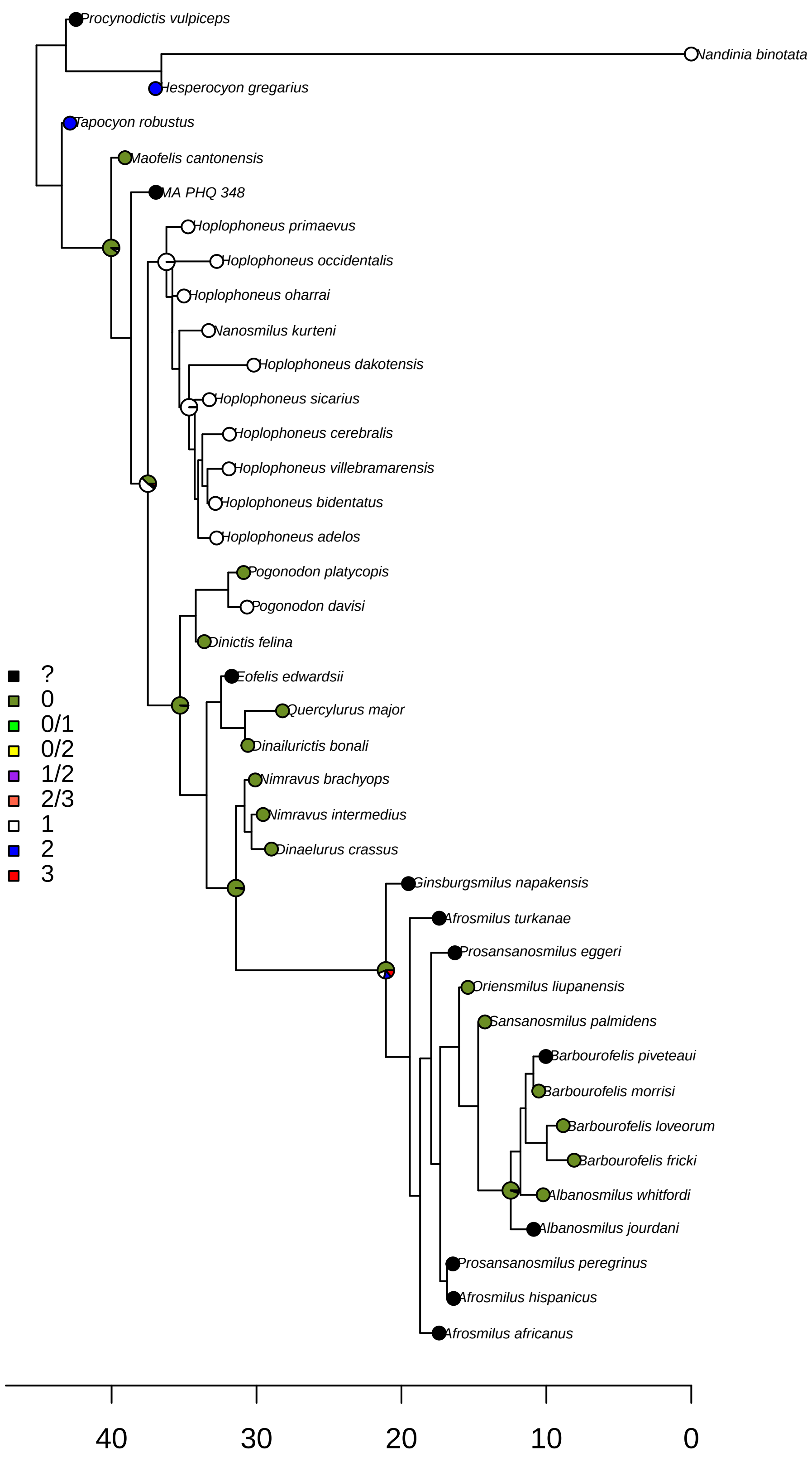

Synapomorphy 202

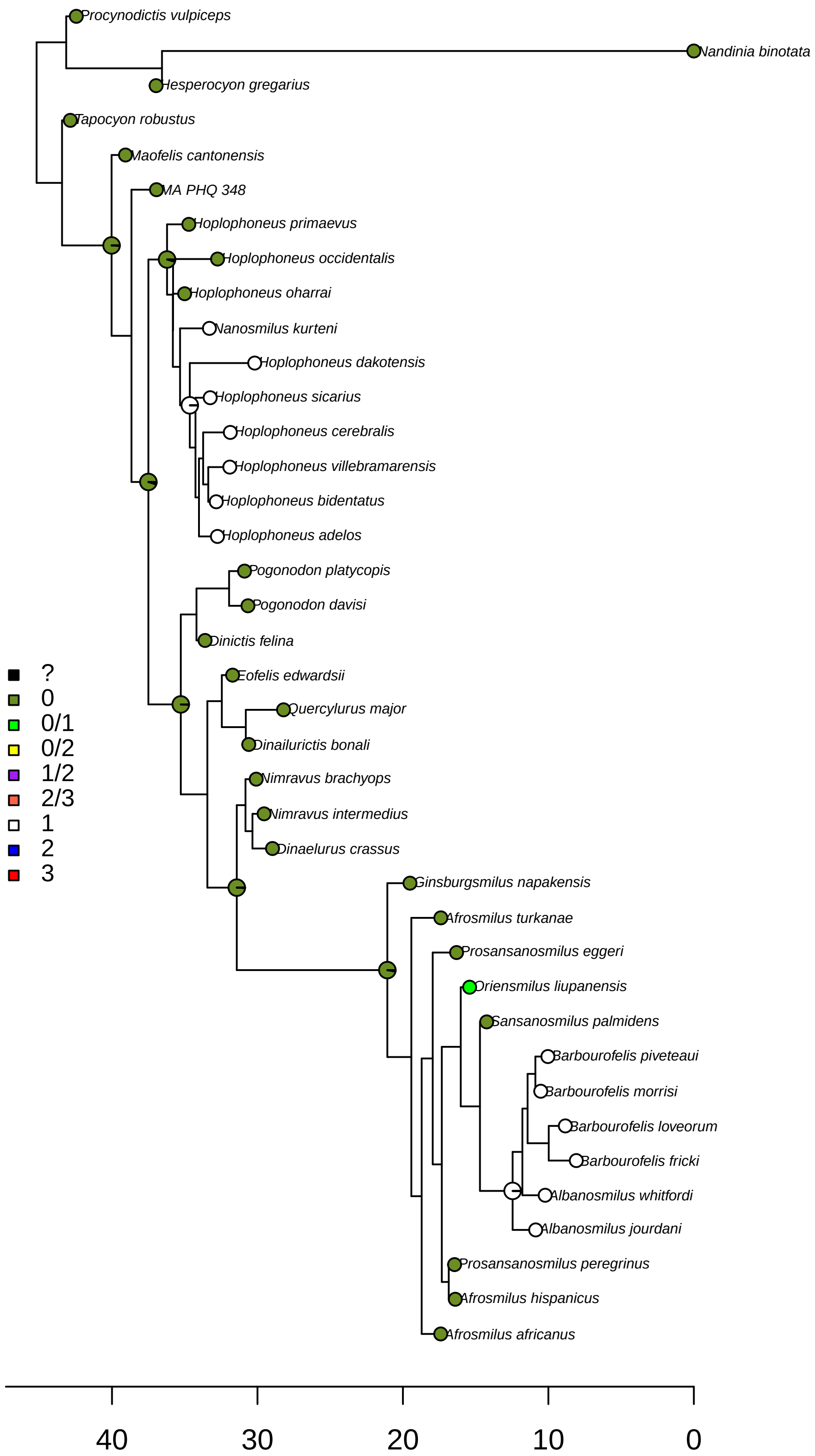

Synapomorphy 207

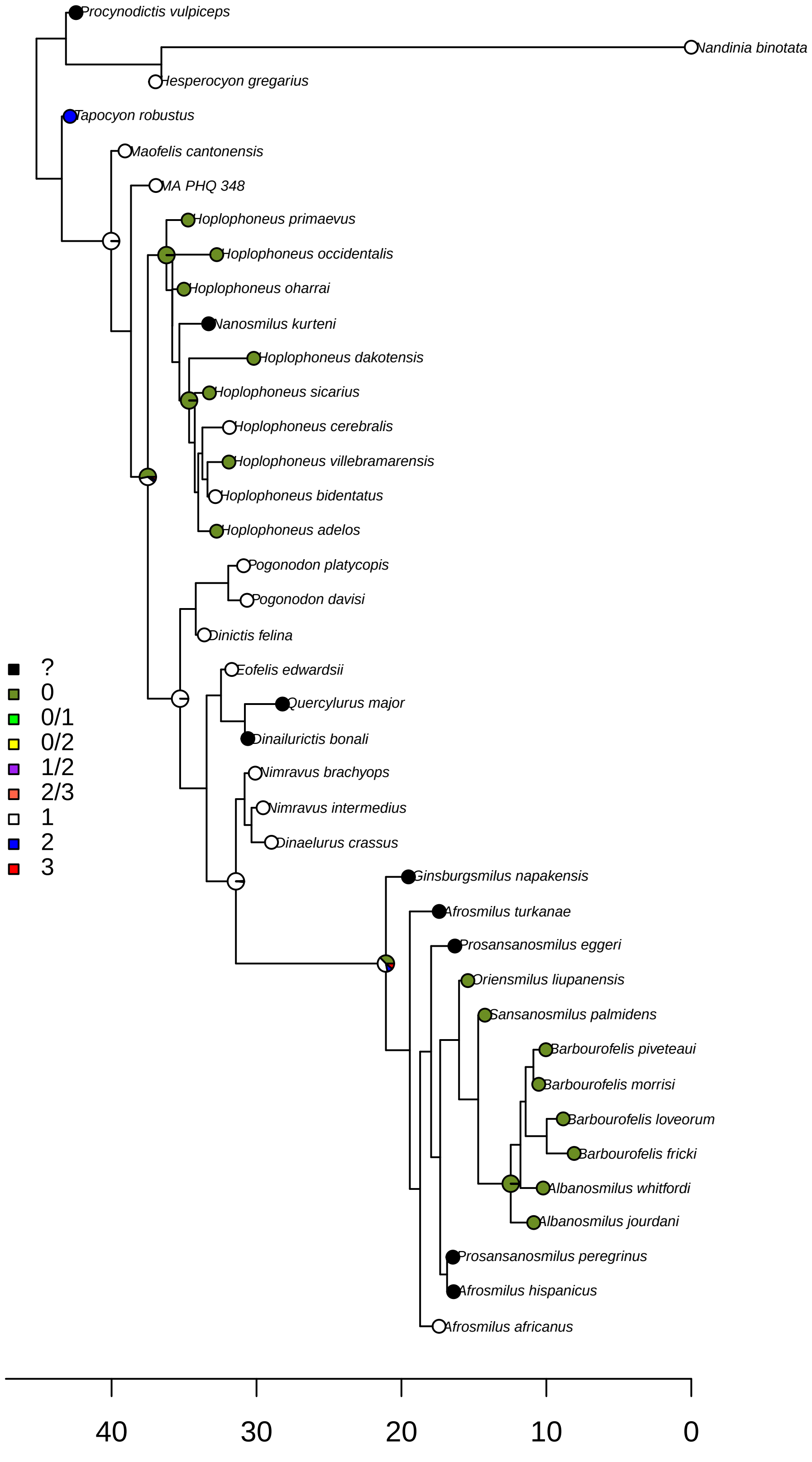

Synapomorphy 211

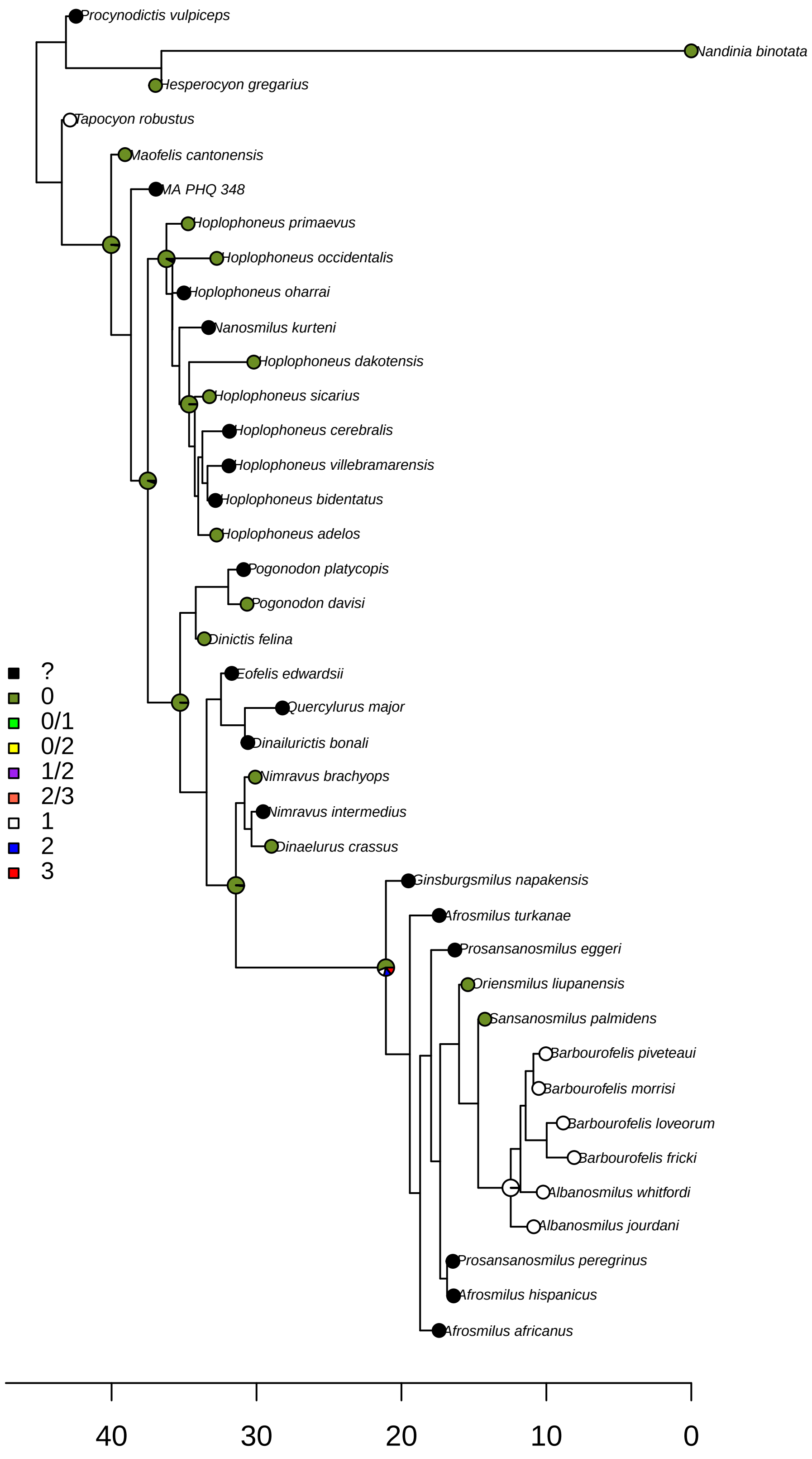

Synapomorphy 213

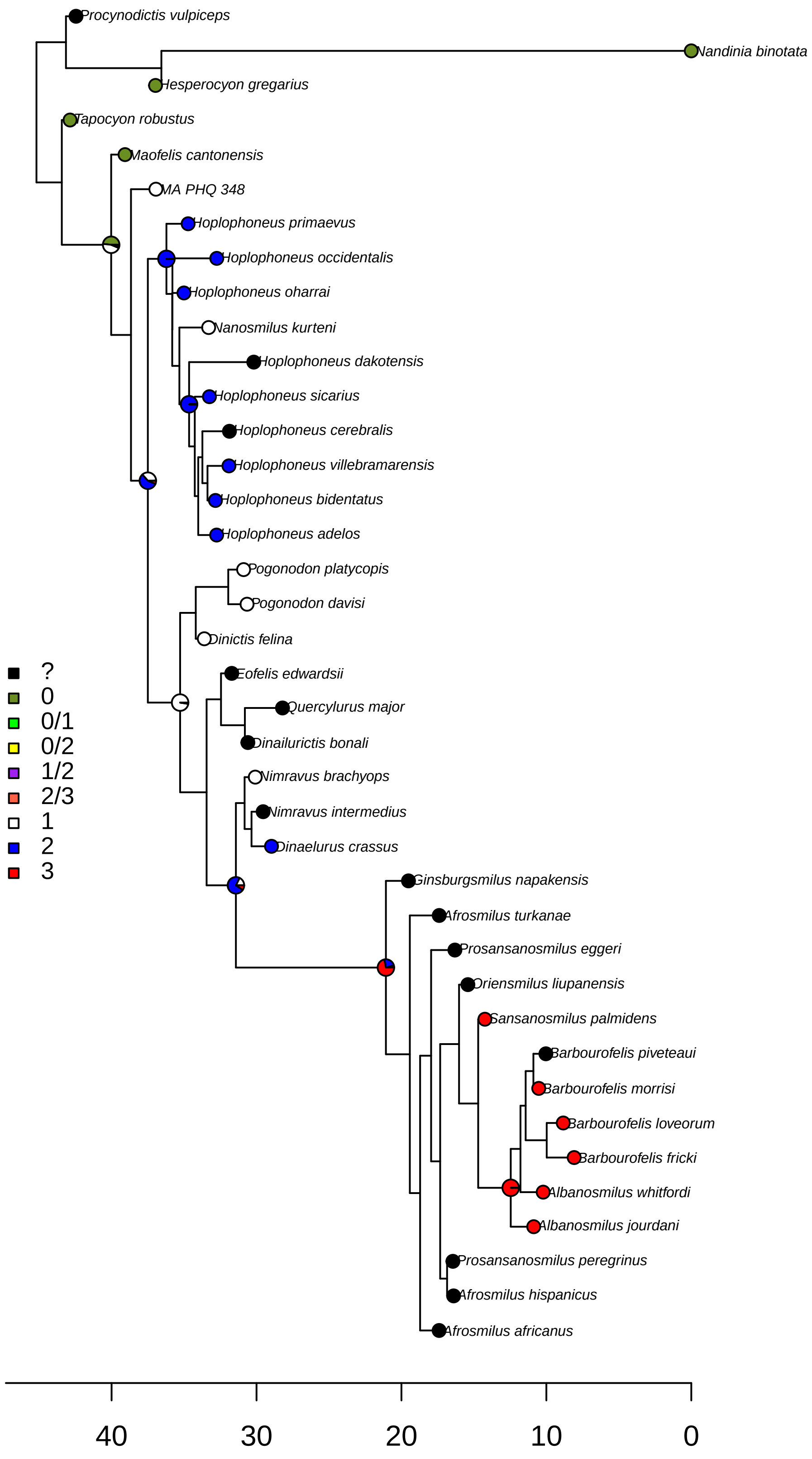

Synapomorphy 214

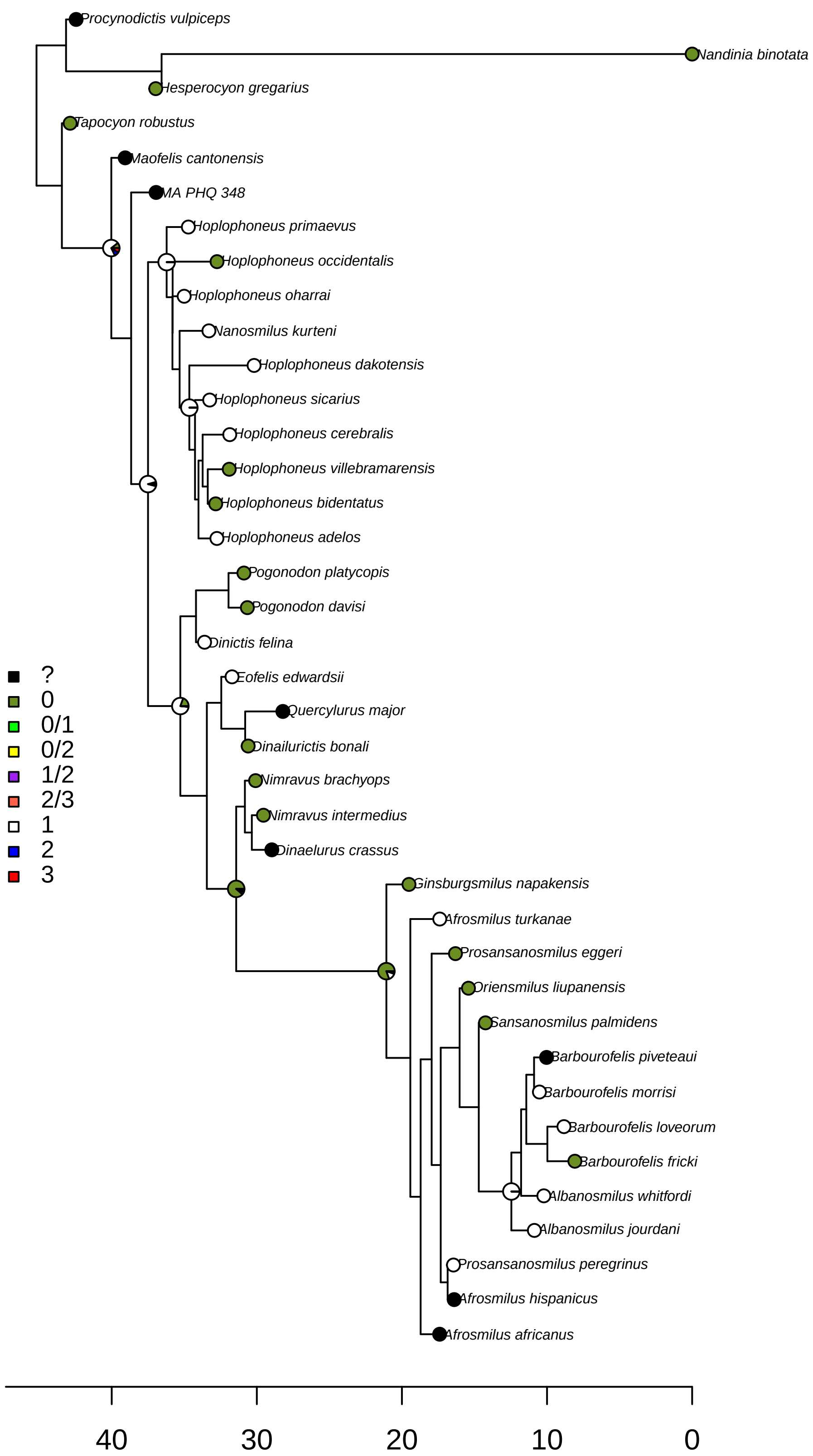

Synapomorphy 219

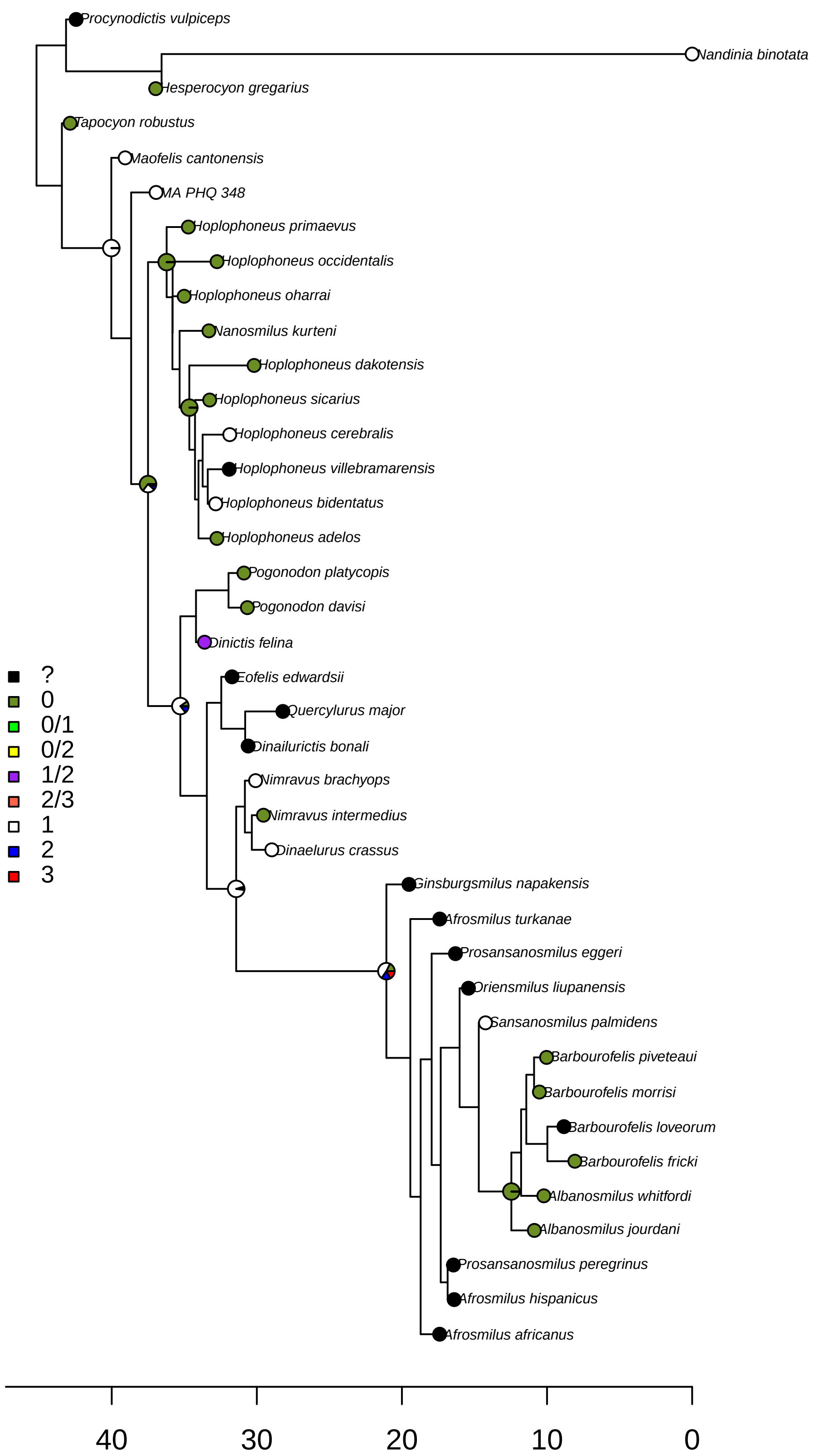

Synapomorphy 223

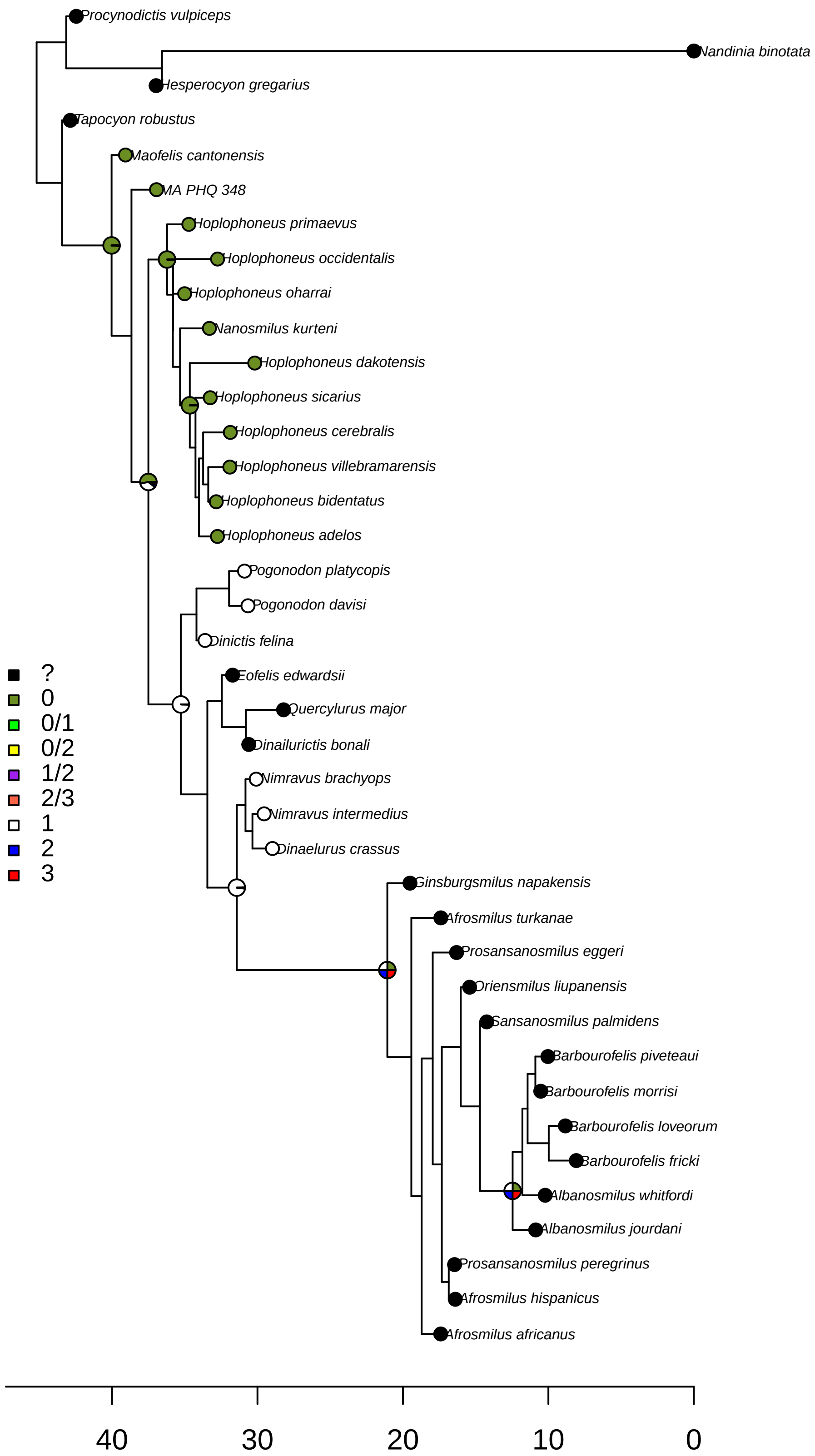

Supplement: Supplementary file 2 — Supplementary Information 2. [file 41598_2021_521_MOESM2_ESM.zip › Synapomorphies.pdf]
