# Supplementary material for: The largest hoplophonine and a complex new hypothesis of nimravid evolution
Source: Sci Rep. 2021 Oct 26;11:21078. doi: 10.1038/s41598-021-00521-1 (PMC8548586; doi:10.1038/s41598-021-00521-1)
Supplement: Supplementary file 3 — Supplementary Information 3. [file 41598_2021_521_MOESM3_ESM.docx]

**Supplementary Material**

**The largest hoplophonine and a complex new hypothesis of nimravid evolution**

Paul Zachary Barrett^1*^

^1^Department of Earth Sciences, University of Oregon, Eugene, Oregon 97403, U.S.A.

^*^Corresponding Author:

Email address: pbarrett@uoregon.edu

**Contents:**

*Eusmilus adelos* Detailed Description

Supplementary Table 1: Source Material for Character Scoring and Continuous Variables

Morphological Character List

Stratigraphic Ranges of Analyzed Taxa

Custom Evolutionary Models (Rate Matrices)

Synapomorphies of the Nimravidae and Select Internal Clades

Figures S1-S3. *Eusmilus adelos* specimen figures

Figure S4. Maximum clade credibility tree of the best-supported Bayesian analysis

Figure S5. Maximum clade credibility tree of analysis with no *Ginsburgsmilus* dentary or postcranial material

Legends for Datasets S1 to S2

SI References

**Supplementary Information Datasets (uploaded to Figshare):**

Dataset S1: BEAST2 xml run files of all partitioning and evolutionary model schemes (.zip) and morphology character matrix (.nex)

Dataset S2: BayesTraits run files and synapomorphy output (.zip)

***Eusmilus adelos* Detailed Description**

*Cranium*

A nimravid of large size with basilar lengths of 221 mm and 233 mm, USNM 18214 and 12820 respectively, compare to *H. primaevus* with a mean value of 154mm^1^. The rostral portion of the cranium is approximately 62-65% of the condylobasal length (Figs. 1; 2C; S1C-D, G-H), while the braincase is the remainder.

In lateral view, the dorsal margin of the cranium shifts from convex to concave (anterior to posterior), with its highest point at the inion. The apex of the nasals are visible, being an anterior projection in an otherwise convex rostrum. The premaxillary dental arcade exhibits a significant amount of prognathism, though typical of other derived dirk-toothed carnivores. The infraorbital foramen is large and applied closely to the antero-ventral corner of the orbit, above the center of the P3. The postorbital process of the frontal is hooked and ventral projecting. There is a small lacrimal process. The zygomatic arch, tall and thin, curves gently dorsally before plunging ventrally (anterior to posterior) as it meets the glenoid pedicle. The jugal does not contact the lacrimal, while it does with the glenoid pedicle which possesses anterior and posterior lips of the glenoid socket which project equally ventrally. The large mastoid processes, with simple paroccipital process, lie closely behind, oriented antero-ventrally. The lamboid crest, and entire occiput, is rotated anteriorly, so that the angle of the crest is approximately 90 degrees relative to the cingular border of the cheektooth row, compare to a mean value of 116 for *Hoplophoneus primaevus*^1^. The occipital condyles and foramen magnum are thus posteriorly oriented, displaying an axial alignment with the dorsal portion of the nasal aperture.

In dorsal view (Figs. 2A; S1A,E), the rostrum is convex about the large canine alveolus transitioning to concave prior to the zygomatic arch. The arch itself is triangular, like all species of *Eusmilus*, having its widest measurement at the most posterior portion, the glenoid pedicle. The large mastoid processes are visible, as are the occipital condyles, with the lamboid crest lying (near vertically oriented) in between. The nasal suture is sunken, likely related to some taphonomic compression. The nasals are shortened, such that the posterior margin does not extend beyond the maxillofrontal suture, a situation that otherwise only exists in the “toy sabertooth” *Eusmilus cerebralis* and Late Miocene *Barbourofelis fricki*. The temporal lines are well defined and terminate slightly anterior of the coronal suture which in turn is located approximately at the postorbital constriction.

The palate (Figs. 1B; S1B,F) is triangular, with the posterior width greater than the width at the canines. The premaxillary-maxillary suture of the palate occurs approximately 30% along the length of the canine (anterior to posterior) and is adjacent to the incisive foramen which is long and narrow. The midline length of the maxilla is greater than that of the palatine, which terminates posteriorly at the level of the P3 main cusp. The anterior opening of the palatine canal is through the maxilla, approximately halfway between the canine and P3. Distinct grooves run anteriorly from these foramina converging just medial of the canines. The posterior edge of the palate midline is aligned with the posterior edge of the P4. Though not preserved in USNM 12820, a midline posterior projection of the palatine exists in USNM 18214. The posterior palatine foramen is not present on the ventral surface of the palate, a conspicuous feature of the holotype of *Eusmilus sicarius* (YPM PU 12953). The choanae are 25-30% larger than the foramen magnum, while the pterygoid processes converge posteriorly towards the basicranium, terminating just anterior of the alisphenoid canal.

The basicranium region is relatively well preserved. However, there are no bullar elements attached to either cranium. The foramen ovale and posterior opening of the alisphenoid canal reside in a common groove that opens ventrally and is located anteromedially of the glenoid fossa. USNM 12820 displays two small foramina posterior to the left glenoid process, likely supernumerary postglenoid foramina, the right side contains only one small foramen. USNM 18214 preserves only one small postglenoid foramen on its left side. There is substantial lateral buttressing of the basisphenoid and basisoccipital which project ventrally beyond the occipital condyles, suggestive that the bullar elements were strongly attached in life. There is no median ridge between these buttresses, but the sutural contact between the basisphenoid and basioccipital splits them about equally, just anterior of the promontorium. The petrosal is only substantially preserved in USNM 12820. It is placed in a deep depression just medial of the mastoid process, though dorsal to the ventral surfaces of all surrounding basicranial elements (basisphenoid, basioccipital, squamosal, mastoid). The promontorium is low and rounded with remains of a small posterior platform. There is no preserved portion indicative of a ventral process of the promontorium. USNM 12820 preserves a lateral notch suggestive of the location of the stylomastoid foramen. The fenestra rotunda is placed centrally. The roof and medial wall of the petrobasilar canal for the inferior petrosal venous sinus is formed entirely by the basioccipital, whereas the petrosal contacts the basioccipital and contributes to the lateral wall of the canal for its preserved portion. The paroccipital process is simple and short, exhibiting a sutural contact with the mastoid process that travels into the auditory region. The petrobasilar foramen is aligned with this suture on the medial side of the auditory capsule and confluent with the petrobasilar canal. A large hypoglossal foramen is placed posterior to the former foramen, though separated by the basioccipital, within a groove between the occipital condyle and paroccipital process.

In posterior view the nuchal crest is near vertical and spade-shaped with rugosities along its external edge for muscle insertion. The foramen magnum is circular and slightly extends dorsally beyond the occipital condyles. The lateral buttressing of the basioccipitals is visible and extends ventrally, approximately a centimeter, beyond the occipital condyles. The mastoid processes are large and hooked-shaped projecting ventrally within a centimeter of the ventral border of the glenoid fossa. The paroccipital processes are large, approximately the same size of the foramen magnum, but simple rugose swellings.

*Dentition*

The incisors are closely appressed in a pronounced arch that extends anteriorly more than a centimeter beyond the anterior edge of the canines. Only two incisors are preserved amongst the referred specimens. However, the alveoli of the incisor arch display substantial anterior projection of these teeth which additionally show they were transversally compressed and became progressively larger, I1 to I3. USNM 18214 possesses the root of the right I3 which is semicircular in cross-section, compressed flat along its mesial edge while curved along is lateral one. The tooth is 5.70 mm in width and anteriorly projecting. The crown of a caniniform I1 or I2 is preserved disarticulated with USNM 12820. This tooth is recurved, 12.23 mm in crown height, 3.62 mm in width and 6.36 mm in length. There is a small diastema, approximately 7.5-10 mm, between I3 and C1.

The following dental descriptions come from USNM 12820. USNM 18214 has either badly damaged or missing teeth which precludes much descriptive information.

The canines (length 31.90 mm, 33.20 mm; width 6.80 mm, 8.36 mm; preserved height 18.35 mm, 62.45 mm; measured at the maxillary border for the left and right tooth respectively) are massive and dagger-like. The canines are substantially compressed with a ratio (length/width) of 3.97 to 4.69, compare to *E. sicarius* with 6.58, and *E. dakotensis* with 2.25 ^1^. These teeth are deciduous with a medial furrow, through which the adult canine teeth can be seen erupting on both sides. The adult canines display fine serrations on both the anterior and posterior edges, while none are preserved on the juvenile canines. The serration density per millimeter (averaged over 5mm) on the posterior edge of the adult canine is 5.58, compare to *H. primaevus* with a mean value of 4.4 ^1^.

What is interpreted to be a tiny (length 3.82mm) P2 is present on the left side of USNM 12820 (Fig. 2B), about halfway between the canine and P3. Otherwise no teeth are present in this region, resulting in a long postcanine diastema of approximately 32mm on USNM 18214 and a variable 20.66 to 25.35mm (not counting the P2) on USNM 12820. The discrepancy of length in values arises from an oddly placed P3 on the right side. This tooth is smaller than that of the left side, but more anteriorly placed and displays a small gap between it and the P4 behind.

The P3 (Fig. 3) is double-rooted (length 11.04 mm, 10.72 mm; width 5.77 mm, 5.39 mm; height 8.94 mm, 8.94 mm, for the left and right tooth respectively measured at the base of the cingulum). The principle cusp is large and conical on its labial side, while flattened on the lingual portion. The principal cusp is separated from the posterior blade-like cusp by a small notch. On the left side this blade is indented to form an additional small notch, composed of the distinct ventrally projecting cingular ridge for the posterior-most portion. There are remnants of fine serrations found along the occlusal edge. There is no anterior cusp.

The P4 (Fig. 3) is typical of carnivoran carnassials (length 21.66 mm, 21.30 mm; width at the protoconal root 10.21 mm, 10.69 mm, width at the carnassial notch 8.60 mm, 8.23 mm; height 16.66 mm, 16.03 mm for the left and right tooth respectively). The main cusp is a large triangular paracone, separated by a large notch from the metastylar blade behind. A distinct rounded “parastyle” exists anterior of the paracone. Interestingly, an anterior blade-like projection of the paracone ascends dorsally from the tip of the paracone to meet the parastyle, forming an anterior notch between these cusps. There is no protocone, though the root that would normally support this cusp is still present, located slightly anterior of the paracone which it is smoothly connected to. Like the P3, a large labial cingular ridge is present on the posterior portion of this tooth.

The M1 (Fig. 3) is reduced like other members of the *Eusmilus* genus (length 6.96 mm, 7.16 mm; width 12.55 mm, 13.59 mm; height 7.51 mm, 8.61 mm, left and right tooth respectively). The anterolabial corner of the tooth features a hooked parastyle that reaches out and is applied against the adjacent P4. From the parastyle, the distinct and raised paracrista travels lingually to the raised paracone, before turning posterior (as the centrocrista) to the posterior termination of the tooth. These cristae feature fine serrations along their length. Lingual of the paracone, the tooth smoothly slopes off to the root of what would be the protocone. However, no cusp is present in this area. Like most of the taxa of the Hoplophoninae, this tooth exhibits substantial vertical rotation, such that the centrocrista is located in a more horizontal plane than a vertical one.

*Dentary*

Partial right and left dentaries are preserved for USNM 12820 (Fig. 2D). The area of preservation extends from the anterior edge of the p4 alveolus to the anterior portion of the masseteric fossa for the right dentary. While the left’s preservation also starts at the anterior portion of the p4 alveolus, it terminates at the posterior portion of the m1. Judging from the p4 alveoli, this tooth would have been large (length 19.39 mm; width 6.51 mm, measurements for right side). Given the orientation of the roots, this tooth in life would have also presented significant lateral rotation, a condition noted for numerous nimravid taxa ^2^. The p4s lie in an imbricated arrangement to the following m1s, with p4s lateral to them. The m1s preserve portions of their roots (length 23.72 mm, greatest width 8.01 mm, measurements for the right side). The anterior root makes up approximately 60% of the tooth and has a slight medial orientation (posterior to anterior), while the posterior root is more medially placed and tapers to the rear. Given the preservation it is impossible to determine the presence of a p3 in life. The depth of the dentary below the m1 is substantial (28.42 mm, 26.39 mm, left and right respectively), compare to 30.8 mm for E*. sicarius* and 32.67 mm for *E. dakotensis*.

*Postcrania*

USNM 12820 preserves several portions of its postcrania (Figs. 1; S2-3), while USNM 18214 has none preserved. Thus, the following descriptions apply solely to it.

*Vertebrae*

Five cervical vertebrae (atlas, axis, 3^rd^, 5^th^ and 7^th^) are preserved in good condition. The transverse processes of the atlas are mostly missing, but it can be inferred that the alar groove was not roofed over to form a foramen. The axis has a tall and posteriorly-projecting neural spine, extending well beyond the posterior zygopophyses. The remaining cervical vertebrae feature no unusual morphology, though small accessory processes between the neural process and posterior zygopophyses are located on what is inferred to be C7, structures which presence is also noted for *Hoplophoneus* and *Nimravus* ^3,4^.

Three partial lumbar vertebrae (two of which are articulated) represent the remainder of the preserved axial skeleton. As in most Carnivora, the pre- and postzygopophyses have a strong interlocking nature which prevents movement in the transverse plane. The neural spine occupies the entire length of the neural arch, though breakage prevents estimates of height and orientation. The transverse processes are also broken, but based on preserved remnants, would have been robust and anteriorly projecting.

*Scapula*

Much of the left scapula is preserved, though most of the lateral half remains embedded in matrix. The area of the supraspinous fossa to the infraspinous fossa is approximately equivalent. The scapular spine is narrow anteroposteriorly as compared to other species of *Hoplophoneus* and *Dinictis*^3^. The area of the glenoid and acromion is poorly preserved, so details of anatomy cannot be inferred. Likewise, proximoposterior corner of the scapula lacks preservation so inference to the presence of the teres major process cannot be made.

*Humerus*

Approximately half of the left humerus is preserved, from midshaft to the distal end. The pectoral ridge is high and transversally compressed, terminating just proximal of the supinator crest. The supinator crest is large, flaring out laterally, while closer to the distal end it curves anteriorly. Most of the medial epicondyle is not preserved, but it can be inferred from the bony strut that would have been large and enclosed a entepicondylar foramen. The ulnar trochlea is shallow and oriented slightly anteromedially to posterolaterally. In anterior view a shallow notch exists on the proximal portion of the capitulum, while the head of the capitulum is flat in distal view, like that of *Dinictis felina*, compared to the rounded heads of other *Hoplophoneus* species. The olecranon fossa is transversely oblong and shallow.

*Radius*

Most of the left radius is preserved, including the complete proximal end, while the distal is missing its epiphysis. The element is oblong in cross-section, with greater width in the mediolateral direction that increases towards the distal end. The head and neck of the proximal end curve slightly medially. The head is oval and shallowly concave. A small notch exists on the anterior edge of the articular surface, positioned laterally to a prominent ridge that runs anteroposteriorly. The radial tuberosity is approximately 1.5cm in length, crescent shaped, and positioned about one centimeter below the radial head. The preserved distal end is smooth anteriorly while a medial projecting ridge is present on the posterior side.

*Ulna*

The proximal left ulna is preserved, save a portion of the olecranon process. The ulna is robust and has a substantial lateral concave curve. The semilunar notch is concave and primarily faces laterally. The distal border of the semilunar notch is smooth like other species of *Hoplophoneus*, compared to an indentation present in *Dinictis felina*. The radial notch is flat, being more similar to *Dincitis* and *Nimravus*, than other species of *Hoplophoneus* which exhibit a curved radial notch.

Supplementary Table 1

Source Material for Morphological Character Scoring and Continuous Variables

American Museum of Natural History, New York City, New York, USA (**AMNH**)

American Museum of Natural History – Frick Collection, New York City, New York, USA (**F:AM**)

American Museum of Natural History, Comparative Anatomy Collection, New York City, New York, USA (**AMNH C.A.**)

Carnegie Museum of National History, Pittsburgh, USA (**CMNH**)

Field Museum of Natural History, Chicago, Illinois, USA (**FMNH**)

Smithsonian National Museum of Natural History, Washington D.C., USA (**USNM**)

John Day Fossil Beds National Monument, Kimberly, Oregon, USA (**JODA**)

University of Nebraska Lincoln State Museum, Lincoln, Nebraska, USA (**UNSM**)

Florida Museum of Natural History, University of Florida, Gainesville, Florida, USA (**UF**)

University of California Museum of Paleontology, Berkeley, California, USA (**UCMP**)

Yale Peabody Museum, Princeton University Collection, New Haven, Connecticut, USA (**YPM PU**)

University of Kansas, Natural History Museum, Lawrence, Kansas, USA (**KUVP**)

Raymond M. Alf Museum of Paleontology, Claremont, California, USA (**RAM**)

Museum of Geology, South Dakota School of Mines and Technology, Rapid City, South Dakota, USA (**SDSM**)

Museum für Naturkunde, Berlin, Germany (**MFN MB. Ma.**)

Beijing Natural History Museum, Beijing, China (**BNHM**)

Collection du Quercy, Faculté des Sciences de Marseille, France (**FSM PQ**)

University of Washington Burke Museum, Seattle, Washington, USA (**UWBM**)

Kenya National Museum, Nairobi, Kenya (**KNM**)

Uganda Museum, Kampala, Uganda (**UM**)

| **Taxa** | **Specimens** | **Literature** |
| --- | --- | --- |
| ***Barbourofelis morrisi*** | AMNH 79999; F:AM 61876, 61900, 80000, 61895, 25204, 69359, 61893, 61976, 61898, 61970, 125665, 61882, Bx-5-Bx-69 #25138-B | - |
| ***Oriensmilus liupanensis*** | AMNH 144755 (cast of an uncatalogued BNHM specimen) | ^5^ |
| ***Sansanosmilus palmidens*** | - | ^6–9^ |
| ***Barbourofelis fricki*** | AMNH 108193 (cast of UNSM 76000); F:AM 61986, 61994, 61997, 99258, 68234, 2672, 61984, 61983, 125670, 116854, 61991; UWBM 72291 | ^10,11^ |
| ***Barbourofelis loveorum*** | UF 24447, 36855, 36871, 37000, 36867, 23796, 24432, 36867, 25081, 25103, 25156, 25191, 25228, 25249, 25267, 25283, 25294, 25302, 27258, 37939, 25054, 25034, 36800, 25013, 24429; AMNH 125125 | ^3,12–16^ |
| ***Albanosmilus whitfordi*** | AMNH 14308; F:AM 61856, 61858, 61861, 61864, 61849, 61680, 61885 | - |
| ***Eusmilus cerebralis*** | AMNH 6941; F:AM 98189, 69377; UCMP 123180, 123181; JODA 7047 | - |
| ***Hoplophoneus oharrai*** | SDSM 2417 | - |
| ***Hoplophoneus primaevus*** | F:AM 62007, AMNH 5338, 82440, 38980; USNM 18184; YPM PU 10741 | ^1,3^ |
| ***Hoplophoneus occidentalis*** | F:AM 102387, 62025, 62022; AMNH 1407, 655; KUVP 2874, 2561; RAM 10356 | - |
| ***Nimravus brachyops*** | UCMP 1681, 2556, 76111; AMNH 6930, 6993, 6940, 6933; F:AM 62020, 62151; JODA 1312 | ^1,17^ |
| ***Nimravus intermedius*** | AMNH 137130 (cast of FSM PQ 327), 105390 (cast of CMNH 2587); MFN MB. Ma. 29987, 29988, 29986, 29991, 29993, 29994, 29995; YPM PU 11569 | ^18–21^ |
| ***Pogonodon platycopis*** | AMNH 6938, 6953 | ^1^ |
| ***Pogonodon davisi*** | UCMP 789; AMNH 102156; F:AM 62026, 62042, 62024, 62018, YPM 10520; YPM PU 11430 | - |
| ***Dinictis felina*** | AMNH 6937, 8777, 38805; YPM PU 12551, 13625, 12577, 11431 | - |
| ***Nanosmilus kurteni*** | UNSM 25505 | - |
| ***Ginsburgsmilus napakensis*** | KNM-SO-5670; UM-P67-13 | ^9,22–24^ |
| ***Prosansanosmilus eggeri*** | - | ^9^ |
| ***Prosansanosmilus peregrinus*** | - | ^9,25,26^ |
| ***Afrosmilus hispanicus*** | - | ^22,27,28^ |
| ***Afrosmilus turkanae*** | KNM-MO-15929, KNM-RU-15984, KNM-RU-15986, KNM-SO15973 | ^22,23,29^ |
| ***Afrosmilus africanus*** | - | ^22,30,31^ |
| ***Albanosmilus jourdani*** | - | ^32^ |
| ***Maofelis cantonensis*** | - | ^33^ |
| ***Quercylurus major*** | - | ^21,34^ |
| ***Dinailurictis bonali*** | MFN MB. Ma. 29985 | ^21,34^ |
| ***Eofelis edwardsii*** | - | ^34–36^ |
| ***Dinaelurus crassus*** | - | ^37^ |
| **MA-PHQ 348** | - | ^38^ |
| ***Eusmilus dakotensis*** | YPM PU 11079; UNSM 1068 | ^39–41^ |
| ***Eusmilus sicarius*** | YPM PU 12953 A (cast of YPM PU 12953) | - |
| ***Eusmilus adelos*** | USNM 12820, 18214 | - |
| ***Eusmilus bidentatus*** | MFN MB. Ma. 30154, 30151 | ^42–45^ |
| ***Eusmilus villebramarensis*** | - | ^21,43^ |
| ***Barbourofelis piveteaui*** | - | ^46^ |
| ***Nandinia binotata*** | AMNH 2409 C.A.; FMNH 149361, 55758 | ^47–49^ |
| ***Tapocyon robustus*** | - | ^47,48,50^ |
| ***Procynodictis vulpiceps*** | AMNH 2514 | ^47,48^ |
| ***Hesperocyon gregarius*** | AMNH 50276 | ^47,48,51–53^ |

Morphological Character List

1. Lacrimal facial process: (0) small, present on face; (1) not present on face; (2) orbital

flange reduced to area around lacrimal foramen ^47: character 1^.

2. Length of palate – position of the posterior edge of palatine midline relative to tooth row: (0) posterior to upper tooth row; (1) anterior or equal to upper tooth row ^47: character 5^.

3. Palatine canal primary anterior opening: (0), at maxilla–palatine suture; (1) opening through maxilla ^47: character 6^.

4. Relative length of frontal and parietal at midline: (0) parietal greater than frontal; (1) parietal equal or subequal to frontal; (2) frontal midline much longer than parietal ^47: character 7^.

5. Postorbital process: (0) prominent; (1) small, reduced ^47: character 8^.

6. Paroccipital process size: (0) well-developed; (1) reduced ^47: character 9^.

7. Paroccipital process shape: (0) simple process; (1) laterally flattened, thin, but is distinct process; (2) absent ^47: character 10^.

8. Postglenoid foramen: (0) present; (1) greatly reduced, or missing ^47: character 12^.

9. Shape of mastoid process: (0) forming a distinct process, extending out farther than paroccipital process, or subequal; (1) blunt, rounded, does not protrude significantly, more a swelling of the mastoid; (2) large tabular process, anteriorly sloping, obscuring much, if not all, of the auditory bulla ^modified from 47: character 13^.

10. Condyloid (hypoglossal) foramen position relative to postlacerate foramen: (0) distant; (1) close (less than the diameter of the hypoglossal foramen away); (2) conjoined with posterior lacerate foramen ^47: character 15^.

11. Condyloid (hypoglossal) foramen position relative to groove between the occipital condyle and the paroccipital process: (0) inline or within groove; (1) anterior to groove ^47: character 16^.

12. Fenestra cochleae (rotunda) position relative to mastoid tubercle: (0) posterior to mastoid tubercle; (1) anterior, subequal to mastoid tubercle ^47: character 18^.

13. Relative distance between the foramen ovale and the alisphenoid canal: (0) separated by at least the diameter of the alisphenoid canal; (1) separated only by a thin wall ^47: character 19^.

14. Ossification of tegmen tympani: (0) facial nerve exposed ventrally; (1) facial nerve beneath a bony sheath that defines the fossa for tensor tympani muscle ^47: character 20^.

15. Anterior loop of internal carotid artery: (0) lack of an anterior loop of the internal carotid artery; (1) presence of the loop – excavation in basisphenoid ^47: character 23^.

16. Suprameatal fossa (fossa on squamosal anterior to mastoid: (0) absent; (1) small ^47: character 24^.

17. Position of internal carotid artery: (0) internal carotid artery laterally positioned, transpromontorial, runs close to margin of fenestra cochlea, presence of a promontory artery, groove for stapedial artery may or may not be present; (1) internal carotid artery transpromontorial but medially positioned, course far from fenestra cochlea; (2) internal carotid artery medial, extrabullar, inside a bony canal formed by the caudal entotympanic ^47: character 25^.

18. Apron shelf on promontorium posterior to fenestra cochleae for entotympanic attachment: (0) absent; (1) blunt – surface present posterior to fenestra cochleae, but no extensive attachment possible; (2) extended, large area for attachment, may roof posterior bullar chamber ^47: character 26^.

19. Ventral process of promontorium: (0) absent; (1) present, medially positioned on promontorium ^47: character 27^.

20. Shape of the promontorium, anterior extension: (0) elongate, apron extension tapers to a point anteriorly, almondlike in appearance; (1) elongate, rounded anteriorly; (2) blunt, quickly truncating; (3) elongate, apron is broad, flat extension, not almond-shaped and not blunt ^47: character 28^.

21. Inferior petrosal sinus: (0) inferior petrosal sinus small; (1) inferior petrosal sinus greatly enlarged ^47: character 31^.

22. Shelf between mastoid process and paroccipital process: (0) laterally wide, curved trough with smooth surface; (1) laterally wide, could have flat surface, rugose or bulbous, no smoothed out trough ^47: character 33^.

23. Extent of flange on basiocciptal lateral edge bordering auditory region: (0) absent; (1) small, nascent; (2) well developed when compared to basal ‘miacids’ ^47: character 34^.

24. Evidence on basisphenoid and basioccipital for marked medial inflation of the entotympanic: (0) absent; (1) present – inflation of entotympanic pushing medially onto and over the basioccipital ^47: character 35^.

25. Evidence of marked posterior inflation of the entotympanic; entotympanic attached during life to paroccipital process or to extensive area posterior to the petrosal: (0) absent; (1) present ^47: character 36^.

26. Epitympanic wing of the petrosal forms ventral floor to the anterior medial corner of the fossa for the tensor tympani muscle (Fig. 9): (0) absent; (1) present, but relatively flat and horizontal ^47: character 38^.

27. Placement of middle lacerate foramen (Fig. 8): (Ordered) (0) foramen a vacuity – not defined anteriorly nor posteriorly, positioned directly anterior to petrosal; (1) foramen anteriorly defined, posteriorly bordered by petrosal – positioned equal or posterior to basisphenoid/basioccipital suture; (2) foramen defined anteriorly, petrosal may be undefined posterior border, foramen positioned in basisphenoid (or edge of alisphenoid) just anterior to basisphenoid/basioccipital suture; (3) foramen defined anteriorly and posteriorly completely bordered by basisphenoid, foramen positioned far anterior to basisphenoid/basioccipital suture ^47: character 40^.

28. M1 a defined cingulum continuous around the lingual face of the protocone: (0) absent; (1) complete cingulum present; (2) anterior segment of cingulae absent or smaller than posterior cingulae ^47: character 41^.

29. M1 protocone height relative to paracone: (0) protocone shorter than paracone; (1) protocone equal or subequal to height of paracone; (2) protocone absent or lacks a cusp associated with the root ^modified from 47: character 42^.

30. M1 parastyle direction: (0) buccally with anterior direction; (1) buccally ^47: character 45^.

31. M1 size of posterior lingual cingular shelf at base of protocone: (0) posterior lingual cingular shelf equal or subequal to anterior cingulum; (1) posterior lingual cingular shelf more pronounced, larger than anterior cingulum ^47: character 47^.

32. M1 relative height of paracone and metacone: (0) paracone equals metacone in height; (1) paracone greater than metacone; (2) metacone absent ^modified from 47: character 48^.

33. M1 relative height of paraconule and metaconule: (0) paraconule equal or subequal to metaconule; (1) both absent ^47: character 49^.

34. M1 width of parastylar shelf: (0) lack of a shelf; (1) broad; (2) narrow, consisting mainly of ridge ^47: character 51^.

35. Presence of M3: (0) present; (1) absent ^47: character 53^.

36. P4 protocone: (0) large, well-developed; (1) reduced or absent ^47: character 56^.

37. Posterior accessory cusps on P3: (0) one cusp present; (1) two cusps present ^47: character 58^.

38. Palatine, relative size: (0) midline length of palatine less than midline length of maxilla; (1) midline length greater than midline length of maxilla ^47: character 60^.

39. Jugal: (0) jugal reaches lacrimal, or is separated from it by only thin sliver of maxilla; (1) jugal widely separated from lacrimal, maxilla broadly laps posteriorly over anterior orbital rim ^47: character 64^.

40. Postorbital constriction: (0) just anterior of frontoparietal suture, near posterior margin of frontal; (1) braincase expanded, with frontals making much greater contribution; fronto–parietal suture located more anteriorly in frontal ^47: character 66^.

41. Posterior entrance of carotid artery into auditory capsule: (0) posterior entry, artery not enclosed in osseous tube; (1) posterior entry, artery enclosed in tube; (2) anterior entry, artery not enclosed in tube ^47: character 67^.

42. Entotympanic: (0) fails to ossify, or is only weakly attached to auditory capsule; (1) ossified at least partially, and firmly fused to the skull ^47: character 68^.

43. Entotympanic septum: (0) absent; (1) present ^47: character 71^.

44. P1: (0) present; (1) absent ^47: character 79^.

45. p1: (0) present; (1) absent ^47: character 84^.

46. m3: (0) present; (1) absent ^47: character 88^.

47. Scapula, postscapular fossa: (0) absent; (1) present ^47: character 90^.

48. Femur, third trocanter: (0) present; (1) absent ^47: character 94^.

49. Scapula – supraglenoid tubercle morphology: (0) expands out over the glenoid fossa (*Didymictis*); (1) blunt, does not extend over the glenoid fossa (*Vulpavus*) ^48: character 100^.

50. Scapula – acromion process angle: Either a short or a long process can be dorsally directed at the acromion process apex, or remain in the same plane as the scapular spine: (0) process angles dorsally (*Vulpavus*); (1) process remains in same plane as the scapular spine (*Canis*) ^48: character 101^.

51. Scapula – acromion process length: The acromion process can terminate far distally to the glenoid fossa or terminate at or before the border. A long acromion can be used as a proxy for the presence of clavicles in extinct taxa. (0) process extends past glenoid fossa (*Vulpavus*); 1– process terminates before or at glenoid fossa (*Gulo*) ^48: character 102^.

52. Scapula – coracoid process: (0) present, as a large clearly projecting process (*Felis*); (1) extremely small or absent (*Canis*) ^48: character 103^.

53. Humerus – olecranon fossa shape: (0) shallow and round (*Vulpavus*); (1) deep and slot-like (*Miacis uintensis*); (2) perforated (*Didymictis*) ^48: character 107^.

54. Humerus – medial edge of posterior trochlea: (0) vertical (*Vulpavus*); (1) slanted (*Öodectes*) ^48: character 108^.

55. Humerus – delto-pectoral crest: (0) present (*Vulpavus*); (1) absent (*Didymictis*) ^48: character 109^.

56. Humerus – medial epicondyle: (0) ends with a well rounded head (*Vulpavus*); (1)

poorly defined, appearing more like a blunt tubercle (*Didymictis*) ^48: character 110^.

57. Humerus – greater tuberosity height: (0) extends past head (*Didymictis*); (1) flush with head in height (*Vulpavus*) ^48: character 112^.

58. Humerus – brachial flange: (0) present and large, extending out from the body of the bone as a flat surface (*Vulpavus*); (1) small, nothing but a small raised line of bone (*Didymictis*) ^48: character 114^.

59. Humerus – lesser tuberosity with a crest or ridge of bone leading from this feature down the shaft: (0) present (*Didymictis*); (1) absent (*Canis*) ^48: character 115^.

60. Humerus – trochlea extent: (0) extends distally past capitulum when viewed anteriorly (*Didymictis*); (1) two articular surfaces are more inline (*Vulpavus*) ^48: character 116^.

61. Humerus – epicondylar foramen: (0) present and round (*Hyaenodon*); (1) present and elongated (*Vulpavus*) ^48: character 117^.

62. Humerus – distal L-shaped ridge of bone on capitulum in distal view (new): (0) present (*Vulpavus*) (1) absent (*Felis*) ^48: character 118^.

63. Humerus – ulnar collateral ligament insertion site size: (0) very large, forming a distinct circular pit (*Didymictis*); (1) small, forming only a shallow depression (*Vulpavus*) ^48: character 120^.

64. Humerus – greater tuberosity angle: (0) greater tuberosity angled away from head; smooth arch is not formed with lesser tuberosity (*Didymictis*); (1) greater tuberosity more flush with head (*Vulpavus*) ^48: character 121^.

65. Humerus – prominence of bicipital groove: (0) groove is very noticeable and deep (*Nandinia*); (1) groove is very subtle if noticeable at all (*Civetticits*) ^48: character 122^.

66. Humerus – capitulum shape: (0) flat for the whole length with a uniform distal margin (*Thinocyon*); (1) rounded (*Vulpavus*) ^48: character 123^.

67. Humerus - tricipital line: (0) large and distinctive (*Didymictis*); (1) reduced (*Nandinia*) ^48: character 124^.

68. Ulna – semi-lunar notch distal border morphology: (0) W-shaped distal border; an indentation occurs between the articular surface with the radius and the rest of the facet (*Öodectes*); (1) indentation lacking (*Canis*) ^48: character 125^.

69. Ulna – semi-lunar notch proximal border extent: (0) proximal ridge extends far from shaft surface (*Hyeanodon*); (1) proximal ridge flush with shaft surface (*Öodectes*) ^48: character 126^.

70. Ulna – *m. bracialis* insertion site: (0) present (*Vulpavus*); (1) absent (*Ailurus*) ^48: character 127^.

71. Ulna – *m. bracialis* insertion site position: (0) on anterior surface of ulna (*Vulpavus*); (1) on the medial margin of the shaft (*Öodectes*) ^48: character 128^.

72. Ulna – radial notch curvature: (0) absent (*Vulpavus*); (1) present (*Ailurus*) ^48: character 129^.

73. Ulna – deep tendon groove on proximal end of ulna: (0) present (*Hyaenodon*); (1)

absent (*Vulpavus*) ^48: character 130^.

74. Ulna – olecranon process shape: (0) mediolaterally robust, square in shape (*Vulpavus*); (1) mediolaterally compressed (*Felis*) ^48: character 132^.

75. Ulna – anconeal process extent: (0) projecting from the shaft, shelf like (*Didymictis*); (1) flush with shaft (*Vulpavus*) ^48: character 133^.

76. Ulna – proximal border of semi-lunar notch, position: (0) lateral to the shaft (*Hyaenodon*); (1) centered with respect to the shaft (*Öodectes*) ^48: character 134^.

77. Ulna – groove on lateral side of shaft: (0) present (*Hyaenodon*); (1) absent (*Vulpavus*) ^48: character 136^.

78. Ulna shape – sigmoidal; the ulna is used as a proxy for the curved nature of all forelimb bones noted by Matthew : (0) sigmoidal (*Vulpavus*); (1) straight (*Canis*) ^48: character 137^.

79. Radius – radial head shape: (0) round (*Vulpavus*); (1) oval (*Didymictis*) ^48: character 139^.

80. Radius – large scaphoid articulation surface: (0) present (*Didymictis*); (1) surface small

(*Vulpavus*) ^48: character 141^.

81. Radius – capitular eminence of radial head development; (0) small (*Vulpavus*); (1) large, disrupts radial rim (*Canis*) ^48: character 142^.

82. Carpus – cuneiform shape in proximal view: (0) triangular (*Nandinia*); (1) rectangular (*Vulpavus*) ^48: character 144^.

83. Carpus – proximal surface of scapholunar, curvature: (0) fully convex (*Nandinia*); (1) convex and concave (*Herpestes*) ^48: character 145^.

84. Carpus – uniciform width (new): (0) as wide as trapezoid and magnum combined (*Canis*); (1) small, only as wide as magnum alone (*Nandinia*) ^48: character 146^.

85. Carpus – cuneiform articulation with ulna, position: (0) on medial (radial) margin (*Felis*); (1) on distal surface of ulna (*Ursus*) ^48: character 150^.

86. Carpus –metacarpal II strongly overlaps III proximally: (0) overlap small or absent (*Canis*); (1) overlap present and substantial (*Felis*) ^48: character 151^.

87. Carpus – metacarpal length: (0) metacarpal lengths are equivalent to sum of phalanges (*Vulpavus*); 1– phalanges are longer than metacarpals (Otariidae) ^48: character 152^.

88. Carpus – medial phalanx distal articular surface symmetry: (0) symmetrical (*Vulpavus*); (1) asymmetrical (*Felis*) ^48: character 153^.

89. Carpus – proximal phalanges compressed dorsoventrally: (0) absent (*Vulpavus*); (1) present (*Hoplophoneus*) ^48: character 154^.

90. Carpus – lateral excavation of the medial phalanx; (0) absent (*Vulpavus*); (1) present (*Felis*) ^48: character 155^.

91. Femur – lesser trochanter orientation: (0) projects posteriorly (*Didymictis*); (1) projects medially (*Vulpavus*) ^48: character 156^.

92. Femur – intertrochanteric crest extent: (0) extends to lesser trochanter (*Didymictis*); (1) becomes flush with shaft before reaching the lesser trochanter (*Vulpavus*) ^48: character 157^.

93. Femur – position of lesser trochanter relative to the third trochanter: (0) third trochanter is lower (*Vulpavus*); (1) third trochanter is roughly at the same level (*Didymictis*) ^48: character 158^.

94. Femur – supracondylar tuberosities: (0) absent (*Vulpavus*); (1) presence of two or more raised tuberosities just proximal to the condyles of the femur on the posterior shaft (*Canis*) ^48: character 160^.

95. Femur – height of greater trochanter relative to head of femur: (0) greater trochanter is higher or sub-equal (*Vulpavus*); (1) greater trochanter is lower than head of femur (*Ursus*) ^48: character 161^.

96. Femur – medial condyle morphology: (0) proximal edge of the articular surface of condyles are flush with the shaft, due to the lack of development of a condylar neck (*Vulpavus*); (1) proximal edge of the articular surface of condyles are not flush with the shaft, due to the development of a condylar neck (*Canis*) ^48: character 162^.

97. Femur – lesser trochanter development: (0) strong, prominently projecting feature (*Vulpavus*); (1) small, barely more than a small bump or ridge on the shaft (*Ursus*) ^48: character 163^.

98. Femur – greater trochanter broadest surface, orientation: (0) faces laterally (*Vulpavus*); (1) faces posteriorly (*Canis*) ^48: character 164^.

99. Femur – patellar groove shape: (0) deep and narrow (*Didymictis*); (1) wide and flat (*Vulpavus*) ^48: character 166^.

100. Femur – position of greater trochanter relative to shaft: (0) greater trochanter over distal midline (*Erinaceus*); (1) greater trochanter lateral to midline (*Didymictis*) ^48: character 167^.

101. Tibia – posterior bone spur on distal tibia: (0) presence of a prominent bone spur on the posterior margin (*Vulpavus*); (1) spur absent (*Nandinia*) ^48: character 170^.

102. Tibia – contact of inner borders of condyles: (0) in contact (*Vulpavus*); (1) separate (*Canis*) ^48: character 171^.

103. Pes – astragalus – height of fibular facet: (0) height subequal to length, resulting in a roughly square shaped facet (*Vulpavus*); (1) height much less than length, resulting in a more crescent shaped feature (*Canis*) ^48: character 175^.

104. Pes – astragalar head shape when viewed dorsally: (0) rounded (*Vulpavus*); (1) flattened (*Canis*) ^48: character 176^.

105. Pes – height of astragalar head when viewed distally: (0) apex of astragalar head very low, does not rise above astragalar neck (head flattened) (*Vulpavus*); (1) apex of astragalar head rises in between astragalar neck and articular surface of astragalar body (*Canis*) ^48: character 177^.

106. Pes – astragalus fossa, on the lateral edge, posterior ventral quadrant: (0) present (*Didymictis*); (1) absent (*Vulpavus*) ^48: character 178^.

107. Pes – astragalus lateral margin, anterior ventral edge expansion: (0) lateral expansion present (*Vulpavus*); (1) expansion absent, creating a much smoother margin (*Procyon*) ^48: character 179^.

108. Pes – astragalar head medial articulation surface extent (new): (0) extends backwards a great distance, past the border of the sustentacular facet (*Hyaenodon*); (1) short, does not extend past the start of the neck (*Vulpavus*) ^48: character 180^.

109. Pes – astragalus sustentacular facet shape: (0) has clear edges, is somewhat convex, and does not extend into gully (*Vulpavus*); (1) is more flat than convex and extends into gully (*Canis*) ^48: character 181^.

110. Pes – astragalar foramen; (0) present and dorsally positioned (*Vulpavus*); (1) present and posteriorly positioned (*Hyaenodon*); (2) absent (*Canis*) ^48: character 182^.

111. Pes – astragalus, posterior ridge for ligament attachments, presence: (0) present, high (*Vulpavus*); (1) low or absent (*Felis*) ^48: character 183^.

112. Pes – astragalus, orientation of posterior ridge for ligament attachments: (0) orientated at an oblique angle relative to the long axis of the astragalar trochlea (*Vulpavus*); (1) orientated in line with the trochlea (*Canis*) ^48: character 184^.

113. Pes – astragalus-tibia articulation surface, extent: (0) covers entire posterior dorsal surface (*Canis*); (1) restricted, fails to cover entire posterior dorsal surface, leaving a gap in the lateral posterior quadrant (*Vulpavus*) ^48: character 185^.

114. Pes – astragalus, cotylar fossa presence: (0) present (*Didymictis*); (1) absent (*Vulpavus*) ^48: character 187^.

115. Pes – Calcaneus, peroneal tubercle development: (0) weakly developed, little more than a ridge (*Didymictis*); (1) well developed (*Vulpavus*) ^48: character 189^.

116. Pes – calcaneus, cuboid facet shape: (0) irregularly shaped (*Didymictis*); (1) round (*Vulpavus*) ^48: character 191^.

117. Pes – calcaneus, cuboid facet orientation: (0) angled dorsally (*Vulpavus*); (1) in planes other than dorsal (*Didymictis*) ^48: character 192^.

118. Pes – calcaneus –dorsal facet morphology: (0) smooth (*Vulpavus*); (1) clearly defined, sharp corner (rather than a smooth curve) (*Canis*) ^48: character 194^.

119. Pes – cuboid shape: (0) relatively rectangular (*Didymictis*); (1) wider proximally than

distally (*Vulpavus*) ^48: character 196^.

120. Pelvis – ilium, anterior expansion: (0) not expanded (*Felis*); (1) expanded dorsoventrally (*Canis*) Primitively for Carnivoramorpha the anterior ilium is not dorsoventrally expanded; however in *Canis*, *Ursus*, *Hyaena*, and *Civettictis* a clear expansion is seen ^48: character 200^.

121. Pelvis – ischial spine position: (0) located just posterior of the border of the acetabulum (*Didymictis*); (1) far posterior from acetabulum (*Vulpavus*); (2) spine absent (*Erinaceus*) ^48: character 201^.

122. Pelvis – ilium, anterior region morphology: (0) broad and flat (*Canis*); (1) divided by a distinct ridge running antero-posteriorly (*Vulpavus*) ^48: character 204^.

123. Pelvis – ilium, ventral surface morphology: (0) broad and flat (*Thinocyon*); (1) narrow (*Vulpavus*) ^48: character 205^.

124. Atlas – alar foramen: (0) absent, only a notch is present (*Canis*); (1) present, confluent with lateral vertebral foramen (*Herpestes*); (2) present, separate from lateral vertebral foramen (*Procyon*) ^48: character 207^.

125. Atlas – transverse processes, orientation: (0) projects at right angle from the body (*Canis*); (1) extends posteriorly at an acute angle to the body (*Nandinia*) ^48: character 208^.

126. Axis – body length: (0) roughly as long as it is wide (*Vulpavus*); (1) elongated relative to its width (*Felis*) ^48: character 210^.

127. Cervical vertebrae – keel: (0) large ventral keel present (*Nandinia*); (1) ventral keel absent (*Dinictis*) ^48: character 211^.

128. Cervical vertebrae – spinous processes, size: (0) large (*Canis*); (1) small (*Felis*) ^48: character 212^.

129. Sacrum – size compared to pelvis: (0) small, does not reach border of acetabulum (*Canis*); (1) large, reaches border of acetabulum (*Felis*) ^48: character 215^.

130. Alisphenoid, foramen ovale (position): (0) anterior to glenoid fossa; (1) medial to glenoid fossa. Comparisons made to the posterior-most edge of the glenoid fossa, not the process. Serves for the passage of the trigeminal nerve (V), internal maxillary artery and the medial meningeal artery ^modified from 54: character 71^.

131. Entotympanic, septum: (0) anterior/posterior orientation; (1) medial/lateral orientation ^modified from 54: character 98^.

132. Entotympanic, ventral floor ossification: (0) ossified; (1) unossified (i.e. *Nandinia*) ^modified from 54: character 102^.

133. Vagina processus hyoideus: (0) confluent with stylomastoid foramen (giving the appearance of one foramen); (1) slightly separate from stylomastoid foramen ^54: character 129^.

134. i1- i3 projection: (0) extends upward; (1) procumbent (less than 45 degrees) ^54: character 151^.

135. P3 number or roots: (0) 2 roots; (1) 1 root; (2) 3 roots ^54: character 164^.

136. P4 posterolingual cingulum: (0) present; (1) vestigial; (2) absent ^54: character 177^.

137. p4 talonid: (0) absent/vestigial; (1) present ^54: character 178^.

138. p4 posterior accessory cusps: (0) 2 cusps, linear; (1) 1 cusp; (2) no cusps; (3) 2 or more cusps, nonlinear ^54: character 179^.

139. P4-M1 carnassial embrasure pit: (0) present; (1) absent ^54: character 181^.

140. M1 roots: (0) 3 roots; (1) 2 roots; (2) 1 root ^54: character 183^.

141. M1 hypocone: (0) present; (1) absent ^54: character 184^.

142. m1 trigonid (trgd) proportion of m1. (0): trigonid length is 60% to 73% of total length; (1): 77-87%; (2): 88% and higher; (3): no talonid ^1: character 30^.

143. m1 metaconid: (0) large; (1) considerably smaller than paraconid; (2) absent ^54: character 189^.

144. m1 entoconid: (0) present; (1) absent ^54: character 191^.

145. m1 hypoconid: (0) present; (1) absent ^54: character 192^.

146. M2 roots: (0) 3 roots; (1) 2 roots; (2) tooth absent ^modified from 54: character 198^.

147. m2 cusps: (0) talonid not elongate, tooth oval outline, no enlarged hypoconulid; (1) low trigonid, well developed talonid cusps; (2) tooth with cusps but no clear trigonid or talonid; (3) tooth without clearly defined cusps; (4) absent ^modified from 54: character 202^.

148. p4 anterior accessory cusp: (0) absent; (1) smaller than posterior; (2) about equal to posterior; (3) larger than posterior ^54: character 221^.

149. Volume contribution of the feloid auditory bulla: (0) both chambers meet over the petrosal and display an external ‘infolding’ at their juncture with a vertical septum (e.g. viverrids, herpestids); (1) anterior chamber formed by rostral entotympanic or pseudoseptum.

150. Mastoid invasion by bulla: (0) absent; (1) present.

151. P4 nimravid “parastyle”: (0) absent; (1) present ^55: character 222^.

152. P2: (0) double-rooted; (1) single-rooted; (2) absent ^55: character 224^.

153. p3 posterior cusps: (0) no cusps; (1) one cusp (posterior cingular cusp); (2) two cusps (posterior cingular cusp and posterior accessory cusp) ^55: character 229^.

154. Basicranial foramina: (0) petrobasilar venous sinus and sigmoid sinus merge intracranially and exit the posterior lacerate (jugular) foramen; (1) veins merge extracranially, passing through the petrobasilar and posterior lacerate foramina respectively ^55: character 231^.

155. P4 protocone: (0) medial or posterior to paracone; (1) anterior to paracone or paracone-parastyle juncture; (2) anterior face of protocone approximately level with anterior face of parastyle ^modified from 56: character 9^.

156. Shape of dentary tooth row: (0), tooth row straight; (l), tooth row curved, or convex ^56: character 9^.

157. P4 metacone/metastyle blade length to paracone length ratio: (0) 0.6-0.99; (1) 1-1.3; (2) 1.4 and greater ^modified from 56: character 11^.

158. Placement of carnassials in tooth row: (0) carnassials in line with tooth row, i.e. at an angle relative to the sagittal plane); (l) carnassials parallel to sagittal plane ^56: character 14^.

159. Placement of anterior accessory cusp of p4: (0) free of main cusp; (l) appressed to main cusp ^56: character 14^.

160. Shape of anterior face of p3: (0) concave/straight; (l) convex ^56: character 15^.

161. Position of infraorbital foramen: (0) positioned anterior to middle of P3; (1) positioned above middle to posterior of P3; (2) positioned above anterior edge of P4 ^modified from 56: character 22^.

162. Position of anterior margin of orbit Coding: (0) above or behind metastylar blade of P4; (1) above the anterior end of P4; (2) above P3 ^modified from 56: character 23^.

163. Suture between premaxillary and frontal on snout coding: (0) absent; (l) present ^56: character 24^.

164. Size of inferior oblique muscle fossa at maxillary-lacrimal suture postero-dorsal to infra-orbital foramen: (0) small; (l) large; (2) absent ^modified from 56: character 25^.

165. Sphenoid foramen and postpalatine foramen position: (0) well separated, distinct foramina; (l) foramina located close together in a single depression ^56: character 27^.

166. The contribution of the maxilla to the antero-internal rim of the zygomatic arch coding: (0) small to none; (l) maxillary makes up a substantial portion of the antero-dorsal margin of the zygomatic arch ^56: character 28^.

167. Shape of basioccipital in ventral view: (0) flat; (l) lateral ridges and central groove ^56: character 32^.

168. Position of premaxillary-maxillary suture on palate: (0) near the middle of the incisive fossa; (l) at the postero-lateral margin of the incisive fossa ^56: character 33^.

169. Shape of jugal-maxillary suture in external view Coding. – (0) angled downwards posteriorly in a straight or weakly sinusoid manner; (1) horizontally straight ^modified from 56: character 36^.

170. Position of external auditory meatus: (0) far forwards of nuchal crest; (l) level with nuchal crest) ^56: character 43^.

171. Shape of nuchal crest: (0) antero-posteriorly inclined; (1) nearly vertical or vertical ^56: character 44^.

172. Size of metacarpal 1: (0) metacarpal 1 has a long, slender diaphysis resembling the other four metacarpals; (1) metacarpal 1 has a blunt and rectangular shape (e.g. felids); (2) vestigial ^modified from 56: character 45^.

173. Relative width of p3 (ranked in order by ratio of Wp3 to Lp3): (0) 0.2-0.39; (1) 0.4-0.6; (2) 0.61-0.66; (3) 0.69 and greater ^57: character 12^.

174. Relative width of p4 (ranked in order by ratio of Wp4 to Lp4): (0) 0.38 and less; (1) 0.40-0.54; (2) 0.55-0.68; (3) 0.72 and greater ^57: character 13^.

175. Relative width of P3 (ranked in order by ratio of WP3 to LP3): (0) 0.38 and less; (1) 0.4-0.65; (2) 0.66-0.79; (3) 0.81 and above ^57: character 14^.

176. Labiolingual compression of the C1: (0) very slightly compressed (length/breadth index smaller than 1.5); (1) markedly compressed (index between 1.5 and 1.8); (2) very compressed (index 1.81-3.0); (3) extremely compressed 4.0 and greater ^32: character 3^.

177. C1 vertical grooves: (0) absent; (1) present ^32: character 4^.

178. P3 anterior cingulum cusp: (0) absent; (1) present ^32: character 7^.

179. P4 preparastyle: (0) absent; (1) present ^32: character 10^.

180. p2: (0) present; (1) absent ^32: character 16^.

181. Orbit: (0) open; (1) closed ^32: character 23^.

182. Position of M1. Polarity: (0) distal to P4; (1) medial to P4 ^32: character 27^.

183. c1: (0) larger than i3; (1) same size ^32: character 31^.

184. Ratio P4 length /p4 length: (0) 1.0-1.87; (1) 1.95-2.1; (2) 2.2 and higher ^32: character 32^.

185. Masseteric fossa on the lateral surface of the maxilla and jugal: (0) shallow or absent; (1) deep with distinct dorsal margin ^1: character 3^.

186. Fossa on the medial face of the zygomatic arch, below the postorbital process: (0) no fossa; (1) presence of a marked fossa ^21: character 12^.

187. Zygomata shape in dorsal view: (0) broadly circular; (1) triangular ^1: character 5^.

188. Shape of the glenoid socket of the craniomandibular joint: (0) anterior lip is missing; (1) posterior lip of the glenoid socket projects more ventrally than anterior lip; (2) anterior lip and posterior lip project equally ventrally ^1: character 10^.

189. Anterior mandible position: (0) in line with the tooth row, mandibular border of cheek

tooth row is in the same plane as the mandibular border of the incisors and canines; (1) elevated

above tooth row; (2) cheek teeth and anterior teeth brought again into same plane by elevation of cheek teeth on pedestal ^1: character 13^.

190. Development and orientation of the coronoid process of the dentary: (0) posteriorly

orientated posterior border without surpassing the level of the mandibular condyle; (1) surpassing the level of the mandibular condyle; (2) vertically orientated posterior border; (3) anteriorly orientated posterior border ^1: character 20^.

191. Size of the genial flange of the dentary in adult taxa. Measured as the height of the genial flange from the anterior portion of the postcanine diastema to the ventral apex of the genial flange/length of dentary from the posterior articular surface to the most anterior aspect: (0) no flange, the ventral rim of the chin is regularly curved; (1) no flange, but the ventral rim of the chin is distinctly angulate; (2) short flange, between 22 and 31% of the total length of mandible; (3) deep flange, 32-50% of the total length of the mandible; (4) extremely deep flange, 54% or more of the total mandibular length ^1: character 15^.

192. Mental fossa. Fossa on the ventral-internal face of the chin. (0): no fossa; (1): fossa present and marked ^1: character 16^.

193. Incisors: (0) spatulate; (1) mostly spatulate, I3 caniform; (2) all caniform ^1: character 17^.

194. C1 length. Mesial-distal length of C1 measured at the dentine/enamel boundary: (0) less than that of P4; (1) greater than that of P4 ^1: character 18^.

195. Serration density of permanent upper canines per millimeter. Measured over an average of 5 mm: (0) none; (1) 0.7-2.7; (2) 2.8-5.0; (3) ≥ 5.5 ^modified from 1: character 19^.

196. Size of P3 vs. size of P4. Measured as a ratio of crown height (base of cingulum to apex of tooth) on adult minimally worn teeth: (0) 0.71-1.1; (1) 0.48-0.70; (2) 0.45 and lower ^modified from 1: character 20^.

197. p3 crown height compared to p4: (0) 0.6-0.98; (1) 1.0 and greater; (2) less than 0.58; (3) p3 absent ^modified from 1: character 26^.

198. Cheek tooth serrations. Serrations on adult minimally worn cheek teeth: (0) absent; (1) present ^1: character 31^.

199. Articulation between the calcaneum and navicular : (0) absent; (1) present ^1: character 33^.

200. Anterior palatine canal opening location: (0) level with P3; (1) anterior to P3; (2) P4 ^modified from 39: character 4^.

201. Position of nasals relative to maxilla-frontal suture: (0) nasals extend beyond the maxillofrontal suture; (1) nasals shortened, the posterior border lies across or anterior to the maxillofrontal suture ^21: character 9^.

202. P4, orientation of the protocone: (0) anterolingually projected; (1) lingually projected ^21: character 23^.

203. Dorsal part of frontoparietal (coronal) suture: (0) smooth, lacking a parietal process; (1) suture irregular with a distinct, often very large, medio-dorsally directed parietal process ^58: character 13^.

204. Dorsal Jugal-squamosal suture in zygomatic arch: (0) abuts the postorbital process; (1) does not abut the postorbital process ^modified from 58: character 19^.

205. Paroccipital process: (0) ventrally directed; (1) posteriorly directed ^modified from 58: character 22^.

206. Angle of long axis of bulla to long axis of skull: (0) Less than or equal to 19°; (1) 20°-27°; (2) 28°-37° ^modified from 58: character 29^.

207. Upper incisor arcade: (0) strongly parabolic; (1) slightly parabolic such that the anterior edge of I3 does not contact a straight edge held across the anterior of the arcade; (2) straight ^59: character 1^.

208. P4 anterior cingulum: (0) indistinct; (1) distinct and often forming a ridge ^modified from 59: character 11^.

209. Relative height of the m1 major cusps, measured from tip of cusp to base of cingulum: (0) height of paraconid low compared with height of protoconid (65–93%); (1) height of paraconid tall compared with height of protoconid (95–130%) ^modified from 59: character 24^.

210. Relative length of p4: (0) small relative to mandible length (~7.5–9%); (1) large relative to mandible length (10–15%) ^modified from 59: character 26^.

211. Size of the jugal postorbital process: (0) small, rounded, often almost absent; (1) tall, triangular and tapering ^59: character 39^.

212. Relative width across the upper incisor arcade compared to cranial basilar length (CBL): (0) 11% and less; (1) narrow (12–15% of CBL); (2) wide (16–19% of CBL); (3) extremely wide (21–25% of CBL) ^modified from 59: character 42^.

213. Palatal region relative width: (0) 16-22%; (1) palate across the center of P3 comparatively narrow compared with CBL (23–25%); (2) palatal region across center of P3 relatively wide (27–34% of CBL); (3) palatal region across center of P3 extremely wide (36–43% of CBL) ^modified from 59: character 43^.

214. Mandibular fossa termination: (0) posterior to the carnassial; (1) terminating below talonid or before carnassial notch ^modified from 59: character 49^.

215. Lower canines, buccal vertical groove/ridge: (0) present; (1) absent ^modified from 60: character 22^.

216. C1 lingual ridge: (0) absent; (1) present ^61: character 11^.

217. Stylomastoid groove. (0) groove originating from the stylomastoid foramen; (1) groove originating from the tympanohyal pit ^62: character 41^.

218. Metatarsal 1: (0) functional metatarsal, articulating with a phalanx; (1) vestigial metatarsal 1 ^63: character 20^.

219. Orientation of premaxillary-maxillary suture relative to upper tooth row: (0) 52°-70°; (1) moderately steep, 70°-80°; (2) very steep, greater than 80° ^modified from 64: character 3^.

220. Teres major fossa: (0) absent; (1) long ^modified from 64: character 20^.

221. Scapholunar, articular surface for trapezoid: (0) convex and concave; (1) concave only ^64: character 37^.

222. m1 anterolingul cingulum: (0) absent; (1) present.

223. Petrobasilar foramen: (0) open vacuity, (1) closed foramen in basioccipital.

224. Anterior cingular cusp of p3: (0) present; (1) absent.

225. Crenulations on the anterior face of the paracone of P4 and occlusal face of the protocone: (0) absent; (1) present.

**Stratigraphic Ranges of Analyzed Taxa**

*Caniformia*

*Hesperocyon gregarious*. **Locality**: Numerous across North America, see Wang ^51^. **Absolute age estimate**: 41.4-29.75 Ma. A relatively ubiquitous taxon of the Chadronian through Whitneyan, the earliest records extend into possibly the earliest Duchesnean ^51^. These earliest specimens come from the Lac Pelletier Lower Fauna of Saskatchewan, but precise age constraints for this fauna is still debated ^53^. Thus, the age range chosen for this taxon was the entirety of the Duchesnean through Whitneyan ^65^, as defined by Kelly et al. ^66^ pending more precise dating.

*Procynodictis vulpiceps*. **Locality**: Wyoming, USA. **Absolute age estimate**: 45.15-41.4 Ma. The stratigraphic range of this taxon appears to encompass the entire Uintan ^67^, though the latest occurrence is in some doubt ^68,69^. For this study the entire Uintan NALMA was used, as defined by Kelly et al. ^66^.

*Feliformia*

*Tapocyon robustus*. **Locality**: California, Montana, Wyoming, Utah, USA. **Absolute age estimate**: 46.5 - 39.7 Ma ^67^. *Tapocyon* has typically been placed as the immediate sister taxon to crown Carnivora based on parsimony analysis of morphological data ^47^. However, Barrett et al. ^55^ recovered it as a stem feliform, as did Tomiya and Tseng ^70^ and thus forms an outgroup for this study.

*Nandiniidae*

*Nandinia binotata*. **Locality**: Central and Western Africa. **Absolutue age estimate**: 0 Ma. With a very deep molecular divergence estimate ^71,72^, the sole extant nandiniid has a frustratingly incomplete fossil record. Though, Morales et al. ^73^ described *Nandinia sp.*, from the Late Miocene Lukeino Formation, Kenya (6.1-5.7 Ma). However, the fragmentary nature of this specimen precludes its inclusion in this analysis and would likely not impact divergence time estimates.

*Nimravidae*

*Oriensmilus liupanensis*. **Locality***:* Tongxin area of Ningxia Hui Autonomous Region, northern China^5^. **Absolute age estimate**: 16.0-15.0 Ma ^74^. The holotype and additionally referred material is from an informal middle member of the Zhang’enbao Formation, from which emanates the Ma’erzhuizi Gou Fauna ^75^, that the authors included within the Dingjia’ergou Fauna. The latter fauna is the date to which I applied the age estimate of this taxon, as did Wang et al. ^5^.

*Sansanosmilus palmidens.* **Locality***:* France. **Absolute age estimate**: 16.0-13.0 Ma. The majority of *S. palmidens* material comes from southern France at the locality of Sansan, MN 6 ^8^. However, additional fragmentary material has been described from Savigné-sur-Lathan (MN5) of western France ^76^.

*Barbourofelis fricki* **Locality***:* Kansas, Nebraska, Texas, Nevada, USA. **Absolute age estimate:** 9.0–7.0 Ma. ^77^

*Barbourofelis loveorum* **Locality***:* Florida, USA. **Absolute age estimate:** 9.5–8.0 Ma. ^77^

*Barbourofelis morrisi* **Locality***:* Nevada, South Dakota, Texas, USA. **Absolute age estimate:** 12.0–9.5 Ma. ^77^

*Albanosmilus whitfordi* **Locality***:* California, Colorado, Nebraska, Texas, Florida, USA. **Absolute age estimate:** 12.0–7.0 Ma. ^77^

*Barbourofelis piveteaui,* **Locality***:* Sinap, Ankara; Turkey. **Absolute age estimate**: 10.899-9.279 Ma. The holotype specimen has no precise locality formation, but is referred to the Middle Sinap Formation ^46^. An additional, more complete specimen, is referred to Sinap Locality 2 of the Middle Sinap Member of the Sinap Formation ^46^. Thus, the age range used here follows the findings of Kappelman et al. ^78^ and Koufos et al. ^79^ for the entire middle member of the Sinap Formation.

*Albanosmilus jourdani* **Locality**: Spain, France, Turkey. **Absolute age estimate**:11.9-9.7 Ma. ^32^.

*Eusmilus cerebralis.* **Locality***:* South Dakota, California, Oregon, Wyoming, USA. **Absolute age estimate**: 34.7-29.586 Ma. The earliest specimen referable to this taxon is an edentulous dentary from the Crazy Johnson Member of the Chadron Formation ^1^. The latest well-documented occurrence is from below the “Blue Basin Tuff” of the Turtle Cove Member of the John Day Formation, 29.586 Ma ^80^.

*Hoplophoneus primaevus.* **Locality***:* Nebraska, South Dakota, Wyoming, Colorado, Oregon, USA. **Absolute age estimate**: 35.7-30.58 Ma. Following the taxonomy of Barrett^1^, the oldest occurrence of this taxon is from Flagstaff Rim Wyoming ^39,81^. The youngest specimens attributed to this taxon are found just below the “Upper Whitney Ash” in Nebraska and the basal *Leptauchenia* beds of the Poleslide Member of the Brule in South Dakota ^39,81^.

*Hoplophoneus occidentalis.* **Locality***:* Nebraska, North Dakota, South Dakota, Wyoming, USA. **Absolute age estimate**: 33.4-31.4 Ma. The earliest stratigraphically constrained specimens of this taxon come from the “lower nodules” of the Scenic Member of the Brule Formation, correlated to 33.4-33.1 Ma ^39,81^. The latest occurring specimens emanate from the *Protoceros* channel sandstones of the Poleslide Member of the Brule Formation ^1,39^.

*Nimravus brachyops*. **Locality***:* Nebraska, South Dakota, California, Oregon, Wyoming (USA); Saskatchewan (Canada)*.* **Absolute age estimate**: 31.4- 27.14 Ma. This is a geographically wide-ranging taxon in North America with its first occurrence in the *Protoceros* channel sandstones of the Poleslide Member of the Brule Formation, South Dakota ^39,81^. The last reported occurrence of this taxon is from the K1 unit of the Turtle Cove Member of the John Day Formation ^80^. The K1 unit is capped by the “Biotite Tuff” and forms the LAD for this study.

*Pogonodon platycopis.* **Locality***:* South Dakota, Nebraska, Oregon, USA. **Absolute age estimate**: 32.0-29.586 Ma. Numerous specimens of Whitneyan age are referred to this taxon, with none being clearly older ^1,39^. The youngest age comes from Unit E of the Turtle Cove Member of the John Day Formation, below the “Blue Basin Tuff” ^80^.

*Pogonodon davisi.* **Locality***:* Wyoming, Nebraska, South Dakota, Oregon, USA. **Absolute age estimate**: 32.0-25.9 Ma. Like *P. platycopis*, the earliest record of this taxon is Whitneyan in age ^1,39^. The youngest specimen (JODA 5841) attributable to this taxon comes from the K2 unit of the Turtle Cove Member of the John Day Formation. This specimen was referred to as a possible new species of *Pogonodon* by Bryant and Fremd ^82^ and Albright et al. ^83^, but is included in this taxon following the diagnosis of Barrett ^1^.

*Dinictis felina*. **Locality***:* South Dakota, North Dakota, Nebraska, Oregon, Montana, Wyoming (USA); Saskatchewan (Canada). **Absolute age estimate**: 35.7- 29.586 Ma. The oldest record known is at Flagstaff Rim, at just over 35.5 Ma, though several slightly older occurrences are possible from fragmentary remains and provisional dates ^39^. For example, *Dinictis sp.* has been collected from the Medicine Poles local fauna of southwestern North Dakota, thought to be Ch2 in age ^84^. This latter occurrence is used as the FAD of this taxon. The LAD includes the occurrence of *Dinictis cyclops= D. felina* ^1^ below the Blue Basin Tuff, 29.586 Ma ^80^.

*Nanosmilus kurteni.* **Locality***:* Nebraska, USA. **Absolute age estimate**: 33.89-32.0 Ma. The holotype and only known specimen is from the Orella Member of the Brule Formation^85^. Absolute radiometrically dated boundaries for the Orella Member are not known, but bracketed to 34.6-31.5 Ma^86^ based on stratigraphically and geographically, nearest dated ash layers. However, dates for the Orellan NALMA are better known and what is used for the range applied to this taxon^81,86^.

*Nimravus intermedius.* **Locality***:* France, Germany, Mongolia. **Absolute age estimate**: 35.37-27.24 Ma. Numerous fragmentary remains are known from the old Quercy collections, as well as the new stratigraphically documented ones, MP 22 to 25 ^21,87^. Additional French material comes from Villebramar, MP 22 ^21^. Of similar age in Southern Germany are attributed dentition at Möhren 13 (MP 22) and Liptingen, MP 22 or MP 23 ^21^. Mongolian *Nimravus* material, of Ergilian age, was separated into its own species, *Nimravus mongoliensis*, for much of the 20^th^ century ^88,89^, but was synonymized to *N. intermedius* by Peigné ^21^. Most recently this material was re-established as a valid species by Egi et al. ^18^ based upon depth of the horizontal ramus and relative size of p1 and p2. However, the variability in anterior premolars for *Nimravus* has been well documented in the literature ^1,21,90^. Thus, until more complete material of this purported species is described I chose to follow the assement of Peigné ^21^ in its synonymy. The Thai, ‘*Nimravus’* material described by Peigné et al. ^91^ lacks the apomorphy of p4 morphology for this genus, and may be more closely related to *Maofelis* for which no lower dentognathic remains are known.

*Ginsburgsmilus napakensis.* **Locality***:* Langental, Fiskus, Grillental-6, Sperrgebiet, Namibia; Songhor, Kenya; Napak, Uganda. **Absolute age estimate**: 20.0-19.0 Ma. An updated occurrence list can be found in Morales and Pickford ^23^, while Werdelin ^92^ describes all of the above sites to be correlative to 20-19 Ma.

*Prosansanosmilus eggeri.* **Locality***:* Bavaria, Germany. **Absolute age estimate**: 16.7-15.97 Ma.

Thus far, the only known specimens of this taxon come from Sandelzhausen (MN 5) of Bavaria ^9^. Sandelzhausen is constrained in age to about 16 Ma based on magnetochronological events and biostratigraphy; see Moser et al. ^93^ for a detailed overview.

*Prosansanosmilus peregrinus.* **Locality***:* Germany, France. **Absolute age estimate**: 18.0-16.0 Ma. In their original description, Heizmann et al. ^25^ named three localities for the occurrence of this taxon: Langenau 1 of Germany; and Bézian and Artenay of France. To this list, Petersbuch 2 of Germany was added by Morlo^26^. Petersbuch 2 and Artenay are MN 4a in age while Langenau 1 and Bézian are MN 4b. The age of MN 4 is skewed slightly older in the depositional basins of Switzerland and Southern Germany ^94^ than the European “standard” (e.g. ^95^). Furthermore, the exact age of the above fossil localities within their respective subzones is unequally known, save with Langenau 1 at slightly less than 17.2 Ma ^94^. Thus conservatively, the base of the South German MN 4a was used as the FAD of this taxon, while the LAD the “standard” top of MN 4.

*Afrosmilus africanus.* **Locality***:* Locherangan, Rusinga, and Karungu (Kenya); Buñol, Spain. **Absolute age estimate**: 17.8-16.0 Ma. *A. africanus* is known from the African sites of Locherangan, Rusinga, and Karungu (Kenya), as well as the Spanish locality of Buñol. The Kenyan locality Locherangan, c. 17.5 Ma ^96^ is located west of Lake Turkana, while Rusinga, c. 17.8 Ma ^92^ and Karungu, c. 17.7-17.5 Ma ^97^, though likely contemporaneous with Rusinga ^92^ or slightly older ^23^ based on biostratigraphy, are localities along the northwestern shore of Lake Victoria. The Spanish locality of Buñol, MN4, 16.6-16.0 Ma ^95^ is located in the east of Spain in the province of Valencia.

*Afrosmilus turkanae.* **Locality***:* Moruorot Hill and Rusinga, Kenya. **Absolute age estimate**: 17.8-16.8 Ma. *A. turkanae* is known from the African localities of Moruorot Hill and Rusinga (Kenya). Moruorot Hill, c. 17.5-16.8 Ma ^98^ is located west of Lake Turkana, while Rusinga is the same locality (and chronology) as mentioned above for *A. africanus*.

*Afrosmilus hispanicus.* **Locality***:* Artesilla*,* Spain. **Absolute age estimate**: 16.7-16.0 Ma. *A. hispanicus* is the only non-African originating species of *Afrosmilus*, known only from the Spanish locality of Artesilla, c. 16.7 Ma ^22,28^, in the northeast of the country in the province of Saragossa. *A. hispanicus* is typically correlated to the MN 4 zone ^23,26^, thus the younger date of 16.0 Ma is applied to its range, being the boundary between MN 4 and 5 ^95^.

*Maofelis cantonensis.* **Locality***:* Maoming Basin, Guangdong Province, China. **Absolute age estimate**: 41.03-33.9 Ma. The holotype, and only known specimen, is from the Youganwo Formation, middle-upper Eocene ^33^. The vertebrate fauna is only known from the upper portion of the formation containing dark oil shales and alternating mudstones ^33^. The precise stratigraphic horizon of the holotype is unknown, but based on magnetostratigraphy it should correlate to the Bartonian through Priabonian ^99^.

*Eofelis edwardsii.* **Locality**: France, Mongolia. **Absolute age estimate**: 35.37-30.83 Ma.

The vast majority of material referred to *Eofelis edwardsii* comes from the old collections of the phosphorites of Quercy with no precise age ^35^. However, material questionably referred to this taxon has been described from Villebramar, France, MP 22 ^35^. Egi et al. ^18^ described *Eofelis sp*. from the Ergilin Dzo Formation, Mongolia, which based on their measurements most closely resembles *Eofelis edwardsii* compared to the larger *E. giganteus* ^35^. The Mongolian specimens are dated to the Ergilian ALMA ^18^ and thus forms the oldest occurrence of this taxon while MP 22 the youngest for this study.

*Dinailurictis bonali.* **Locality***:* France, Spain. **Absolute age estimate**: 32.63-27.24 Ma

Most material attributed to this taxon comes from the Quercy fissures, dated MP 22 to 25, and Villebramar, MP 22 ^21,87^. The Spanish material, canine fragments, comes from Carrascosa del Campo, approximately MP 25 ^21^. Thus, the range of this taxon was chosen as MP 22-25, with absolute dates for these zones following Ogg et al. ^65^.

*Quercylurus major.* **Locality***:* France, Spain. **Absolute age estimate**: 28.82-27.24 Ma

The French material, including the holotype, come from the old collections of Quercy which have no precise age ^21^. The Spanish material from Carrascosa del Campo has an estimated age of MP 25 and thus the only reliably dated material^21^. Absolute dates follow Ogg et al. ^65^.

*Dinaelurus crassus.* **Locality***:* Oregon, USA. **Absolute age estimate**: 29.5-28.5 Ma. The precise stratigraphic context for the holotype, and only known specimen of this taxon, were unknown when Eaton ^37^ initially described it. However, a provisional Early Arikareean (Upper John Day Formation) was attributed to the specimen based on preservation and associated matrix ^37,39^. Albright et al. ^83^ suggest the origin to be the E unit of the Turtle Cove Member of the John Day Formation. This assessment is given without evidence, but is one of most fossiliferous units of the Turtle Cove Member. The associated absolute dates for the E unit were used for this study pending additional justification for the provenience of this taxon.

*Hoplophoneus oharrai*. **Locality***:* South Dakota, USA. **Absolute age estimate**: 35.7-34.7 Ma. The type and only specimen comes from the Crazy Johnson Member of the Chadron Formation in southwest South Dakota ^1^. Absolute dates are not available for the Crazy Johnson member, though it contains a middle Chadronian fauna (Ch3: 34.7-35.7 Ma ^100^) which is used for its range here.

*MA-PHQ 348*. **Locality***:* Phosphorites of Quercy, south-western France. **Absolute age estimate**: 47.4-23.03 Ma. This plesiomorphic nimravid specimen comes from the old collections of Quercy and thus has little to no stratigraphic context. The Phosphorites have a total extent from the base of MP 11 to the early Miocene ^101,102^. Given the poor understanding of this specimen’s stratigraphic context a broad prior from the base of MP 11 to the end of the Oligocene was chosen.

*Eusmilus dakotensis*. **Locality***:* South Dakota, USA. **Absolute age estimate**: 30.5-29.75 Ma. Three referable specimens come from the Brule Fm., Poleslide Member of South Dakota ^1^. Bryant ^39^ limits the first appearance to the latter Whitneyan, during Chron C12n or C11r. This leads to the FAD as stated with the LAD being the end of the Whitneyan.

*Eusmilus sicarius*. **Locality***:* South Dakota, USA. **Absolute age estimate**: 33.4-33.1 Ma. Following the results of this study, the only known specimen belonging to this species is the holotype, which comes from the lower nodule zone of the Scenic Member of the Brule Formation. This stratum is roughly correlative to Chron C13n ^81^.

*Eusmilus adelos*. **Locality***:* Niobrara County, Wyoming, USA. **Absolute age estimate**: 33.7-32.0 Ma. The holotype specimen comes from the Northwest corner of Seaman Hills, Niobrara County, Wyoming. The additional referred specimen was collected about one mile North of Whitman, Wyoming. Both of these specimens come from the Seaman Hills which spans the entirety of the Orellan in typical outcrops ^81,103^. Unfortunately, with no greater stratigraphic or geographic resolution, the entirety of the Orellan was used as the occurrence for this taxon.

*Eusmilus bidentatus.* **Locality**: France, Germany. **Absolute age estimate**: 33.77-32.63 Ma. This taxon is well known in the Phosphorites of Quercy, but also Soumailles ^43,45^. Additional material is known from southern Germany, but all of it (along with the French material) has been correlated to MP 21 ^43^.

*Eusmilus villebramarensis.* **Locality**: France, Switzerland, Germany. **Absolute age estimate**: 32.63-30.83 Ma. Most material for this taxon comes from France in the old collections of Quercy, but also Villebramar ^43^. However, occurrences in Switzerland and Germany are also known, which with the Villebramar material also has a MP 22 date ^43^.

**Custom Evolutionary Models (Rate Matrices)**

Beast 2.6.3 does not include a method to order characters from the BEAUti graphic user interface, thus only non-ordered characters are available for an analysis that does not manually edit the xml file. Below are schematic depictions of the rate matrices used in this analysis, with diagonal elements removed. The xml versions of these models are visible in Data S1.

*3-state Characters*

*Ordered*

Nimravidae_1_3_ordc_ratematrix

Characters: 1, 4, 10, 18, 23, 32, 53, 136, 140, 143, 146, 152-153, 155, 157, 161-162, 174, 184, 189, 193, 196, 206-207, 219


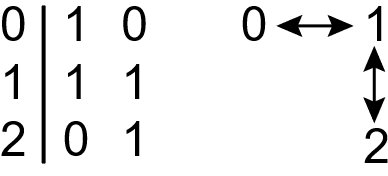


*Ordered, multipath*

Nimravidae_1_3m_ordc_ratematrix

Characters: 29, 135, 164


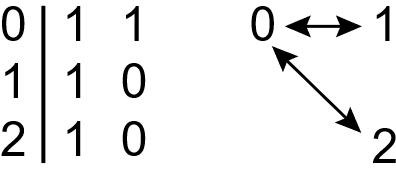


*4-state Characters*

*Ordered*

Nimravidae_1_4c_ord_ratematrix

Characters: 142, 148, 173, 175-176, 195, 212-213


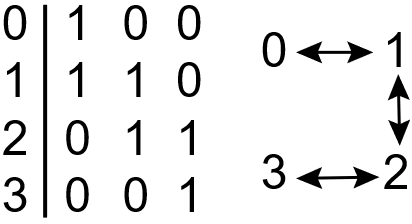


*Ordered, multipath 1, irreversible*

Nimravidae_1_4mi_1ord_ratematrix

Characters: 197


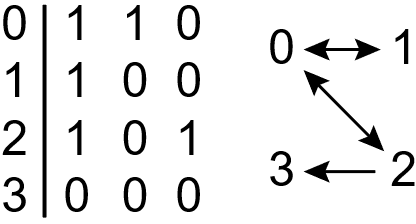


*Ordered, multipath 2*

Nimravidae_1_4m_2ord_ratematrix

Characters: 138


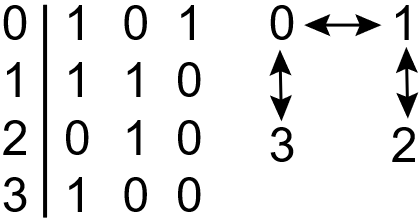


*Ordered, multipath 3*

Nimravidae_1_4m_3ord_ratematrix

Characters: 190


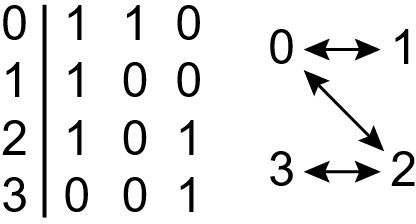


*5-state Characters*

*Ordered*

Nimravidae_1_5c_ord_ratematrix

Characters: 147, 191


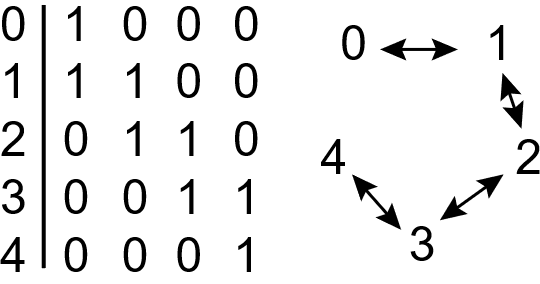


**Synapomorphies of the Nimravidae and Select Internal Clades**

Below are listed the unambiguous synapomorphies of the Nimravidae and internal clades:

**Nimravidae**

Char. 9: 1 🡪 0. Shape of mastoid process: forming a distinct process, extending out farther than paroccipital process, or subequal.

Char. 140: 0 🡪1. M1 roots: 2 roots.

Char. 154: 0 🡪 1. Basicranial foramina: veins merge extracranially, passing through the petrobasilar and posterior lacerate foramina respectively.

Char. 193: 0 🡪1. Incisors: mostly spatulate, I3 caniform.

Char. 200: 2 🡪 0. Anterior palatine canal opening location: level with P3.

**Nimravinae**

Char. 15: 1 🡪0. Anterior loop of internal carotid artery: lack of an anterior loop of the internal carotid artery.

Char. 83: 0 🡪 1. Carpus – proximal surface of scapholunar, curvature: convex and concave.

Char. 116: 1 🡪 0. Pes – calcaneus, cuboid facet shape: irregularly shaped, not round.

Char. 133: 0 🡪 1. Vagina processus hyoideus: slightly separate from stylomastoid foramen.

Char. 223: 0 🡪 1. Petrobasilar foramen: closed foramen in basioccipital.

**Hopliphoninae**

Char. 20: 1 🡪 2. Shape of the promontorium, anterior extension: blunt, quickly truncating.

Char. 85: 0 🡪 1. Carpus – cuneiform articulation with ulna, position: on distal surface of ulna.

Char. 200: 0 🡪 1. Anterior palatine canal opening location: anterior to P3.

**Barbourofelini + Nimravus/Dinaelurus**

Char. 20: 1 🡪 0. Shape of the promontorium, anterior extension: elongate, apron extension tapers to a point anteriorly, almond like in appearance.

Char. 148: 0 🡪 3. p4, anterior accessory cusp: larger than posterior.

Char. 192: 0 🡪 1. Mental fossa on the ventral face of the chin: fossa present and marked.

**Barbourofelini**

Char. 37: 0 🡪 1. Posterior accessory cusps on P3: two cusps present.

Char. 138: 1 🡪 0. p4, posterior accessory cusps: 2 cusps, linear.

Char. 153: 1 🡪 2. p3, posterior cusps: two cusps (posterior cingular cusp and posterior accessory cusp).

Char. 177: 0 🡪 1. C1 vertical grooves: present.

***Eusmilus***

Char. 197: 2 🡪 3. Loss of p3.

***Barbourofelis* + *Albanosmilus***

Char. 7: 0 🡪 2. Paroccipital process shape: absent.

Char. 173: 1 🡪 2. Relative width of p3 (ranked in order by ratio of Wp3 to Lp3): 0.61-0.66.

Char. 181: 0 🡪 1. Orbit closed.

Char. 184: 0 🡪 1. Ratio P4 length /p4 length: 1.95-2.1.

Char. 211: 0 🡪 1. Postorbital process of the jugal tall, triangular and tapering.


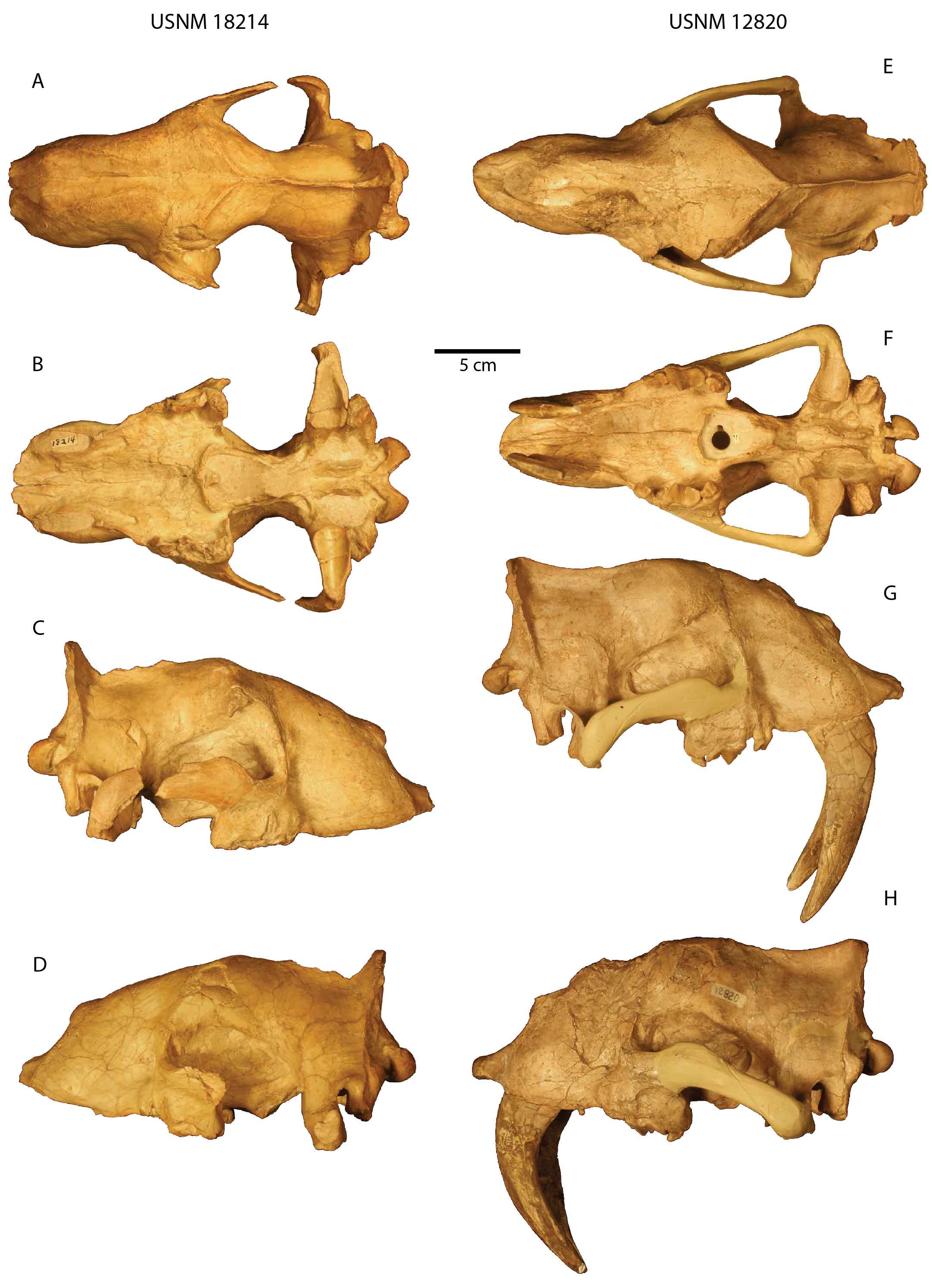


Figure S1. Crania of USNM 18214 (A-D) and USNM 12820 (E-H) in dorsal, ventral, right lateral, and left lateral respectively. Note that the tips of the canines of USNM 12820 are reconstructed such that actual length is likely exaggerated.


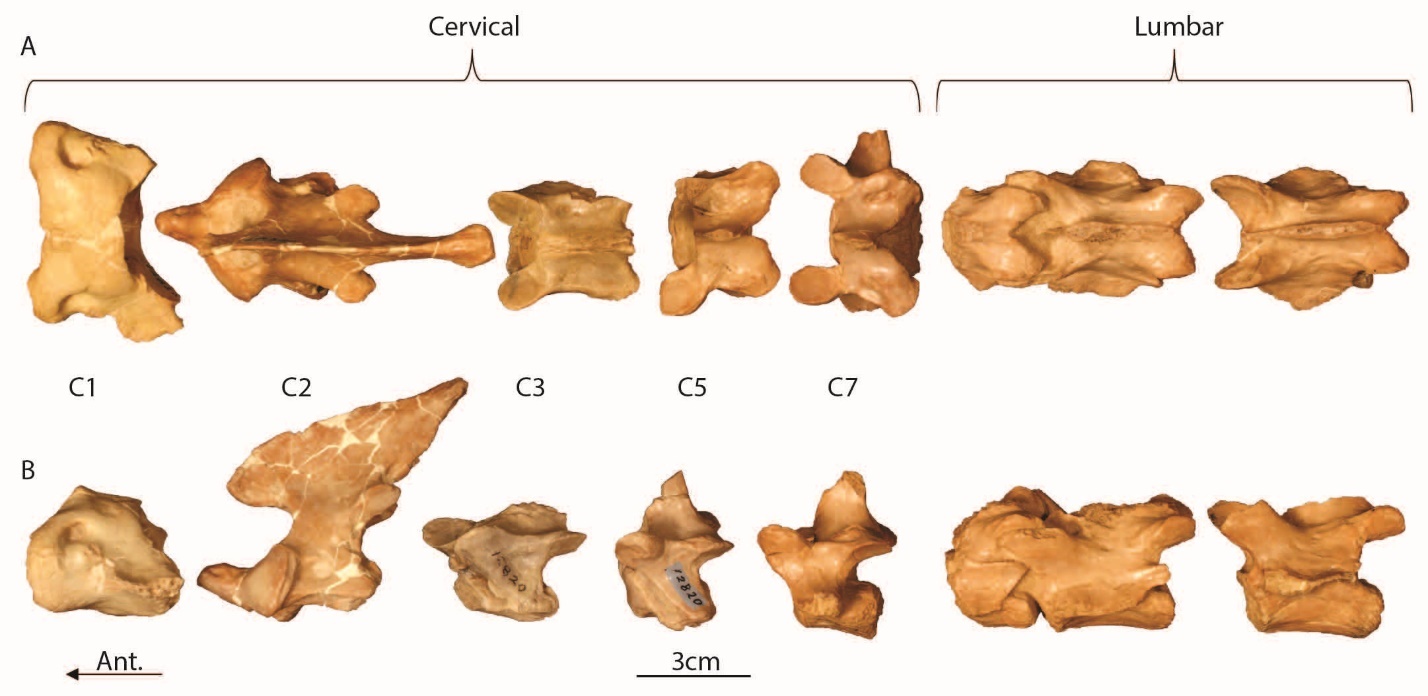


Figure S2. Dorsal (A) and left lateral (B) views of the preserved vertebral series for USNM 12820. Cervical vertebrae are identified as shown. Within the lumbar series three vertebrae are known, one partial, though their exact location cannot be determined, they were likely late (posterior) in the series.


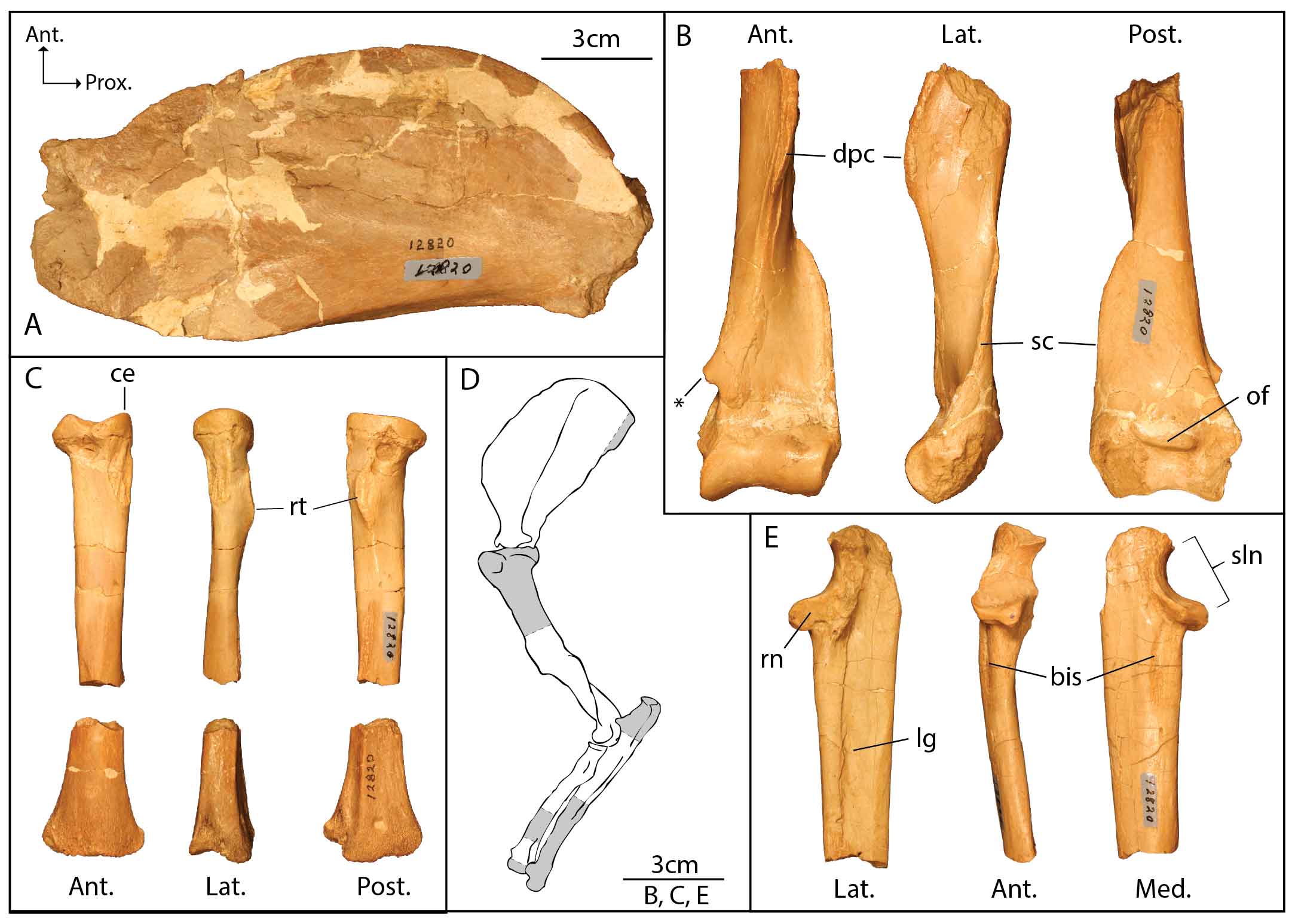


**Figure S3.** Preserved appendicular skeleton of USNM 12820, *Eusmilus adelos,* all left elements. **A**: scapula (medial view); **B**: distal humerus (anterior, lateral, posterior view); **C**: proximal and distal radius (anterior, lateral, posterior views); **D**: line drawing displaying estimated preserved portions of skeleton, missing in grey; **E**: proximal ulna (lateral, anterior, medial views). Note, loss of epiphyseal distal end of radius and proximal end of ulna. Abbreviations: bis = brachialis insertion site; ce = capitular eminence of radius; dpc = delto-pectoral crest; lg = lateral groove of ulna; of = olecranon fossa; rn = radial notch; rt = radial tuberosity; sc = supinator crest (brachial flange); sln = semilunar notch; * = remnants of bridge enclosing epicondylar foramen.


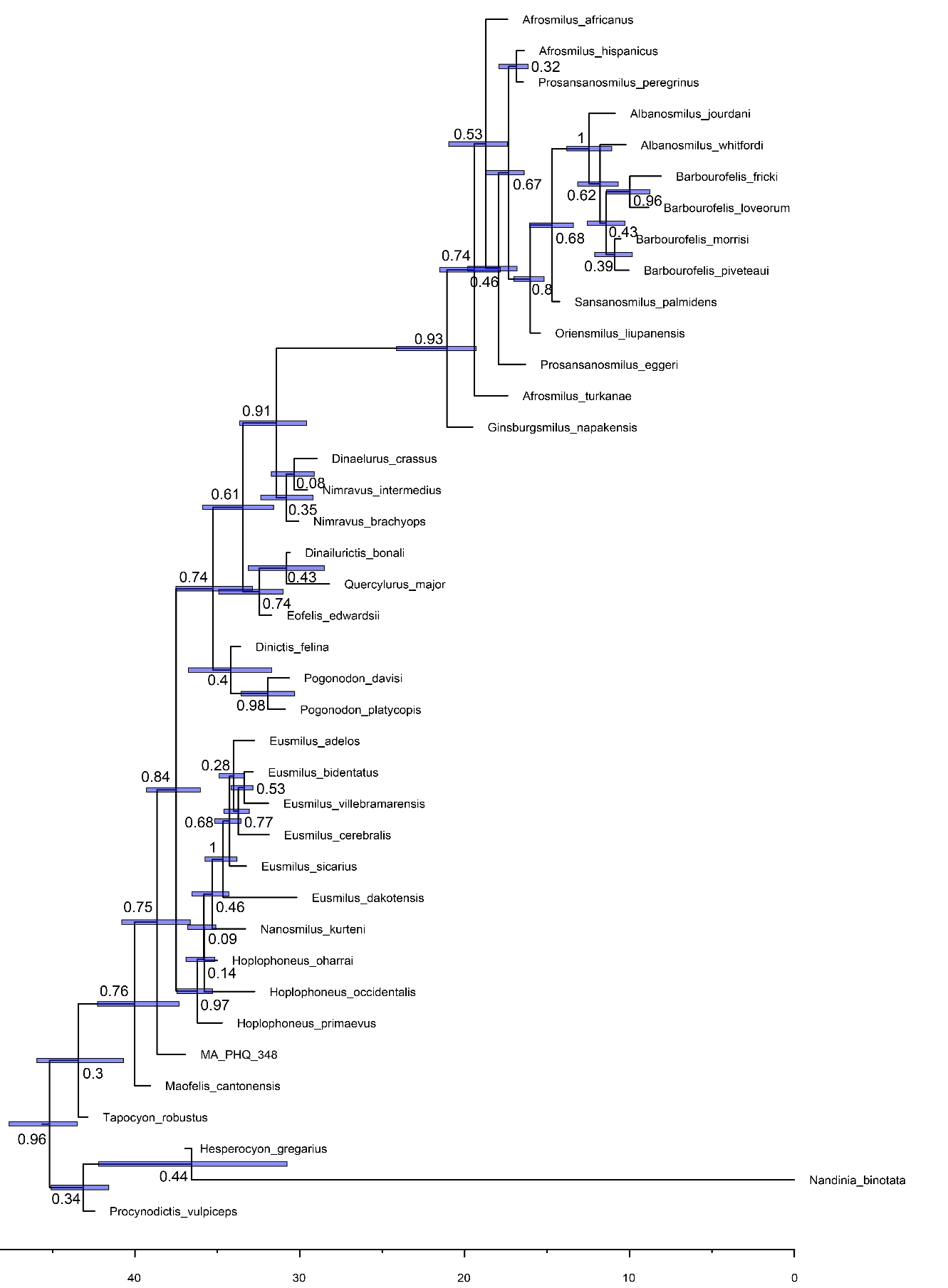


**Figure S4.** Maximum clade credibility tree of the best-supported Bayesian analysis. The numbers adjacent to nodes indicate posterior probability while the blue horizontal line segments indicate the 95% probability distribution of node ages. Scale is in million of years before the present.

**
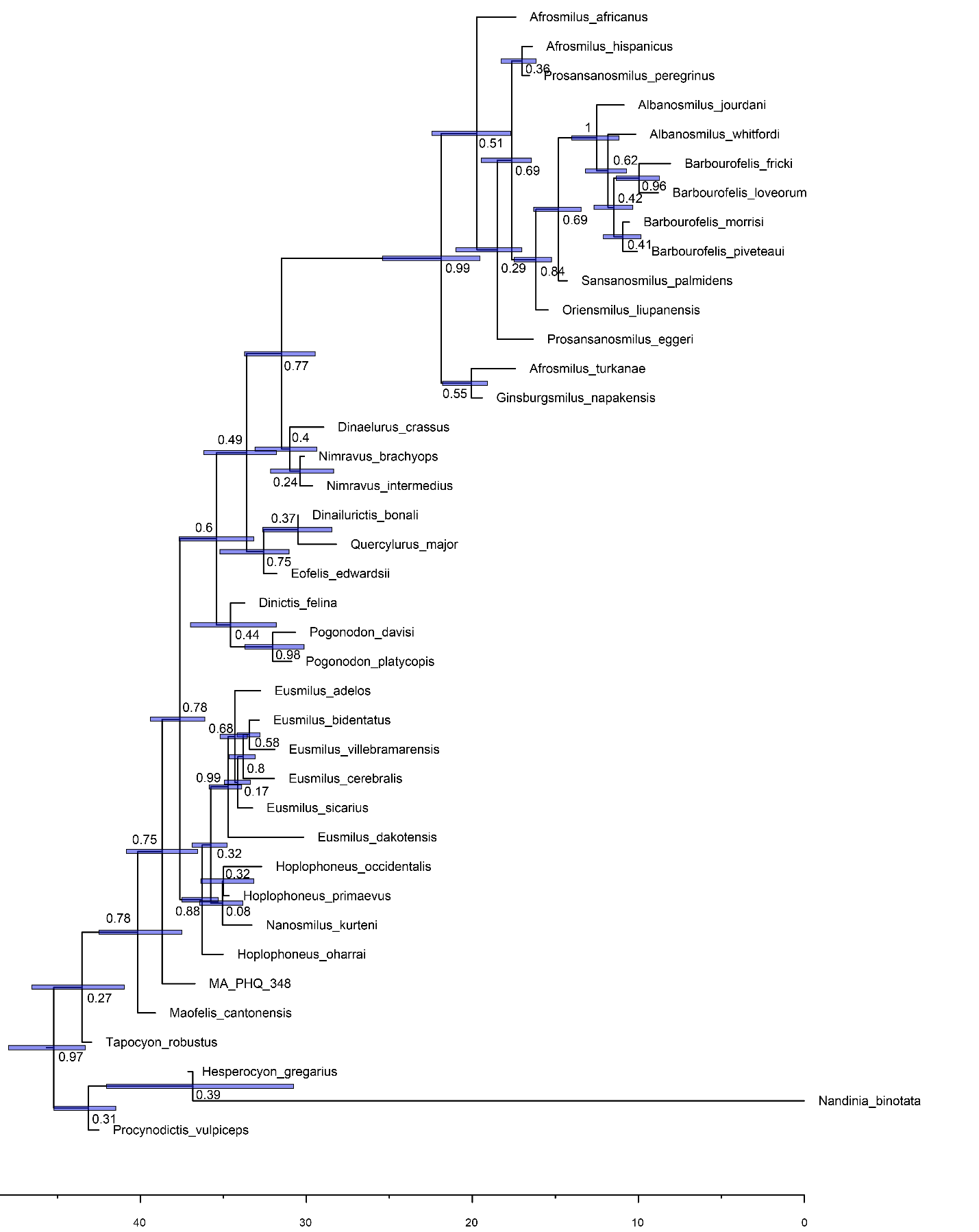
**

**Figure S5.** Maximum clade credibility tree of the best-supported Bayesian analysis with the exclusion of *Ginsburgsmilus napakensis* dentary and postcranial characters. The numbers adjacent to nodes indicate posterior probability while the blue horizontal line segments indicate the 95% probability distribution of node ages. Scale is in million of years before the present.

Data S1. All ten character partitioning and evolutionary model schemes assessed in this study, plus the morphology character matrix upon which they were built. The provide .zip file contains these schemes in .xml format as used by BEAST2 and the Path Sampler software, while the matrix is in .nex format.

Data S2. Required run files and output of the BayesTraits analysis of this study. The provided .zip file contains: The possible synapomorphies identified by TNT in matrix format, BayesTraits run file, 1,000 random post-burnin trees of the best-supported BEAST analysis, the MCC tree of the best-supported BEAST analysis, and mapped characters and associated node probabilities of all 41 possible synapomorphies.

**Supplemental Information References**

1. Barrett, P. Z. Taxonomic and systematic revisions to the North American Nimravidae (Mammalia, Carnivora). *PeerJ* **4:e1658**, (2016).

2. Bryant, H. N. & Russell, A. P. Carnassial functioning in nimravid and felid sabertooths: theoretical basis and robustness of inferences. in *Functional Morphology in Vertebrate Paleontology* (ed. Thomason, J.) 116–135 (Cambridge University Press, 1995).

3. Bryant, H. N. The anatomy, phylogenetic relationships and systematics of the Nimravidae (Mammalia: Carnivora). *Department of Zoology* **Doctoral d**, (University of Toronto, 1988).

4. Scott, W. B. & Jepsen, G. L. The mammalian fauna of the white river oligocene. *Trans. Am. Philos. Soc.* 108–153 (1936).

5. Wang, X., White, S. C. & Guan, J. A new genus and species of sabretooth , Oriensmilus liupanensis (Barbourofelinae, Nimravidae, Carnivora), from the middle Miocene of China suggests barbourofelines are nimravids, not felids. *J. Syst. Palaeontol.* **18**, 783–803 (2020).

6. Filhol, H. Études sur les Mammifères Fossiles de Sansan. *Bibliothèque des Hautes Études Sect. des Sci. Nat.* **37**, 1–319 (1890).

7. Ginsburg, L. La faune des carnivores miocènes de Sansan (Gers). *Mémoires du Muséum Natl. d’Histoire Nat. Sér. C - Sci. la Terre* **9**, 1–190 (1961).

8. Peigné, S. Les Carnivora de Sansan. in *Mammifères de Sansan* (ed. S., S. E. N.) 559–660 (Muséum national d’Histoire naturelle, 2012).

9. Morlo, M., Peigné, S. & Nagel, D. A new species of Prosansanosmilus: implications for the systematic relationships of the family Barbourofelidae new rank (Carnivora, Mammalia). *Zool. J. Linn. Soc.* **140**, 43–61 (2004).

10. Schultz, C. B., Schultz, M. R. & Martin, L. D. A new tribe of saber-toothed cats (Barbourofelini) from the Pliocene of North America. *Bull. Univ. Nebraska State Museum* **9**, 1–31 (1970).

11. Joeckel, R. M. & Stavas, J. M. New Insights into the Cranial Anatomy of Barbourofelis fricki (Mammalia, Carnivora). *J. Vertebr. Paleontol.* **16**, 585–591 (1996).

12. Baskin, J. A. Barbourofelis (Nimravidae) and Nimravides (Felidae), with a Description of Two New Species from the Late Miocene of Florida. *J. Mammal.* **62**, 122–139 (1981).

13. Neff, N. A. The basicranial anatomy of the Nimravidae (Mammalia: Carnivora) : character analyses and phylogenetic inferences. *PhD. Thesis. Department of Biology* **Doctoral d**, (City University of New York, 1983).

14. Hunt, R. M. Evolution of the aeluroid Carnivora: significance of auditory structure in the nimravid cat Dinictis. *Am. Museum Novit.* **2886**, 1–74 (1987).

15. Bryant, H. N. Delayed Eruption of the Deciduous Upper Canine in the Sabertoothed Carnivore Barbourofelis lovei (Carnivora, Nimravidae). *J. Vertebr. Paleontol.* **8**, 298–306 (1988).

16. Bryant, H. N. Phylogenetic relationships and systematics of the Nimravidae (Carnivora). *J. Mammal.* **72**, 56–78 (1991).

17. Welsh, E., Boyd, C. A., Spearing, K. & Barrett, P. Z. Stratigraphic and taxonomic revision of a North American False Saber-toothed Cat Cub. *Proc. S. Dak. Acad. Sci.* **94**, 141–153 (2015).

18. Egi, N. *et al.* Taxonomic revisions on nimravids and small feliforms (Mammalia, Carnivora) from the Upper Eocene of Mongolia. *Hist. Biol.* **28**, 105–119 (2016).

19. Peigné, S. & De Bonis, L. Le premier crane de Nimravus (Mammalia, Carnivora) d’Eurasie et ses relations avec N. brachyops d’Amerique du Nord. *Rev. Paleobiolgie* **18**, 57–67 (1999).

20. Schmidt-Kittler, N. Raubtiere aus dem Jungtertiär Kleinasiens. *Palaeontogr. Abteilung A* **155**, 1–131 (1976).

21. Peigné, S. Systematic review of European Nimravinae (Mammalia, Carnivora, Nimravidae) and the phylogenetic relationships of Palaeogene Nimravidae. *Zool. Scr.* **32**, 199–229 (2003).

22. Morales, J., Salesa, M. J., Pickford, M. & Soria, D. A new tribe, new genus and two new species of Barbourofelinae (Felidae, Carnivora, Mammalia) from the early Miocene of East Africa and Spain. *Trans. R. Soc. Edinb. Earth Sci.* **92**, 97–102 (2001).

23. Morales, J. & Pickford, M. A new barbourofelid mandible (Carnivora, Mammalia) from the Early Miocene of Grillental-6, Sperrgebiet, Namibia. *Commun. Geol. Surv. Namibia* **18**, 113–123 (2018).

24. Morales, J., Pickford, M. & Salesa, M. J. Creodonta and Carnivora from the Early Miocene of the Northern Sperrgebiet, Namibia. *Mem. Geol. Surv. Namibia* **20**, 291–310 (2008).

25. Heizmann, E. P. J., Ginsburg, L. & Bulot, C. Prosansanosmilus peregrinus, ein neuer machairodontider Felide aus dem Miocän Deutschlands und Frankreichs. *Stuttgarter Beiträge zur Naturkd. B* **58**, 1–27 (1980).

26. Morlo, M. New remains of Barbourofelidae (Mammalia, Carnivora) from the Miocene of Southern Germany: implications for the history of barbourofelid migrations. *Beiträge zur Paläontologie* **30**, 339–349 (2006).

27. Belinchón, M. & Morales, J. Los carnívoros del Mioceno Inferior de Buñol (Valencia, España). *Rev. española Paleontol.* **4**, 3–8 (1989).

28. Azanza, B. *et al.* Les grands mammifères du Miocène inférieur d’Artesilla, bassin de Calatayud-Teruel (province de Saragosse, Espagne). *Bull. du Museum Natl. d’Histoire Nat. Sect. C Sci. la Terre Paleontol. Geol. Mineral.* **15**, 105–153 (1993).

29. Werdelin, L. & Peigné, S. Carnivora. in *Cenozoic Mammals of Africa* (eds. Werdelin, L. & Sanders, W. J.) 603–657 (University of California Press, 2010). doi:10.5252/g2016n2a4

30. Andrews, C. W. On the Lower Miocene vertebrates from British East Africa, collected by Dr. Felix Oswald. *Q. J. Geol. Soc. London* **70**, 163–186 (1914).

31. Savage, R. J. G. Fossil Mammal of Africa: The Miocene Carnivora of East Africa. *Bull. Br. Museum (Natural Hist.* **10**, 239–316 (1965).

32. Robles, J. M. *et al.* New craniodental remains of the barbourofelid Albanosmilus jourdani (Filhol, 1883) from the Miocene of the Valles-Penedes Basin (NE Iberian Peninsula) and the phylogeny of the Barbourofelini. *J. Syst. Palaeontol.* **11**, 993–1022 (2013).

33. Averianov, A., Obraztsova, E., Danilov, I., Skutschas, P. & Jin, J. First nimravid skull from Asia. *Sci. Rep.* **6**, 1–8 (2016).

34. Ginsburg, L. Révision taxonomique des Nimravini (Carnivora Felidae) de l’Oligocène des Phosphorites du Quercy. *Bull. du Muséum Natl. d’Histoire Nat. Paris* **4e série 1**, 35–49 (1979).

35. Peigné, S. A new species of Eofelis (Carnivora: Nimravidae) from the Phosphorites of Quercy, France. *Comptes Rendus l’Académie des Sci. Paris* **330**, 653–658 (2000).

36. de Bonis, L., Gardin, A. & Blondel, C. Carnivora form the early Oligocene of the ‘Phosphorites du Quercy’ in southwestern France. *Geodiversitas* **41**, 601–621 (2019).

37. Eaton, G. F. John Day Felidae in the Marsh Collection. *Am. J. Sci.* **4**, 425–452 (1922).

38. Peigné, S. A primitive nimravine skull from the Quercy fissures, France: implications for the origin and evolution of Nimravidae (Carnivora). *Zool. J. Linn. Soc.* **132**, 401–410 (2001).

39. Bryant, H. N. Nimravidae. in *The Terrestrial Eocene-Oligocene Transition in North America* (eds. Prothero, D. R. & Emry, R. J.) 453–475 (Cambridge University Press, 1996).

40. Hatcher, J. B. Discovery, in the Oligocene of South Dakota, of Eusmilus, a genus of sabre-toothed cats new to North America. *Am. Nat.* **29**, 1091–1093 (1895).

41. Morea, F. M. On the species of Hoplophoneus and Eusmilus (Carnivora, Felidae). *Department of Geology* **Master’s t**, (South Dakota School of Mines and Technology, 1975).

42. Joeckel, R. M., Peigné, S., Hunt Jr., R. M. & Skolnick, R. I. The auditory region and nasal cavity of Oligocene Nimravidae (Mammalia: Carnivora). *J. Vertebr. Paleontol.* **22**, 830–847 (2002).

43. Peigné, S. & Brunet, M. Une Nouvelle Espèce Du Genre Eusmilus (Carnivora: Nimravidae) De L’Oligocène (MP 22) D’Europe. *Geobios* **34**, 657–672 (2001).

44. Piveteau, J. Les Chats des Phosphorites du Quercy. *Ann. Paléontologie* **20**, 107–163 (1931).

45. Ringeade, M. & Michel, P. A propos de l’Eusmilus (Eusmilus bidentatus ringeadei Ringeade et Michel, 1994) de Soumailles, lieu-dit de la commune de Pardaillan, Canton de Duras (Lot et Garonne, France) : étude descriptive. *Paléo* **6**, 5–37 (1994).

46. Geraads, D. & Güleç, E. Relationships of Barbourofelis piveteaui (Ozansoy, 1965), a late miocene nimravid (Carnivora, Mammalia) from Central Turkey. *J. Vertebr. Paleontol.* **17**, 370–375 (1997).

47. Wesley-Hunt, G. D. & Flynn, J. J. Phylogeny of the Carnivora: basal relationships among the carnivoramorphans, and assessment of the position of ‘Miacoidea’ relative to Carnivora. *J. Syst. Palaeontol.* **3**, 1–28 (2005).

48. Spaulding, M. & Flynn, J. J. Phylogeny of the Carnivoramorpha: The impact of postcranial characters. *J. Syst. Palaeontol.* **10**, 653–677 (2012).

49. Wible, J. R. & Spaulding, M. On the cranial osteology of the African palm civet, Nandinia Binotata (Gray, 1830) (Mammalia, Carnivora, Feliformia). *Ann. Carnegie Museum* **82**, 1–114 (2013).

50. Wesley, G. D. & Flynn, J. J. a Revision of Tapocyon (Carnivoramorpha), Including Analysis of the First Cranial Specimens and Identification of a New Species. *J. Paleontol.* **77**, 769–783 (2003).

51. Wang, X. Phylogenetic systematics of the Hesperocyoninae (Carnivora, Canidae). *Bull. Am. Museum Nat. Hist.* **221**, (1994).

52. Wang, X. Transformation from plantigrady to digitigrady: functional morphology of locomotion in Hesperocyon (Canidae: Carnivora). *Am. Museum Novit.* 1–23 (1993).

53. Bryant, H. N. The Carnivora of the Lac Pelletier Lower Fauna (Eocene: Duchesnean), Cypress Hills Formation, Saskatchewan. *J. Paleontol.* **66**, 847–855 (1992).

54. Gaubert, P., Wozencraft, W. C., Cordeiro-Estrela, P. & Veron, G. Mosaics of Convergences and Noise in Morphological Phylogenies: What’s in a Viverrid-Like Carnivoran? *Syst. Biol.* **56**, 865–894 (2005).

55. Barrett, P. Z., Hopkins, S. S. B. & Price, S. A. How many sabretooths? Reevaluating the number of carnivoran sabretooth lineages with total-evidence Bayesian techniques and a novel origin of the Miocene Nimravidae. *J. Vertebr. Paleontol.* **e1923523**, (2021).

56. Werdelin, L. & Solounias, N. The Hyaenidae : taxonomy, systematics and evolution. *Foss. Strat.* **30**, 104 (1991).

57. Tseng, Z. J. & Wang, X. The first record of the late Miocene Hyaenictitherium hyaenoides Zdansky (Carnivora: Hyaenidae) in Inner Mongolia and an evaluation of the genus. *J. Vertebr. Paleontol.* **27**, 699–708 (2007).

58. Christiansen, P. Phylogeny of the great cats (Felidae: Pantherinae), and the influence of fossil taxa and missing characters. *Cladistics* **24**, 977–992 (2008).

59. Christiansen, P. Phylogeny of the sabertoothed felids (Carnivora: Felidae: Machairodontinae). *Cladistics* **29**, 543–559 (2013).

60. Salesa, M. J., Antón, M., Morales, J. & Peigné, S. Systematics and phylogeny of the small felines (Carnivora, Felidae) from the Late Miocene of Europe: a new species of Felinae from the Vallesian of Batallones (MN 10, Madrid, Spain). *J. Syst. Palaeontol.* **10**, 87–102 (2012).

61. Salles, L. O. Felid Phylogenetics: Extant Taxa and Skull Morphology (Felidae, Aeluroidea). *Am. Museum Novit.* **3047**, 67 (1992).

62. Sakamoto, M. & Ruta, M. Convergence and Divergence in the Evolution of Cat Skulls: Temporal and Spatial Patterns of Morphological Diversity. *PLoS One* **7**, e39752 (2012).

63. Rothwell, T. P. Phylogenetic systematics of North American Pseudaelurus (Carnivora, Felidae). *Am. Museum Novit.* **3403**, (2003).

64. Van Valkenburgh, B., Grady, F. & Kurtén, B. The Plio-Pleistocene cheetah-like cat Miracinonyx inexpectatus of North America. *J. Vertebr. Paleontol.* **10**, 434–454 (1990).

65. Ogg, J. G., Ogg, G. & Gradstein, F. M. *A Concise Geologic Time Scale*. (Elsevier, 2016).

66. Kelly, T. S., Murphey, P. C. & Walsh, S. L. New Records of Small Mammals From the Middle Eocene Duchesne River Formation, Utah, and Their Implications for the Uintan-Duchesnean North American Land Mammal Age Transition. *Paludicola* **8**, 208–251 (2012).

67. Robinson, P. *et al.* Wasatchian through duchesnean biochronology. in *Late Cretaceous and Cenozoic mammals of North America: Biostratigraphy and Geochronology* (ed. M. Woodburne) 106–155 (Columbia University Press, 2004).

68. Spaulding, M., Flynn, J. J. & Stucky, R. K. A new basal carnivoramorphan (Mammalia) from the ‘Bridger B’ (Black’s fork member, bridger formation, bridgerian nalma, middle eocene) of wyoming, USA. *Palaeontology* **53**, 815–832 (2010).

69. Solé, F., Smith, T., de Bast, E., Codrea, V. & Gheerbrant, E. New carnivoraforms from the latest Paleocene of Europe and their bearing on the origin and radiation of Carnivoraformes (Carnivoramorpha, Mammalia). *J. Vertebr. Paleontol.* **36**, e1082480 (2016).

70. Tomiya, S. & Tseng, Z. J. Whence the beardogs? Reappraisal of the middle to late eocene ‘miacis’ from Texas, USA, and the origin of amphicyonidae (mammalia, carnivora). *R. Soc. Open Sci.* **3**, (2016).

71. Nyakatura, K. & Bininda-Emonds, O. Updating the evolutionary history of Carnivora (Mammalia): a new species-level supertree complete with divergence time estimates. *BMC Biol.* **10**, 1–31 (2012).

72. Zhou, Y., Wang, S.-R. & Ma, J.-Z. Comprehensive species set revealing the phylogeny and biogeography of Feliformia (Mammalia, Carnivora) based on mitochondrial DNA. *PLoS One* **12**, e0174902 (2017).

73. Morales, J., Pickford, M. & Soria, D. Carnivores from the late miocene and basal pliocene of the Tugen Hills, Kenya. *Rev. la Soc. Geológica España* **18**, 39–61 (2005).

74. Qiu, Z.-X. *et al.* Neogene land mammal stages/ages of China – toward the goal to establish an Asian land mammal stage/age scheme. in *Fossil mammals of Asia: Neogene biostratigraphy and chronology* (eds. X. Wang, Flynn, L. J. & Fortelius, M.) 29–90 (Columbia University Press, 2013).

75. Wang, S.-Q. *et al.* Biostratigraphic subdividing of the Neogene Dingjia’ergou mammalian fauna, Tongxin County, Ningxia Province, and its background for the uplift of the Tibetan Plateau36. *Quat. Sci.* **36**, 789–809 (2016).

76. Ginsburg, L. Les faunes de mammifères terrestres du Miocène moyen des Faluns du bassin de Savigné-sur-Lathan (France). *Geodiversitas* **23**, 381–394 (2001).

77. Tseng, Z. J., Takeuchi, G. T. & Wang, X. Discovery of the upper dentition of Barbourofelis whitfordi (Nimravidae, Carnivora) and an evaluation of the genus in California. *J. Vertebr. Paleontol.* **30**, 244–254 (2010).

78. Kappelman, J. *et al.* Chronology. in *Geology and paleontology of the Miocene Sinap Formation, Turkey* (eds. Fortelius, M., Kappelman, J., Sen, S. & Bernor, R.-L.) 41–66 (Columbia University Press, 2003).

79. Koufos, G. D., Mayda, S. & Kaya, T. *New carnivoran remains from the Late Miocene of Turkey*. *PalZ* **92**, (Springer Berlin Heidelberg, 2018).

80. Famoso, N. A., Samuels, J. X., Hopkins, S. S. B., Emery, M. M. & Davis, E. B. Updated Biostratigraphy of the Turtle Cove Member (John Day Formation) in the John Day Basin, Oregon. in *Western association of vertebrate Paleontology annual Meeting: Program with abstracts. PaleoBios 32(1)* **32**, 6 (2015).

81. Prothero, D. R. & Emry, R. J. The Chadronian, Orellan, and Whitneyan North American land mammal ages. in *Late Cretaceous and Cenozoic Mammals of North America: Biostratigraphy and Geochronology* (ed. Woodburne, M. O.) 156–168 (Columbia University Press, 2004).

82. Bryant, H. N. & Fremd, T. J. Revised Biostratigraphy of the Nimravidae (Carnivora) from the John Day Basin of Oregon. *J. Vertebr. Paleontol.* **18**, 30A (1998).

83. Albright, L. B. *et al.* Revised chronostratigraphy and biostratigraphy of the John Day formation (Turtle Cove and Kimberly Members), Oregon, with implications for updated calibration of the Arikareean North American land mammal age. *J. Geol.* **116**, 211–237 (2008).

84. Boyd, C. A. & Webster, J. R. Depositional History of the Chadron Formation in North Dakota. *North Dakota Geol. Surv. Rep. Investig.* **120**, 107 (2018).

85. Martin, L. D. A new miniature saber-tooth nimravid from the Oligocene of Nebraska. *Ann. Zool. Fennici* **28**, 341–348 (1992).

86. Zanazzi, A., Kohn, M. J. & Terry, D. O. Biostratigraphy and paleoclimatology of the Eocene-Oligocene boundary section at Toadstool Park (northwestern Nebraska). *Geol. Soc. Am. Spec. Pap.* **452**, 197–214 (2009).

87. Peigné, S., Vianey-Liaud, M., Pélissié, T. & Sigé, B. Valbro: A new site of vertebrates from the early Oligocene (MP22) of France (Quercy). I – Geological context; Mammalia: Rodentia, Hyaenodontida, Carnivora. *Ann. Paleontol.* **100**, 1–45 (2014).

88. Gromova, V. Premiere decoverte d’un chat primitif au Paleogene d’Asie Centrale. *Vertebr. Palasiat.* **3**, 59–72 (1959).

89. Dashzeveg, D. Some Carnivorous Mammals from the Paleogene of the Eastern Gobi Desert , Mongolia , and the Application of Oligocene Carnivores to Stratigraphic Correlation. *Am. Museum Novit.* **3179**, 1–14 (1996).

90. Toohey, L. The Species of Nimravus (Carnivora, Felidae). **Doctoral d**, (Princeton University, 1959).

91. Peigné, S., Chaimanee, Y., Jaeger, J.-J., Suteethorn, V. & Ducrocq, S. Eocene nimravid carnivorans from Thailand. *J. Vertebr. Paleontol.* **20**, 157–163 (2000).

92. Werdelin, L. Chronology of Neogene Mammal Localities. in *Cenozoic Mammals of Africa* (eds. Werdelin, L. & Sanders, W. J.) 27–43 (University of California Press, 2010).

93. Moser, M., Rössner, G. E., Göhlich, U. B., Böhme, M. & Fahlbusch, V. The fossil lagerstätte Sandelzhausen (Miocene; southern Germany): History of investigation, geology, fauna, and age. *Palaontologische Zeitschrift* **83**, 7–23 (2009).

94. Reichenbacher, B. *et al.* A new magnetostratigraphic framework for the Lower Miocene A new magnetostratigraphic framework for the Lower Miocene ( Burdigalian / Ottnangian , Karpatian ) in the North Alpine Foreland Basin. *Swiss J. Geosci.* **106**, 309–334 (2013).

95. Agustí, J. *et al.* A calibrated mammal scale for the Neogene of Western Europe. State of the art. *Earth Science Reviews* **52**, 247–260 (2001).

96. Anyonge, W. Fauna from a New Lower Miocene Locality West of Lake Turkana, Kenya. *J. Vertebr. Paleontol.* **11**, 378–390 (1991).

97. Drake, R. E., Van Couvering, J. A., Pickford, M. H., Curtis, G. H. & Harris, J. A. New chronology for the Early Miocene mammalian faunas of Kisingiri, Western Kenya. *J. Geol. Soc. London* **145**, 479–491 (1988).

98. Boschetto, H. B., Brown, F. H. & McDougall, I. M. Stratigraphy of the Lothidok Range, northern Kenya, and K/Ar ages of its Miocene primates. *J. Hum. Evol.* **22**, 47–71 (1992).

99. Li, Y. X. *et al.* Terrestrial responses of low-latitude Asia to the Eocene-Oligocene climate transition revealed by integrated chronostratigraphy. *Clim. Past* **12**, 255–272 (2016).

100. Janis, C. M., Gunnell, G. F. & Uhen, M. D. *Evolution of Tertiary mammals of North America. Volume 2: Small mammals, xenarthrans, and marine mammals*. (Cambridge University Press, 2008).

101. Legendre, S. *et al.* La faune de mammifères de Vielase (phosphorites du Quercy, Sud de la France): Preuve paléontologique d’une karstification du Quercy dès l’Eocène inférieur Par Montpellier ; Toulouse Débutées en 1965 , l ’ exploration et l ’ exploitation paléontologique no. *Neues Jahrb. für Geol. und Paläontologie* **7**, 414–428 (1992).

102. Sigé, B., Aguilar, J. P., Astruc, J. G. & Marandat, B. Extension au Miocène inférieur des remplissages phosphatés du Quercy. La faune de Vertébrés de Crémat (Lot, France). *Geobios* **24**, 497–502 (1991).

103. Prothero, D. R. & Whittlesey, K. E. Magnetic stratigraphy and biostratigraphy of the Orellan and Whitneyan land-mammal ‘ages’ in the White River Group. *Geol. Soc. Am. Spec. Pap.* **325**, 39–61 (1998).
